# Supplementary material for: Programmable Assembly of Multistranded Helices in Water
Source: Nat Commun. 2025 Dec 11;16:10955. doi: 10.1038/s41467-025-67227-0 (PMC12698662; doi:10.1038/s41467-025-67227-0)
Supplement: Supplementary file 1 — Supplementary Information [file 41467_2025_67227_MOESM1_ESM.pdf]

Supplementary Information for:

Programmable Assembly of Multistranded Helices in Water

Dimitri Delcourt,[1] Reguram Arumugaperumal,[1] Prachi Verma,[1] Perttu Permi,[1,2] Rosa M. Gomila,[3] Antonio Frontera,[3] and Fabien B. L. Cougnon[1*]

[1] Department of Chemistry, Nanoscience Center, University of Jyväskylä, P.O. Box 35, FI-40014 JYU, Finland

[2] Department of Biological and Environmental Science, Nanoscience Center, University of Jyväskylä, P.O. Box 35, FI-40014 JYU, Finland

[3] Department de Química, Universitat de les Illes Balears, Carretera de Valldemossa km 7.5, 07122 Palma de Mallorca, Baleares, Spain

Corresponding Author

* fabien.b.l.cougnon@jyu.fi

Table of Contents

1.	General information	3
2.	Synthesis of the strands	5
3.	NMR characterization of unfolded strand **1** in CD_3CN	18
4.	NMR characterization of double helix (**1**)$_2$ in D_2O	22
5.	Host-guest studies with double helix (**1**)$_2$·**TFA**	29
6.	NMR characterization of unfolded strand **2** in CD_3CN	32
7.	NMR characterization of double helix (**2**)$_2$ and triple helix (**2**)$_3$ in D_2O	36
8.	Host-guest studies with triple helix (**2**)$_3$·**TFA**	46
9.	Concentration- and temperature-dependent double-to-triple helix transformation in the presence of guests **G1-G4**	51
10.	^{1}H and ^{13}C NMR spectra of the synthesized compounds	57
11.	DFT calculations	66
12.	References	67

1. General information

All reagents and solvents were purchased from commercial sources and used without further purification – except triethylamine, which was distilled on potassium hydroxide under inert atmosphere before being used. Reagents were purchased from Sigma-Aldrich [(triisopropylsilyl)acetylene, trimethylsilylacetylene, triethylamine, bis(triphenylphosphine)palladium(II) dichloride, tetrakis(triphenylphosphine)palladium(0) , methyl trifluoromethanesulfonate, potassium perfluorobutanesulfonate, potassium trifluoromethanesulfonate] and Tokyo Chemical Industry Co [3,5-dibromopyridine, 3-ethynylpyridine, copper(I) iodide, tetrabutylammonium fluoride solution (1 M in tetrahydrofuran), 3-bromoiodobenzene, 1,3-diiodobenzene, 1,3-diethynylbenzene, sodium 1-butanesulfonate, 4,4'-biphenyldisulfonic acid].

NMR analyses. Spectra were measured on a JEOL 400 MHz NMR ECX-400 spectrometer equipped with a 5-mm broadband 40TH5AT/FG2 autotunable universal probe, a 400 MHz Bruker Avance III NMR spectrometer equipped with a 5-mm BBFO probehead, a Bruker Avance III 500 MHz NMR spectrometer equipped with a 5-mm nitrogen-cooled ^{1}H, ^{13}C, ^{15}N Prodigy probehead and a Bruker Avance III HD 800 MHz NMR spectrometer, equipped with a cryogenically cooled, 5 mm ^{1}H, ^{13}C, ^{15}N triple-resonance TCI probehead. All signals were internally referenced to the solvent residue ($CDCl_3$: 7.26 ppm, D_2O: 4.79 ppm, CD_3CN: 1.96 ppm) or tetramethylsilane. NMR solvents ($CDCl_3$ with or without 0.03% of tetramethylsilane, D_2O and CD_3CN) were purchased from Sigma-Aldrich and Eurisotop.

HR-MS analyses. HR-MS analyses were performed on an Agilent 6560 ESI-IM-QTOF mass spectrometer equipped with AJS ESI ion source.

UHPLC-MS analyses. UHPLC-MS analyses were performed on an Agilent 6530-QTOF mass spectrometer (ionisation mode: ESI+) equipped with an Agilent 1290 UHPLC inlet, UV detector and Autosampler. Eluents: solution A (99.9% water, 0.1 % trifluoroacetic acid), solution B (100% acetonitrile). Gradient: 0 min, 5% B; 1 min, 5% B; 10 min, 100% B. Flow rate: 0.5 mL/min.

Melting Point. Melting points were measured on a Büchi Melting point B-540/B-545.

Preparative HPLC. Water soluble compounds were purified by HPLC Shimadzu LC-8A system equipped with a Shimadzu array detector SPD-M20A using a Gemini 10 µm C18 column 110 Å, 100 x 21.20 mm 10 micron from Phenomenex. Eluents: solution A (99.9% water, 0.1% trifluoroacetic acid), solution B (100% acetonitrile). Gradient: 0 min, 5% B; 1 min, 5% B; 15 min, 100% B. Flow rate: 12 mL/min.

List of abbreviations. DOSY: Diffusion-ordered spectroscopy; DMF: N,N-Dimethylformamide, equiv.: equivalents; ESI-IM-QTOF: Electrospray Ionisation – Ion Mobility – Quadrupole Time-of-Flight; EXSY: Exchange spectroscopy; HR-MS: High-Resolution Mass Spectrometry; NMR: Nuclear Magnetic Resonance; OTf: Triflate; ROESY: rotating frame Overhauser effect spectroscopy; TBAF: Tetra-*n*-butylammonium fluoride; TFA: Trifluoroacetic acid; TIPS: Triisopropylsilyl; TMS: Trimethylsilyl; UHPLC: Ultra-High Performance Liquid Chromatography; UV: Ultraviolet.

2. Synthesis of the strands

2.1. Synthesis of strand 1

Strand **1** was synthesized according to the following procedure:

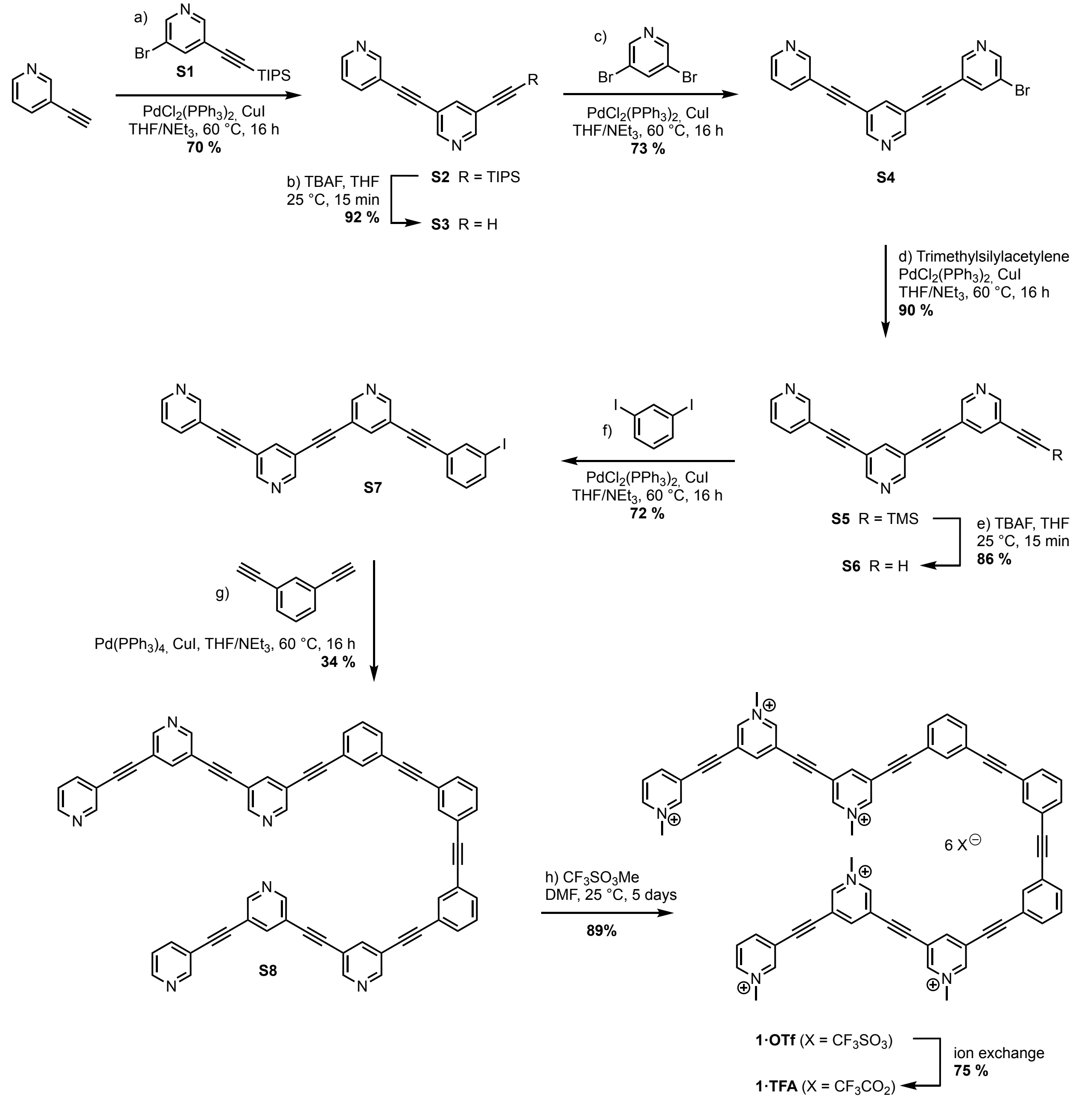

Compound S1

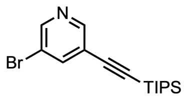

The synthesis of **S1** was adapted from a published procedure.[1] 3,5-Dibromopyridine (1.0 g, 4.2 mmol), (triisopropylsilyl)acetylene (1.0 mL, 4.7 mmol), $PdCl_2(PPh_3)_2$ (190 mg, 0.06 mmol) and CuI (23 mg, 0.03 mmol) were dissolved in triethylamine (10 mL) under nitrogen atmosphere. The mixture was stirred 16 h at 50 °C and concentrated by rotary evaporation. The crude product was redissolved in dichloromethane, washed with a saturated solution of Na_2CO_3, dried over Na_2SO_4, filtered, and concentrated by rotary evaporation. The residue was purified by silica gel column chromatography (gradient pentane/ethyl acetate = 100/0 to 30/70) to yield **S1** (1.4 g, 97 %) as a yellow oil. Spectral data closely match those previously reported in the literature.[1] ^{1}H NMR (400 MHz, $CDCl_3$ + 0.03 % tetramethylsilane, 293 K): δ 8.58 (br s, 2H), 7.87 (dd, *J* = 2.2, 1.8 Hz, 1H), 1,12 (s, 21H). ^{13}C NMR (100 MHz, $CDCl_3$ + 0.03 % TMS, 293 K): δ 150.7, 149.7, 141.1, 122.0, 120.0, 101.7, 96.8, 18.6, 11.2.

Compound S2

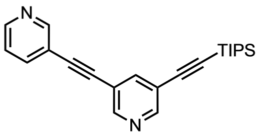

3-Ethynylpyridine (242 mg, 2.4 mmol), **S1** (783 mg, 2.3 mmol), $PdCl_2(PPh_3)_2$ (84 mg, 0.1 mmol) and CuI (12 mg, 0.06 mmol) were dissolved in triethylamine (3 mL) and tetrahydrofuran (3 mL) at 0 °C under nitrogen atmosphere. The reaction mixture was stirred 16 h at 60 °C, concentrated by rotary evaporation, and the resulting residue was purified by silica gel column chromatography (gradient pentane/ethyl acetate = 100/0 to 50/50) to yield **S2** (581.0 mg, 70 %) as a colorless oil. Melting point: 67-68 °C. ^{1}H NMR (400 MHz, $CDCl_3$ + 0.03 % tetramethylsilane, 293 K): δ 8.78 (br d, *J* = 1.7 Hz, 1H), 8.67 (d, *J* = 2.1 Hz, 1H), 8.64 (d, *J* = 2.1 Hz, 1H), 8.59 (dd, *J* = 4.9, 1.7 Hz, 1H), 7.90 (t, *J* = 2.1 Hz, 1H), 7.82 (dt, *J* = 8.1, 1.7 Hz, 1H), 7.32 (ddd, *J* = 8.1, 4.9, 0.8 Hz, 1H), 1.13 (m, 21H). ^{13}C NMR (100 MHz, $CDCl_3$ + 0.03 % TMS, 293 K): δ 152.4, 151.9, 150.9, 149.3, 141.3, 138.7, 123.3, 120.5, 119.6, 119.4, 102.4, 96.2, 89.8, 88.5, 18.7, 11.3. HR- HR-MS (ESI+) *m/z* calculated for $C_{23}H_{28}N_2Si^+$ $[M]^+$ = 361.2024, found 360.2022, Δ = -0.58 ppm.

Compound S3

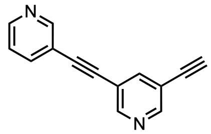

Tetrabutylammonium fluoride (1 M in tetrahydrofuran, 720 µL) was added to a solution of **S2** (795 mg, 2.2 mmol) in tetrahydrofuran (150 mL). The reaction mixture was stirred for 15 min at 25 °C and concentrated by rotary evaporation. The residue was purified by silica gel column chromatography (gradient pentane/ethyl acetate = 100/0 to 60/40) to yield **S3** (403 mg, 92 %) as a white solid. Melting point: 107-108 °C. ^{1}H NMR (400 MHz, $CDCl_3$ + 0.03 % tetramethylsilane, 293 K): δ 8.78 (br s, 1H), 8.70 (d, *J* = 2.0 Hz, 1H), 8.66 (d, *J* = 2.0 Hz, 1H), 8.60 (br d, *J* = 5.0 Hz, 1H), 7.91 (t, *J* = 2.0 Hz, 1H), 7.82 (dt, *J* = 7.8, 2.0 Hz, 1H), 7.32 (dd, *J* = 7.8, 5.0 Hz, 1H), 3.26 (s, 1H). ^{13}C NMR (100 MHz, $CDCl_3$ + 0.03 % tetramethylsilane, 293 K): δ 152.4, 151.9, 151.5, 149.4, 141.4, 138.7, 123.3, 119.5, 119.2, 90.0, 88.2, 81.6, 79.5. HR-MS (ESI+) *m/z* calculated for $C_{14}H_9N_2$ $[M]^+$ = 204.0687, found 204.0695, Δ = -3.76 ppm.

Compound S4

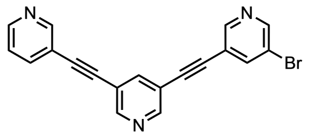

Compound **S3** (127 mg, 0.6 mmol), 3,5-dibromopyridine (966 mg, 4,1 mmol), $PdCl_2(PPh_3)_2$ (72 mg, 0.1 mmol) and CuI (11 mg, 0.03 mmol) were dissolved in dry triethylamine (10 mL) and tetrahydrofuran (10 mL) at 0 °C under nitrogen atmosphere. The reaction mixture was stirred for 8 h at 25 °C and 16 h at 60 °C before being concentrated by rotary evaporation. The crude product was redissolved in dichloromethane, washed with a saturated solution of Na_2CO_3, dried over Na_2SO_4, filtered, and concentrated by rotary evaporation. The residue was purified by silica gel column chromatography (gradient pentane/ethyl acetate = 100/0 to 0/100) to yield **S4** (481 mg, 73 %) as a white solid. Melting point: 189-190 °C. ^{1}H NMR (400 MHz, $CDCl_3$ + 0.03 % tetramethylsilane, 293 K): δ 8.79 (br s, 1H), 8.74 (d, *J* = 2.0 Hz, 1H), 8.72 (d, *J* = 2.0 Hz, 1H), 8.68 (d, *J* = 2.0 Hz, 1H), 8.66 (d, *J* = 2.0 Hz, 1H), 8.61 (br d, *J* = 4.9 Hz, 1H), 7.98 (t, *J* = 2.0 Hz, 1H), 7.97 (t, *J* = 2.0 Hz, 1H), 7.83 (dt, *J* = 7.9, 2.0 Hz, 1H), 7.33 (dd, *J* = 7.9, 4.9Hz, 1H). ^{13}C NMR (100 MHz, $CDCl_3$ + 0.03 % tetramethylsilane, 293 K): δ 152.5, 151.7, 151.4, 150.6, 150.4, 149.5, 140.9, 138.7, 123.3, 120.9, 120.3, 119.7, 119.5, 119.2, 90.3, 89.5, 88.2. HR-MS (ESI+) *m/z* calculated for $C_{14}H_{11}BrN_3$ $[M]^+$ = 359.0058, found 359.0062, Δ = -1.19 ppm.

Compound S5

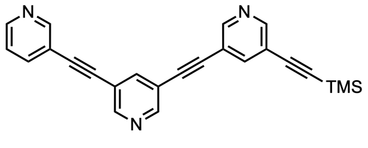

Compound **S4** (1.90 g, 5.27 mmol, 1.00 equiv.), $PdCl_2(PPh_3)_2$ (0.185 g, 0.263 mmol, 0.05 equiv.), CuI (50 mg, 0.263 mmol, 0.05 equiv.) in dry degassed tetrahydrofuran (25 mL) at 0 °C was added trimethylsilylacetylene (1.12 mL, 7.91 mmol, 1.5 equiv.) and Et_3N (2.5 mL, 15.82 mmol, 3.00 equiv.). The resulting mixture was then allowed to stir overnight at 60 °C and monitored by thin-layer chromatography. After completion, the reaction mixture was filtered through celite. The resulting organic phase was diluted in dichloromethane (50 mL) and washed with a saturation solution of NH_4Cl. The combined organic layers were washed with brine (100 mL), dried (Na_2SO_4), filtered and concentrated. The residue was purified by flash chromatography (hexane/ethyl acetate = 30/70) to give product **S5** (1.79 g, 90%) as a white solid. Melting point: 188-190 °C. ^{1}H NMR (500 MHz, $CDCl_3$, 298 K): δ 8.80 (s, 1H), 8.74 (dd, *J* = 2.0 , 2.0 Hz, 2H), 8.68 (dd, *J* = 2.0 Hz, 2H), 8.62 (d, *J* = 4.8 Hz, 1H), 7.98 (br d, *J* = 2.0 Hz, 1H), 7.91 (br d, *J* = 2.0 Hz, 1H), 7.86-7.84 (m, 1H), 7.34 (dd, *J* = 4.7, 4.9 Hz, 1H), 0.28 (s, 9H). ^{13}C NMR (126 MHz, $CDCl_3$, 298 K): δ 152.37, 151.86, 151.42, 151.28, 150.94, 149.36, 141.18, 140.77, 138.62, 123.16, 120.14, 119.59, 119.44, 119.33, 118.97, 100.29, 99.64, 90.08, 89.17, 88.73, 88.16, -0.25. HR-MS (ESI+) *m/z* calculated for $C_{24}H_{19}N_3Si^+$ $[M+H]^+$ = 378.1426, found 378.1424, Δ = -1.56 ppm.

Compound S6

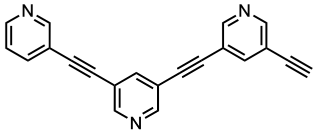

To a stirred solution of substrate **S5** (1.75 g, 4.63 mmol, 1.00 equiv.) in tetrahydrofuran/methanol (1:2, 30 mL) was added K_2CO_3 (1.27 g, 9.27 mmol, 2.00 equiv.). The resulting mixture was stirred at room temperature for 15 min at 25 °C and monitored by thin-layer chromatography. After completion, the reaction mixture was filtered, concentrated and subjected to column chromatography (hexane/ethyl acetate = 20/80) to give product **S6** as a white solid (1.216 g, 86%). Melting point: 189-190 °C. ^{1}H NMR (500 MHz, $CDCl_3$, 298 K): δ 8.81 (br d, *J* = 2.0 1H), 8.76-8.74 (m, 3H), 8.70 (d, *J* = 2.0 Hz, 1H), 8.62 (dd, *J* = 2.0, 2.0 Hz, 1H), 8.0 (br t, *J* = 2.0 Hz, 1H), 7.94 (br t, *J* = 2.0 Hz, 1H), 7.87-7.85 (m, 1H), 7.35 (dd, *J* = 4.0, 5.0 Hz, 1H), 3.29 (s, 1H). ^{13}C NMR (126 MHz, $CDCl_3$, 298 K): δ 152.38, 152.06, 151.49, 151.45, 151.30, 149.38, 141.37, 140.81, 138.62, 123.17, 119.61, 119.43, 119.24, 119.15, 90.12, 88.95, 88.14, 81.66, 79.34. HR-MS (ESI+) *m/z* calculated for $C_{21}H_{11}N_3^+$ $[M+H]^+$ = 306.1031, found 306.1028, Δ = 0.38 ppm.

Compound S7

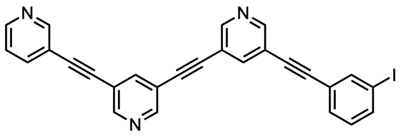

Compound **S6** (1.0 g, 3.27 mmol, 1.00 equiv.), $Pd(PPh_3)_4$ (0.189 g, 0.163 mmol, 0.05 equiv.), CuI (31 mg, 0.163 mmol, 0.05 equiv.) in dry degassed THF (25 mL) at 0 °C was added 1,3-diiodobenzene (1.18 mL, 3.60 mmol, 1.1 equiv.) and Et_3N (1.4 mL, 9.82 mmol, 3.00 equiv.). The resulting mixture was then allowed to stir overnight at 60 °C and monitored by thin-layer chromatography. After completion, the reaction mixture was filtered through celite. The resulting organic phase was diluted in dichloromethane (50 mL) and washed with a saturated solution of NH_4Cl. The combined organic layers were washed with brine (100 mL), dried (Na_2SO_4), filtered and concentrated. The residue was purified by flash chromatography (hexane/ethyl acetate = 20/80) to give product **S7** (1.2 g, 72 %) as a white solid. Melting point: 186-188 °C. ^1H NMR (500 MHz, $CDCl_3$, 298 K): δ 8.82 (br d, *J* = 2.0, 1H), 8.76-8.73 (m, 4H), 8.63 (dd, *J* = 2.0, 2.0 Hz, 1H), 8.0 (br t, *J* = 2.0 Hz, 1H), 7.97 (br t, *J* = 2.0 Hz, 1H), 7.93 (br t, *J* = 2.0 Hz, 1H), 7.87-7.85 (m, 1H), 7.75-7.73 (m, 1H), 7.54-7.52 (m, 1H), 7.35(dd, *J* = 5.0, 5.0 Hz, 1H), 7.14 (t, *J* = 8.0 Hz, 1H). ^{13}C NMR (126 MHz, $CDCl_3$, 298 K): δ 152.37, 151.49, 151.31, 151.10, 149.37, 140.82, 140.75, 140.30, 138.64, 138.11, 130.86, 130.02, 124.21, 123.19, 119.88, 119.63, 119.44, 119.30, 119.20, 93.78, 91.80, 90.12, 89.12, 88.93, 88.16, 86.16. HR-MS (ESI+) *m/z* calculated for $C_{27}H_{14}N_3I^+$ $[M+H]^+$ = 508.0310, found 508.0305, Δ = -0.46 ppm.

Compound S8

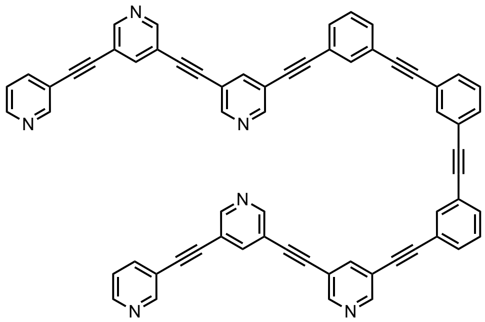

Compound **S7** (1.80 g, 3.56 mmol, 2.00 equiv.), $Pd(PPh_3)_4$ (0.205 g, 0.178 mmol, 0.1 equiv.), CuI (33 mg, 0.178 mmol, 0.1 equiv.) in dry degassed tetrahydrofuran (25 mL) at 0 °C was added 1,3-diethynylbenzene (0.237 mL, 1.78 mmol, 1.0 equiv.) and Et_3N (1.5 mL, 10.68 mmol, 6.00 equiv.). The resulting mixture was then allowed to stir overnight at 60 °C and monitored by thin-layer chromatography. After completion, the reaction mixture concentrated by rotary evaporation. The crude product was diluted in dichloromethane (50 mL) and washed with a saturated solution of NH_4Cl. The combined organic layers were washed with brine (100 mL), dried (Na_2SO_4), filtered and concentrated. The residue was purified by flash chromatography (dichloromethane/methanol = 95/5) to give product **S8** (568 g, 34 %) as a pale-yellow solid. Melting point: >400 °C (decomposition). ^1H NMR (500 MHz, $CDCl_3$, 298 K): δ 8.82 (br t, *J* = 3.0, 2H), 8.77-8.73 (m, 8H), 8.63 (br d, *J* = 5.0 Hz, 2H), 8.0 (br t, *J* = 6.0 Hz, 4H), 7.86 (br t, *J* = 6.0 Hz, 2H), 7.76 (br t, *J* = 3.0 Hz, 2H), 7.58-7.53 (m, 5H), 7.43-7.39 (m, 3H), 7.37-7.33 (m, 4H). ^{13}C NMR (126 MHz, $CDCl_3$, 298 K): δ 152.37, 151.54, 151.45, 151.31, 150.99, 149.35, 140.78, 138.64, 138.63, 135.41, 134.75, 133.17, 132.43, 132.16, 131.62, 128.70, 123.48, 123.18, 122.58, 120.07, 119.60, 119.44, 119.31, 119.16, 90.11, 89.19, 88.89, 88.18. HR-MS (ESI+) *m/z* calculated for $C_{64}H_{33}N_6^+$ $[M+H]^+$ = 885.2762, found 885.2732, Δ = 3.39 ppm.

Strand 1

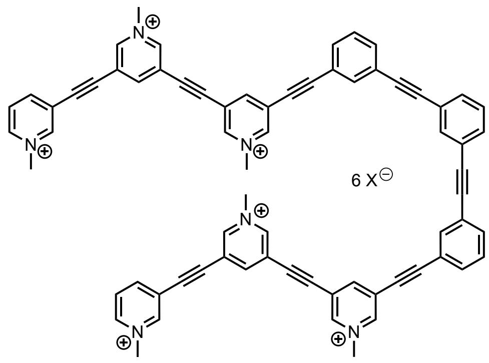

Methyl triflate (120 μL, 1.1 mmol) was added to a solution of **S7** (40 mg, 0.05 mmol) in dry N,N-dimethylformamide (2 mL) under argon atmosphere. The mixture was stirred 24 h at 60 °C, and reaction progress was monitored by LC–MS. Upon completion, water (10 mL) was added, and the mixture was lyophilized to obtain a crude solid. The residue was redissolved in water (5 mL), cooled to 0 °C, and centrifuged. The resulting precipitate was collected and washed with dichloromethane (**1·OTf**, 75 mg, 89% yield).

Ion exchange was performed on preparative HPLC to afford salt of **1·CF_3CO_2** as a beige solid (50 mg, 75% yield). Melting point: >400 °C (decomposition). ^{1}H NMR (500 MHz, CD_3CN, 298 K): δ 9.08 (br t, *J* = 2.0, 2H), 9.04 (br t, *J* = 2.0, 2H), 9.01-8.99 (m, 4H), 8.93 (br t, *J* = 1.0 Hz, 2H), 8.83 (br t, *J* = 2.0 Hz, 2H), 8.74 (br t, *J* = 2.0 Hz, 2H), 8.72-8.70 (m, 2H), 8.65 (br t, *J* = 2.0 Hz, 1H), 8.63 (br t, *J* = 2.0 Hz, 1H) 8.12-8.09 (m, 2H), 7.83 (t, *J* = 2.0 Hz, 2H), 7.77 (t, *J* = 1.0 Hz, 1H), 7.73-7.68 (m, 4H), 7.63 (dd, *J* = 1.0, 2.0 Hz, 2H), 7.57-7.48 (m, 3H), 4.40 (s, 6H), 4.37 (s, 6H), 4.36 (s, 6H), ^{13}C NMR (126 MHz, CD_3CN, 298 K): δ 160.6, 160.3, 150.2, 149.6, 149.1, 149.0, 148.8, 148.5, 147.8, 147.0, 135.5, 135.1, 134.5, 133.2, 132.9, 130.5, 130.3, 129.4, 124.5, 124.0, 123.6, 123.4, 123.1, 122.9, 122.0, 120.7, 116.0, 90.2, 89.5, 89.3, 88.8, 82.4, 50.2, 50.0, 49.7. HR-MS (ESI+) *m/z* calculated for $C_{80}H_{50}F_{15}N_6O_{10}^+$ [M+5CF_3CO_2]$^+$ = 1539.3344, found 1539.3328, Δ = -1.04 ppm.

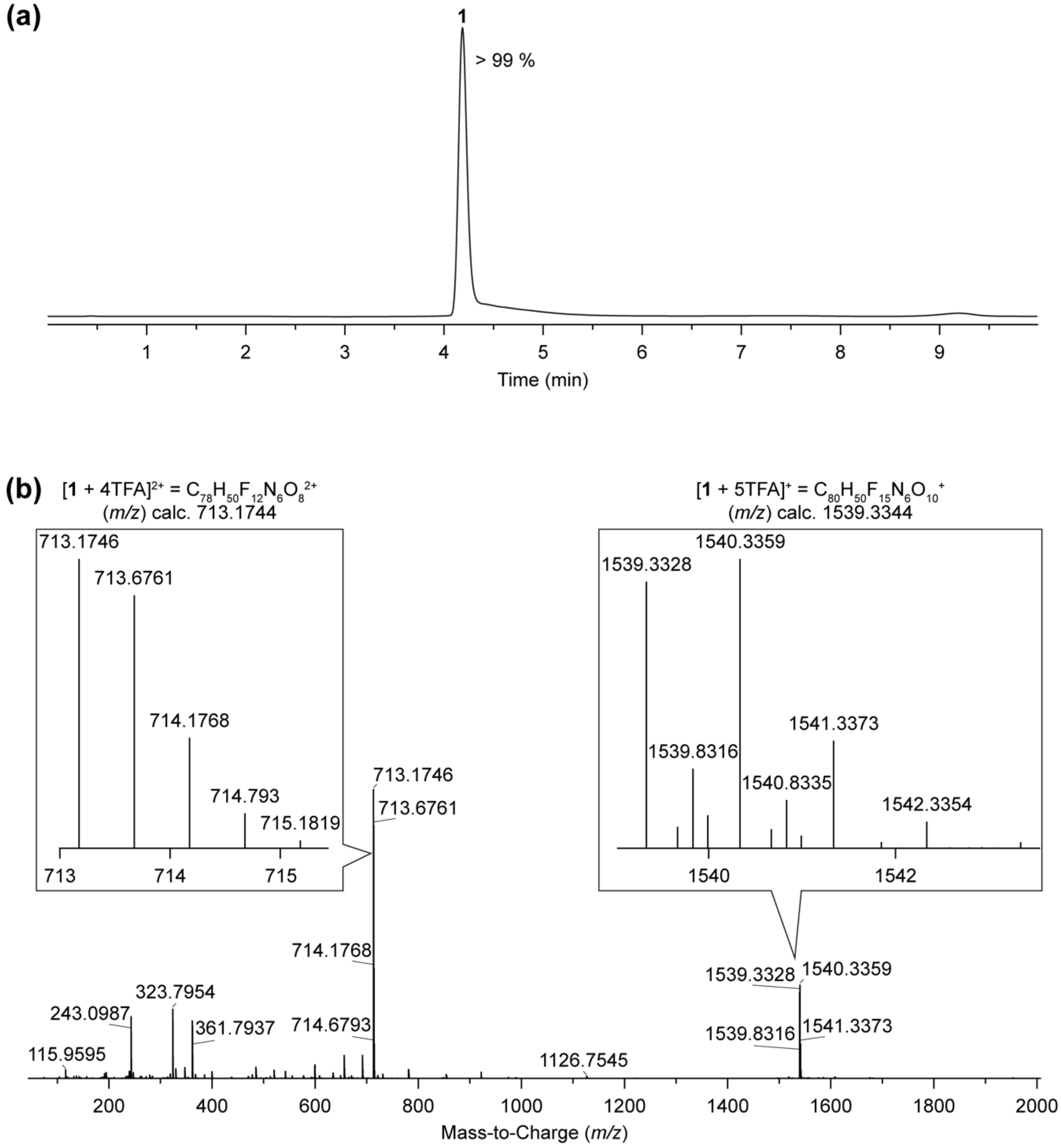

Figure S1 | UHPLC-MS characterization of strand 1. Tandem **(a)** Reverse-phase UHPLC chromatogram and **(b)** MS spectrum of strand **1**. Absorbance was recorded at 254 nm.

2.2. Synthesis of strand 2

Strand **2** was synthesized according to the following procedure:

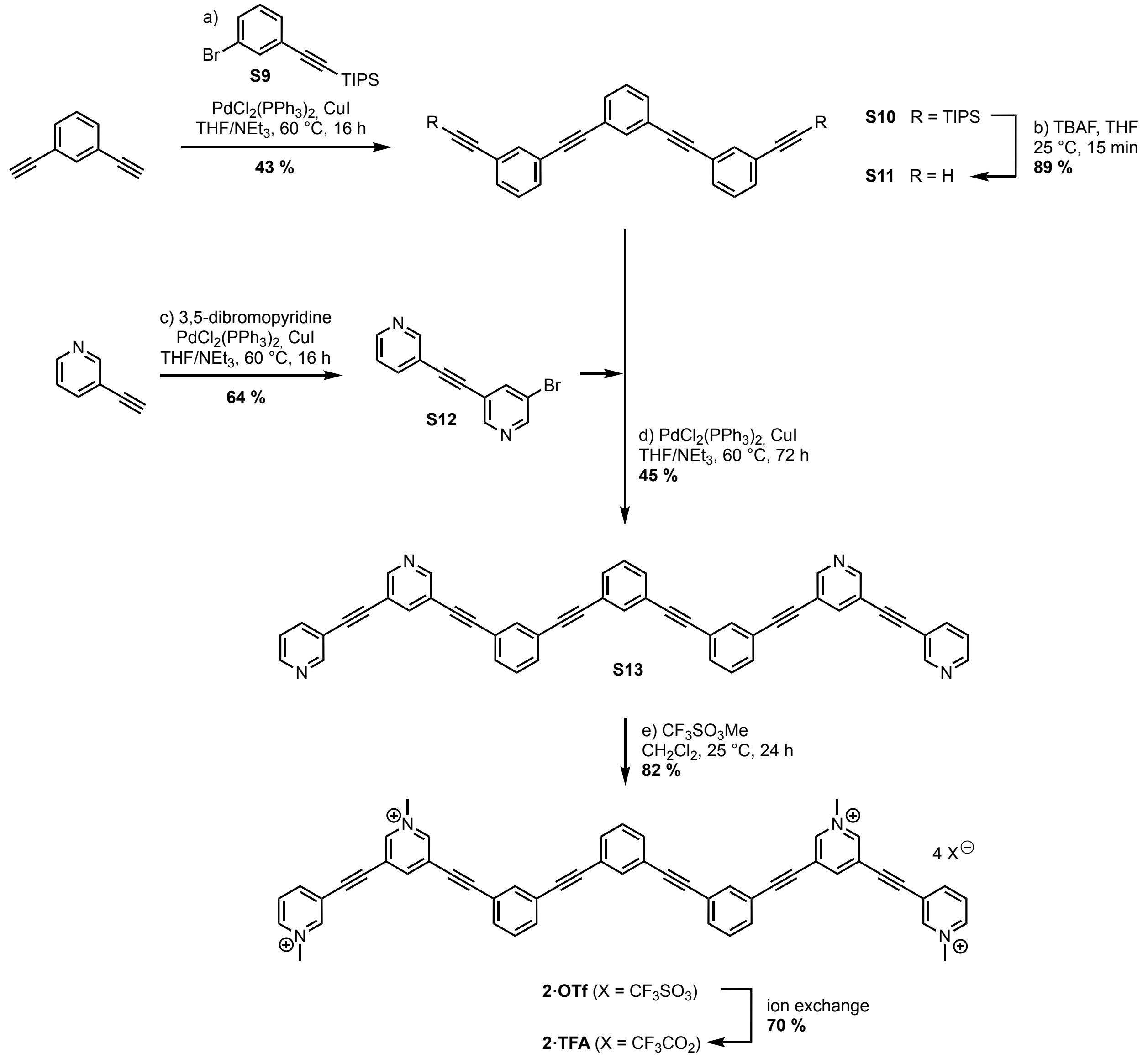

Compound S9

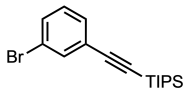

The synthesis of **S9** was adapted from a published procedure.[2] 3-bromoiodobenzene (735 µL, 5.7 mmol), (triisopropylsilyl)acetylene (1.4 mL, 6.4 mmol), $PdCl_2(PPh_3)_2$ (206 mg, 0.3 mmol), and CuI (30 mg, 0.2 mmol) were dissolved in dry triethylamine (18 mL) at 0 °C under nitrogen atmosphere. The reaction mixture was stirred 16 h at 25 °C, concentrated by rotary evaporation, and the resulting residue was purified by silica gel column chromatography (100% cyclohexane) to yield **S9** (1.9 g, 97 %) as a colorless oil. Spectral data closely match those previously reported in the literature.[2] ^{1}H NMR (400 MHz, $CDCl_3$ + 0.03 % tetramethylsilane, 293 K): δ 7,61 (dd, J = 2.6, 0.9 Hz, 1H), 7.42 (br s, 2H), 7.19 (t, J = 7.9 Hz, 1H), 1.12 (s, 21H). ^{13}C NMR (100 MHz, $CDCl_3$ + 0.03 % tetramethylsilane, 293 K): δ 135.0, 131.9, 130.9, 130.2, 125.8, 122.3, 105.6, 92.8, 18.8, 11.6.

Compound S10

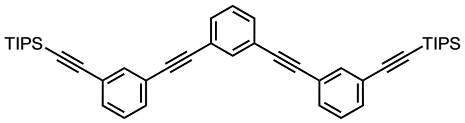

The synthesis of **S10** was adapted from a published procedure.[3] 1,3-diethynylbenzene (263 µL, 2.0 mmol), **S9** (1.5 g, 4.4 mmol), $PdCl_2(PPh_3)_2$ (139 mg, 0.2 mmol), and CuI (20 mg, 0.1 mmol) were dissolved in dry triethylamine (10 mL) and tetrahydrofuran (10 mL) at 0 °C under nitrogen atmosphere. The reaction mixture was stirred 16 h at 60 °C, concentrated by rotary evaporation, and the resulting residue was purified by silica gel column chromatography (gradient hexane/dichloromethane = 100/0 to 90/10) to yield **S10** (551 mg, 43 %) as a colorless oil. Spectral data closely match those previously reported in the literature.[3] ^{1}H NMR (400 MHz, $CDCl_3$ + 0.03 % tetramethylsilane, 293 K): δ 7.71 (t, J = 1.4 Hz, 1H), 7.48 (m, 6H), 7.33 (t, J = 7.7 Hz, 1H), 7.31 (t, J = 7.7 Hz, 2H), 1.26 (m, 6H), 1.14 (s, 36H). ^{13}C NMR (100 MHz, $CDCl_3$ + 0.03 % tetramethylsilane, 293 K): δ 135.2, 134.6, 132.4, 131.4, 130.1, 128.4, 123.6, 123.5, 89.7, 89.0, 82.4, 78.3, 18.7, 11.3.

Compound S11

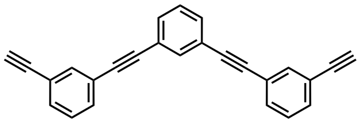

The synthesis of **S11** was adapted from a published procedure.[3] Tetrabutylammonium fluoride (1 M in tetrahydrofuran, 4.6 mL) was added to a solution of **S10** (829 mg, 1.3 mmol) in tetrahydrofuran (150 mL). The reaction mixture was stirred for 15 min at 25 °C and concentrated by rotary evaporation. The crude product was first washed with plenty of CH_3OH. The residue was then redissolved in dichloromethane, washed with water, dried over Na_2SO_4, filtered and concentrated by rotary evaporation to yield **S11** (376 mg, 89 %) as a white solid. Spectral data closely match those previously reported in the literature.[3] ^{1}H NMR (400 MHz, $CDCl_3$ + 0.03 % tetramethylsilane, 293 K): δ 7.72 (t, *J* = 1.4 Hz, 1H), 7.69 (t, *J* = 1.4 Hz, 2H), 7.50 (m, 6H), 7.34 (t, *J* = 7.7 Hz, 1H), 7.31 (t, *J* = 7.7 Hz, 2H), 3.12 (s, 2H). ^{13}C NMR (100 MHz, $CDCl_3$ + 0.03 % tetramethylsilane, 293 K): δ 135.2, 134.7, 132.0, 131.9, 131.6, 128.5, 123.4, 122.6, 89.1, 89.0, 82.7, 77.9.

Compound S12

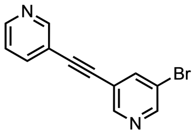

3-Ethynylpyridine (1.0 g, 9.8 mmol), 3,5-dibromopyridine (3.0 g, 12.8 mmol), $PdCl_2(PPh_3)_2$ (341 mg, 0.5 mmol) and CuI (53 mg, 0.3 mmol) were dissolved in dry triethylamine (35 mL) and tetrahydrofuran (43 mL) at 0 °C under nitrogen atmosphere. The reaction mixture was stirred 16 h at 60 °C, concentrated by rotary evaporation, and the resulting residue was purified by silica gel column chromatography (gradient hexane/ethyl acetate = 100/0 to 50/50) to yield **S12** (1.6 g, 64 %) as a white solid. Melting point: 91-92 °C. ^{1}H NMR (400 MHz, $CDCl_3$ + 0.03 % tetramethylsilane, 293 K): δ 8.78 (br d, *J* = 1.5 Hz, 1H), 8.68 (d, *J* = 2.0 Hz, 1H), 8.65 (d, *J* = 2.0 Hz, 1H), 8.61 (br dd, *J* = 4.9, 1.5 Hz, 1H), 7.99 (t, *J* = 2.0 Hz, 1H), 7.83 (dt, *J* = 7.9, 2.0 Hz, 1H), 7.33 (ddd, *J* = 7.9, 4.9, 0.7 Hz, 1H). ^{13}C NMR (100 MHz, $CDCl_3$ + 0.03 % tetramethylsilane, 293 K): δ 152.5, 150.4, 150.3, 149.5, 140.9, 138.8, 123.3, 121.2, 120.3, 119.4, 90.5, 87.7. HR-MS (ESI+) *m/z* calculated for $C_{12}H_7BrN_2^+$ $[M]^+$ = 257.9793, found 257.9798, Δ = -2.02 ppm.

Compound S13

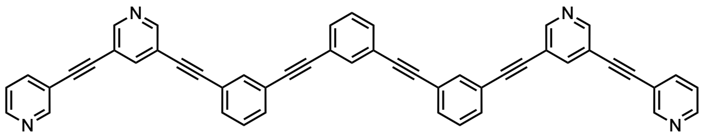

Compounds **S11** (395 mg, 1.2 mmol), **S12** (624 mg, 2.4 mmol), $PdCl_2(PPh_3)_2$ (86 mg, 0.1 mmol) and CuI (14 mg, 0.07 mmol) were dissolved in dry triethylamine (10 mL) and tetrahydrofuran (10 mL) at 0 °C under nitrogen atmosphere. The reaction mixture was stirred for 72 h at 60 °C and concentrated by rotary evaporation. The solid residue was dissolved in dichloromethane, washed with a saturated solution of Na_2CO_3, dried over Na_2SO_4, filtered, and concentrated by rotary evaporation. The resulting solid was washed several times with petroleum ether and methanol to yield **S13** (372 mg, 45 %) as a beige solid. Melting point: >400 °C (decomposition). ^1H NMR (400 MHz, $CDCl_3$ + 0.03 % tetramethylsilane, 293 K): δ 8.79 (br d, *J* = 1.5 Hz, 2H), 8.71 (d, *J* = 2.0 Hz, 2H), 8.70 (d, *J* = 2.0 Hz, 2H), 8.60 (br dd, *J* = 5.0, 1.5 Hz, 2H), 7.96 (t, *J* = 2.0 Hz, 2H), 7.83 (dt, *J* = 8.0, 2.0 Hz, 2H), 7.73 (t, *J* = 1.5 Hz, 1H), 7.72 (t, *J* = 1.5 Hz, 2H), 7.55-7.49 (m, 6H), 7.38 (t, *J* = 8.0 Hz, 2H), 7.34 (t, *J* = 8.0 Hz, 1H), 7.32 (ddd, *J* = 8.0, 5.0, 1.0 Hz, 2H). ^{13}C NMR (100 MHz, $CDCl_3$ + 0.03 % tetramethylsilane, 293 K): δ 152.5, 151.4, 151.1, 149.4, 140.8, 138.7, 134.9, 134.8, 132.2, 131.7, 128.8, 128.7, 123.7, 123.4, 123.3, 122.7, 120.1, 119.6, 92.7, 89.9, 89.4, 89.0, 88.5, 85.8. HR-MS (ESI+) *m/z* calculated for $C_{50}H_{27}N_4^+$ $[M+H]^+$ = 683.2231, found 683.2238, Δ = -1,02 ppm.

Strand 2

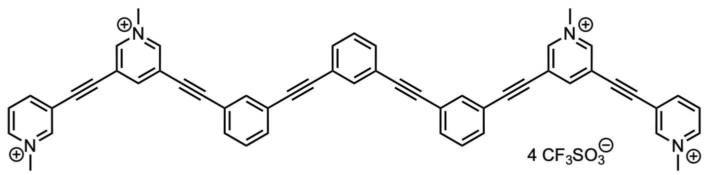

Methyl triflate (154 µL, 1.4 mmol) was added to a solution of **S13** (169 mg, 0.3 mmol) in dry dichloromethane (120 mL) under nitrogen atmosphere. The mixture was stirred 24 h at 25 °C before being concentrated by rotary evaporation. The crude product was washed with dichloromethane to yield **2·OTf** (270 mg, 82 %) as a beige solid. Melting point: >400 °C (decomposition). ^{1}H NMR (500 MHz, CD_3CN, 298 K): δ 9.01 (br s, 2H), 8.94 (br s, 2H), 8.91 (br s, 2H), 8.74 (br s, 2H), 8.71 (br d, *J* = 6.3 Hz, 2H), 8.65 (br d, *J* = 8.2 Hz, 2H), 8.99 (br t, *J* = 7.2 Hz, 2H), 7.84 (br t, *J* = 1.7 Hz, 2H), 7.77 (br t, *J* = 1.6 Hz, 1H), 7.71 (br dt, *J* = 7.8, 1.3 Hz, 2H), 7.69 (br dt, *J* = 7.9, 1.4 Hz, 2H), 7.62 (br dd, *J* = 7.8, 1.7 Hz, 2H), 7.55 (t, *J* = 7.8 Hz, 2H), 7.49 (t, *J* = 7.8 Hz, 1H), 4.37 (s, 6H), 4.36 (s, 6H). ^{13}C NMR (126 MHz, CD_3CN, 298 K): δ 149.6, 149.1, 148.6, 148.5, 147.7, 146.9, 135.6, 135.2, 134.5, 133.2, 132.9, 130.6, 130.3, 129.4, 125.4, 124.6, 124.1, 123.4, 123.1, 122.1, 98.1, 90.2, 89.8, 89.4, 88.6, 82.6, 50.0, 49.8. HR-MS (ESI+) *m/z* calculated for $C_{57}H_{38}F_9N_4O_9S_3^+$ $[M+3CF_3SO_3]^+$ = 1189.1652, found 1189.1686, Δ = -2.86 ppm.

Ion exchange was performed on preparative HPLC. Strand **2·TFA** (169 mg, 70 %) was isolated as a beige solid. HR-MS (ESI+) *m/z* calculated for $C_{60}H_{38}F_9N_4O_6^+$ $[M+3CF_3CO_2]^+$ = 1081.2643, found 1081.2623, Δ = 1.85 ppm.

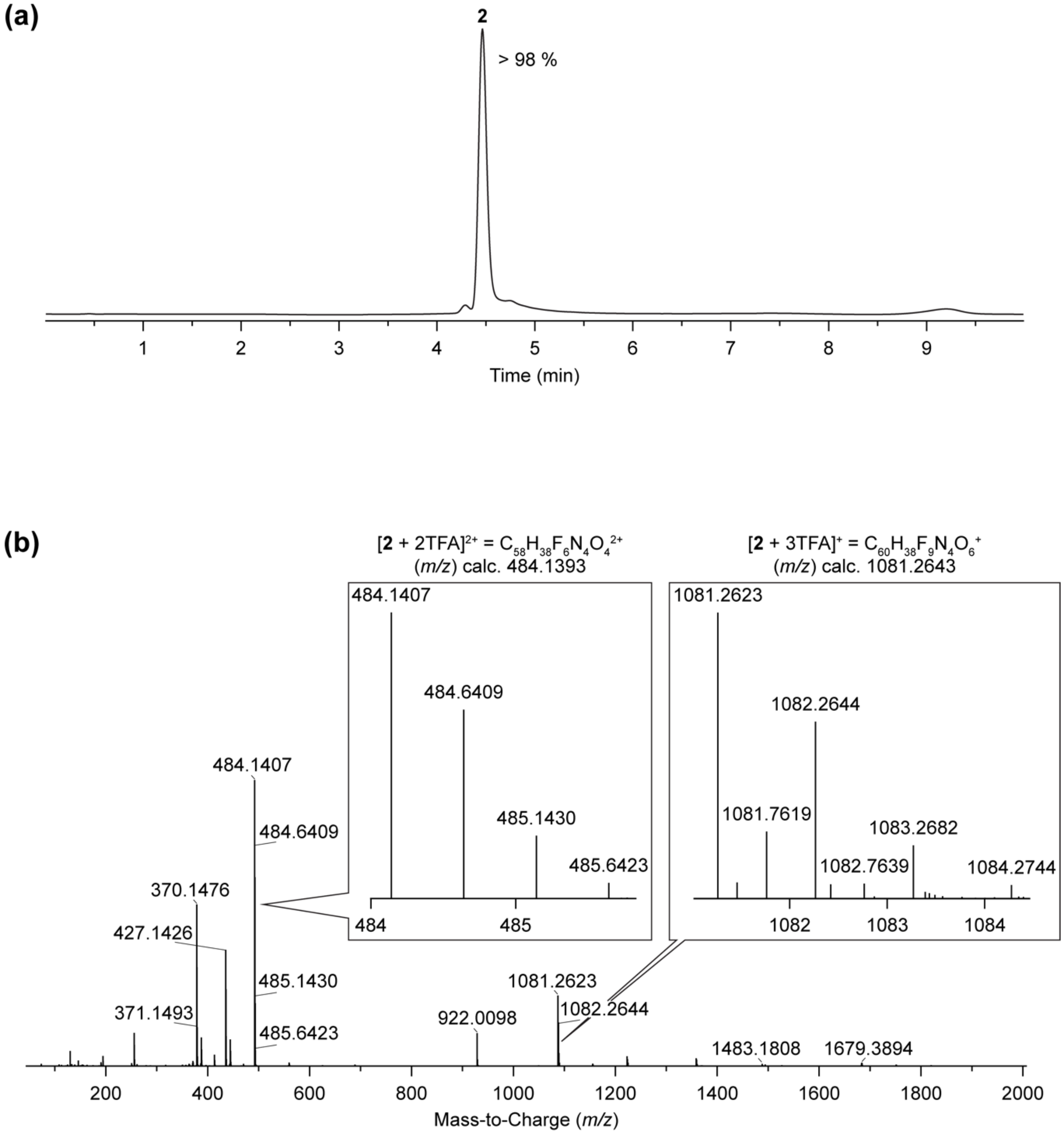

Figure S2 | UHPLC-MS characterization of strand 2. Tandem **a,** Reverse-phase UHPLC chromatogram and **b,** MS spectrum of strand **2**. Absorbance was recorded at 254 nm.

3. NMR characterization of unfolded strand 1 in CD_3CN

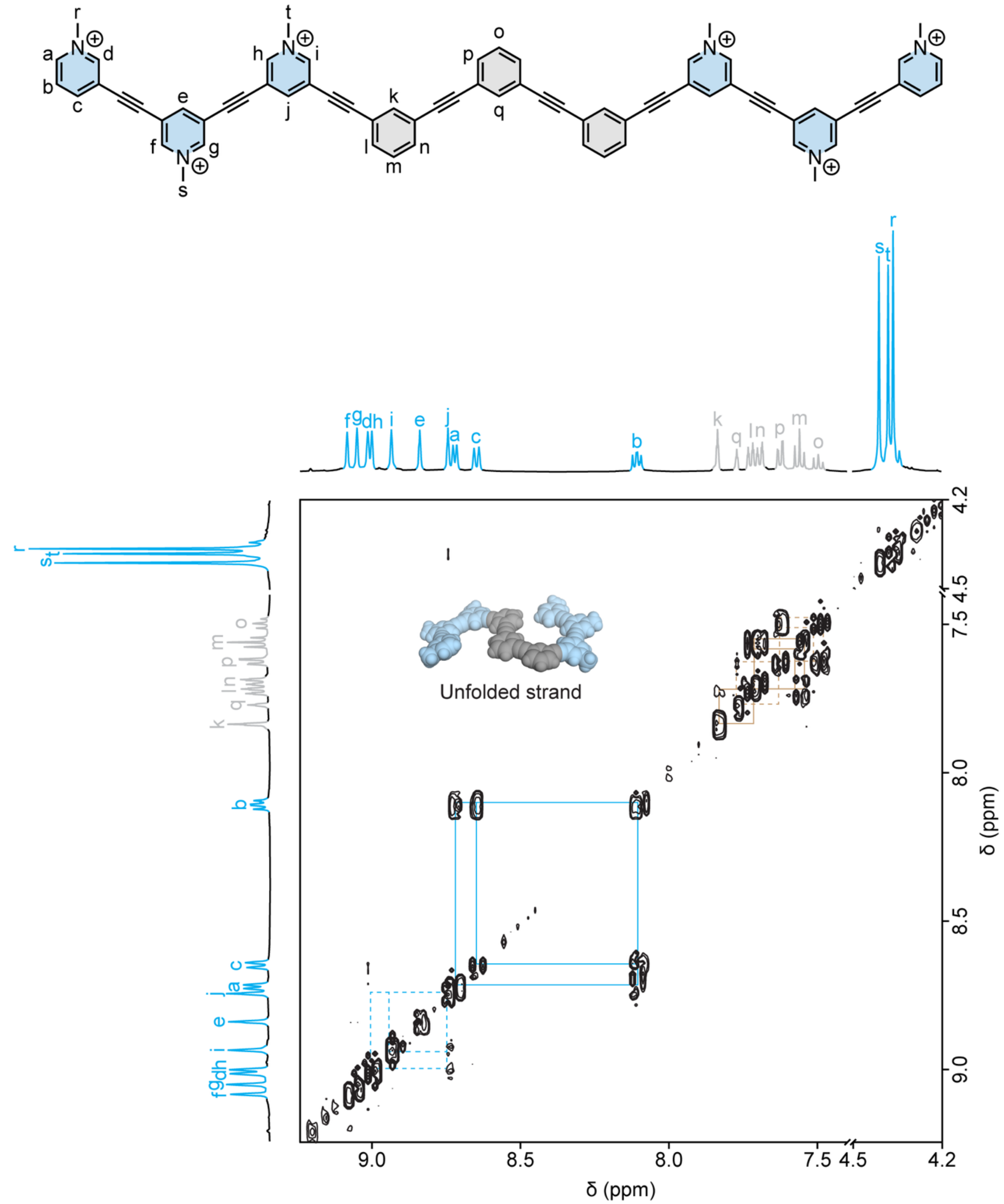

Figure S3 | Expansion of 2D ^{1}H-^{1}H COSY NMR spectrum of unfolded strand 1·TFA in CD_3CN (5 mM, 500 MHz, 298 K).

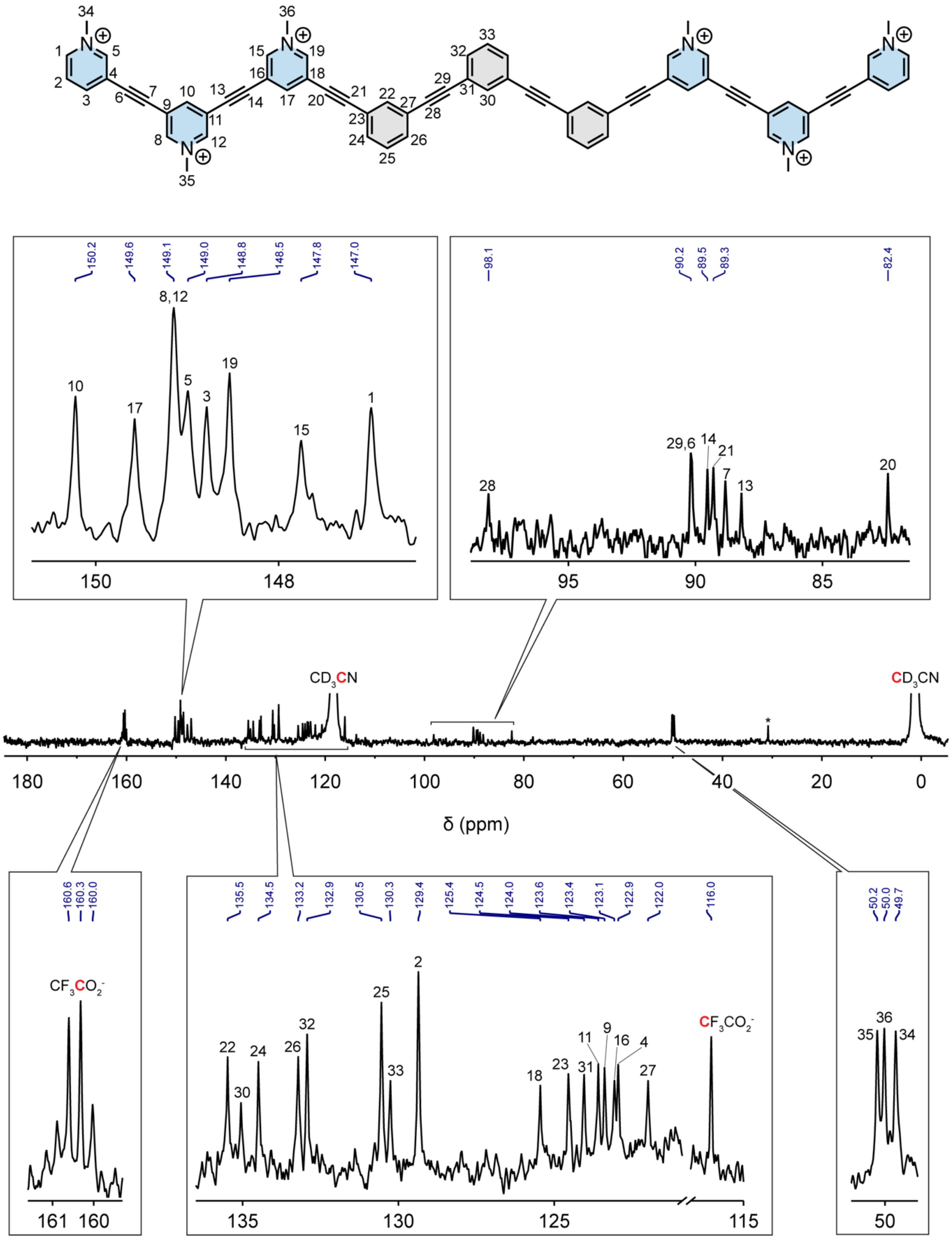

Figure S4 | ^{13}C NMR spectrum of unfolded strand 1·TFA in CD_3CN (5 mM, 126 MHz, 298 K). Assigned ^{13}C NMR chemical shifts are denoted onto the 1·TFA structure.

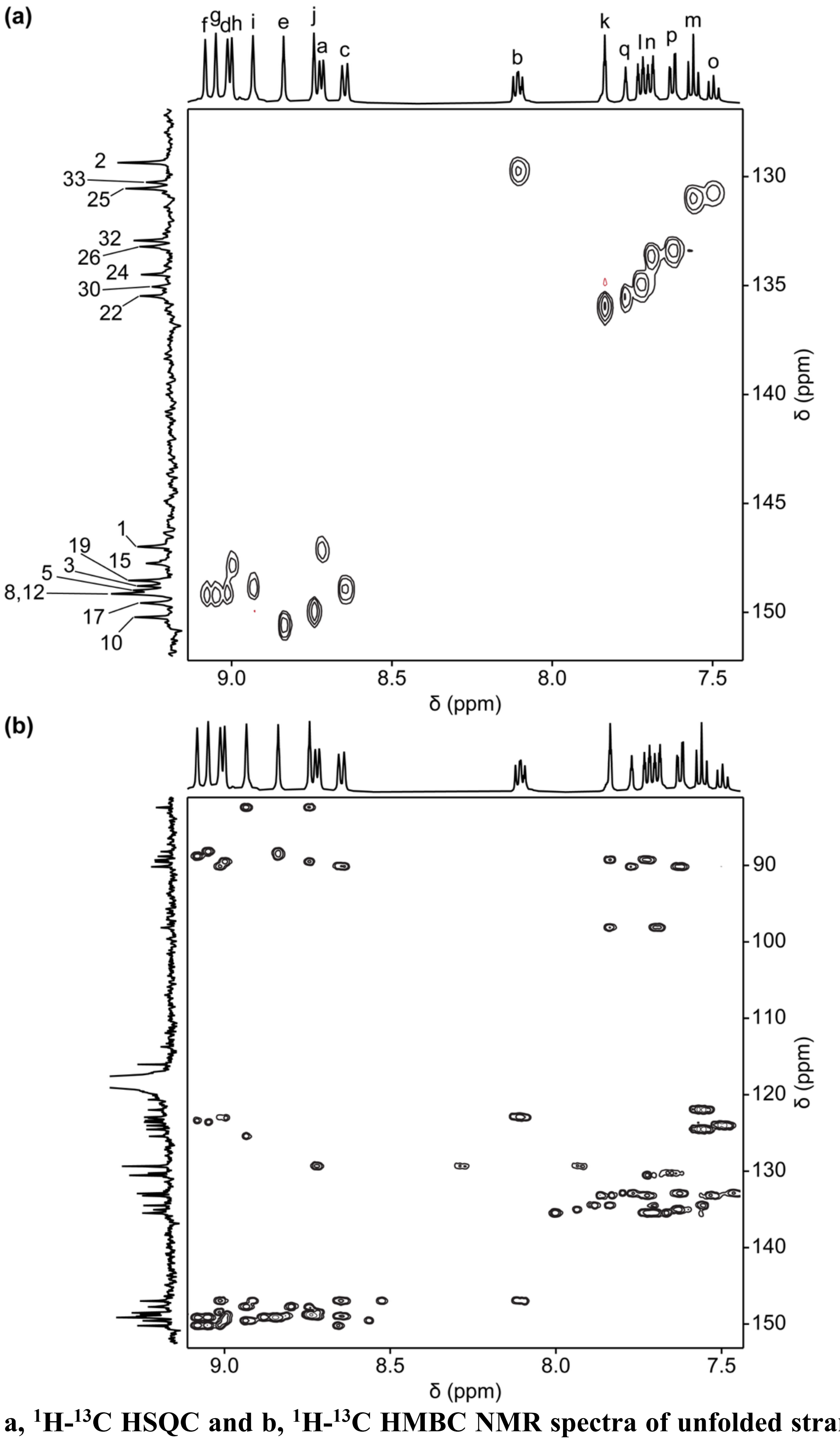

Figure S5 | a, ^{1}H-^{13}C HSQC and b, ^{1}H-^{13}C HMBC NMR spectra of unfolded strand 1·TFA in CD_3CN (5 mM, 500 MHz, 298 K).

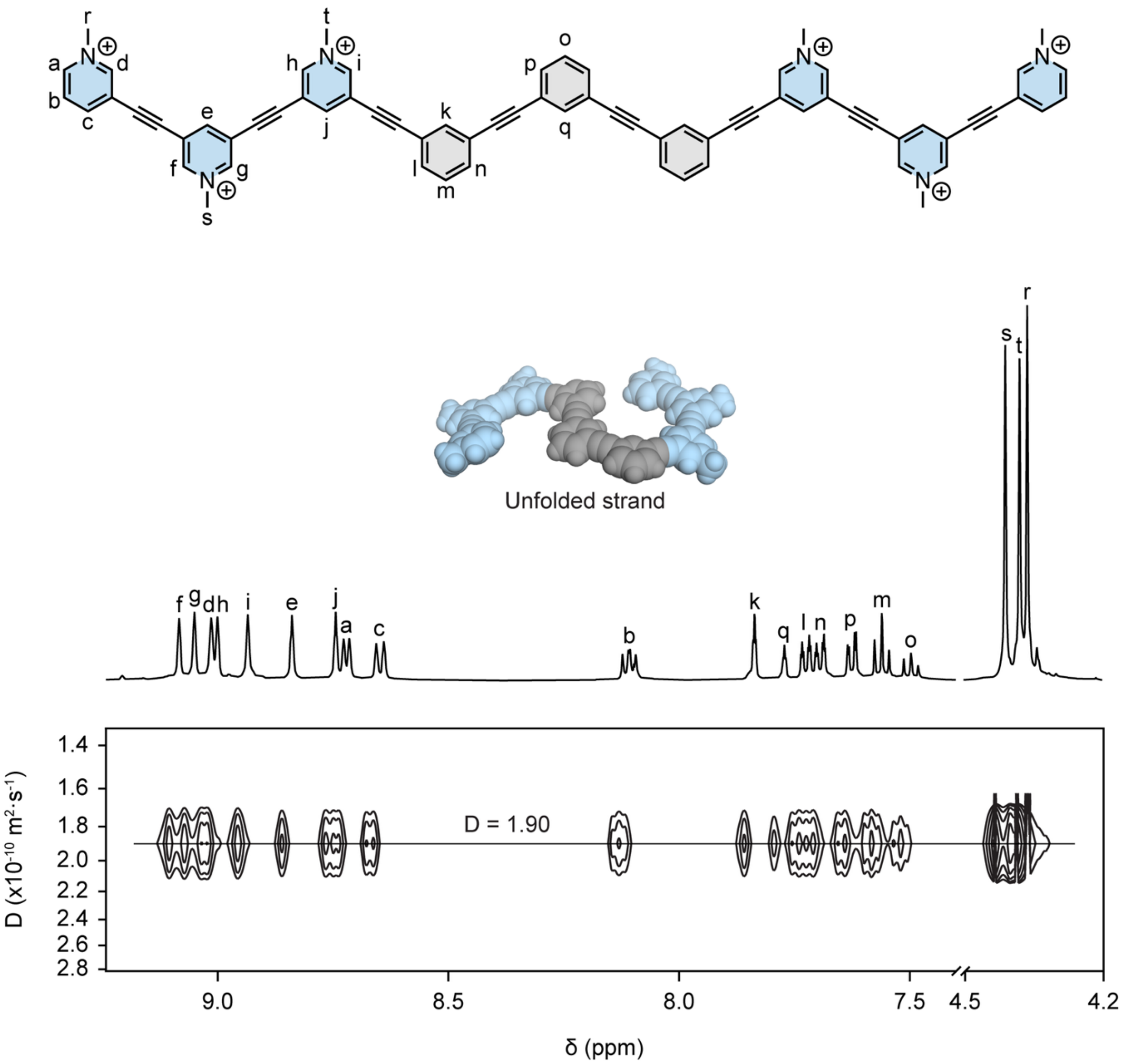

Figure S6 | Expansion of ^{1}H and DOSY NMR spectra of unfolded strand 1·TFA in CD_3CN (5 mM, 500 MHz, 298 K). Hydrodynamic radius r_H = 12.0 Å was calculated using the Stokes-Einstein equation.

4. NMR characterization of double helix $(1)_2$ in D_2O

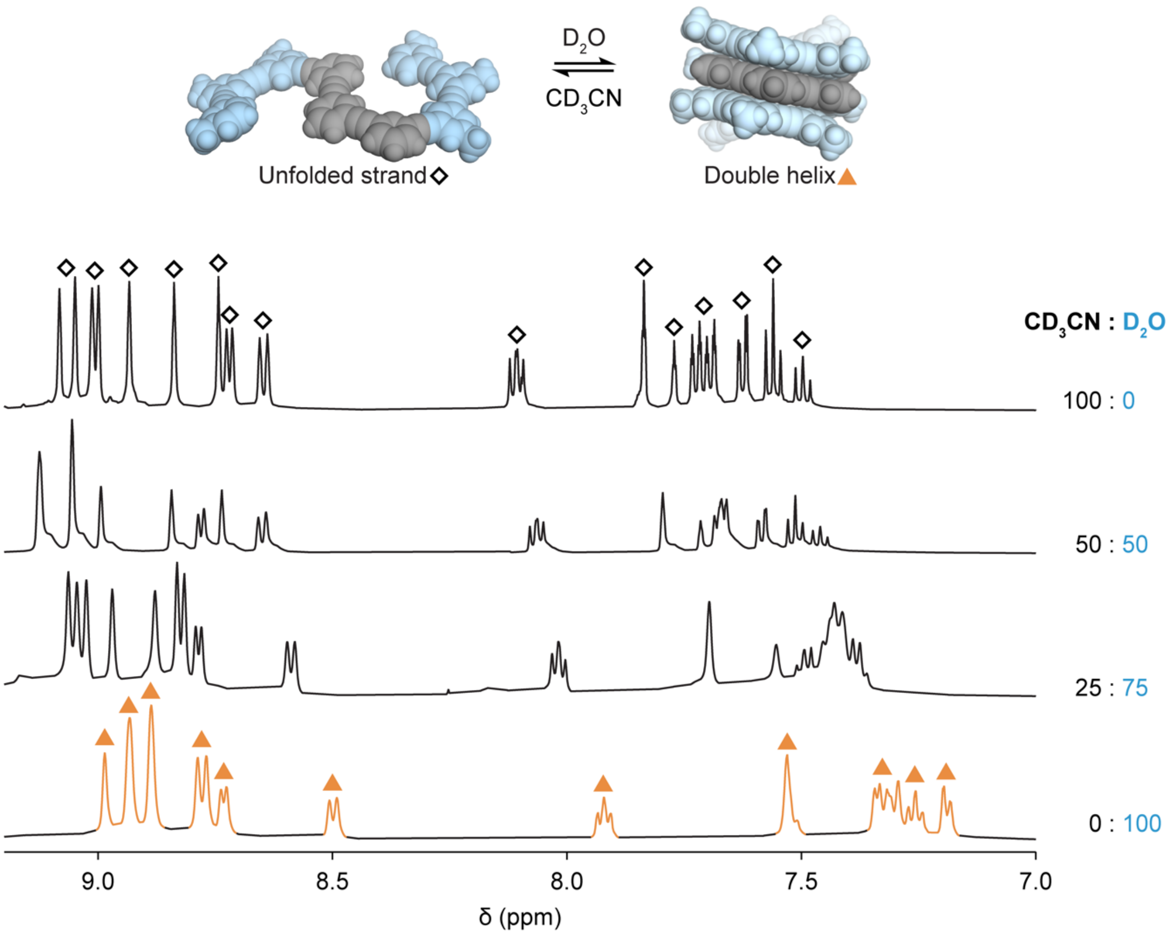

Figure S7 | Expansion of aromatic ^{1}H NMR spectral region of 1·TFA in CD_3CN / D_2O mixtures (5 mM, 500 MHz, 298 K). The signals corresponding to the unfolded strand and double helix are labelled with empty diamonds and orange triangles, respectively.

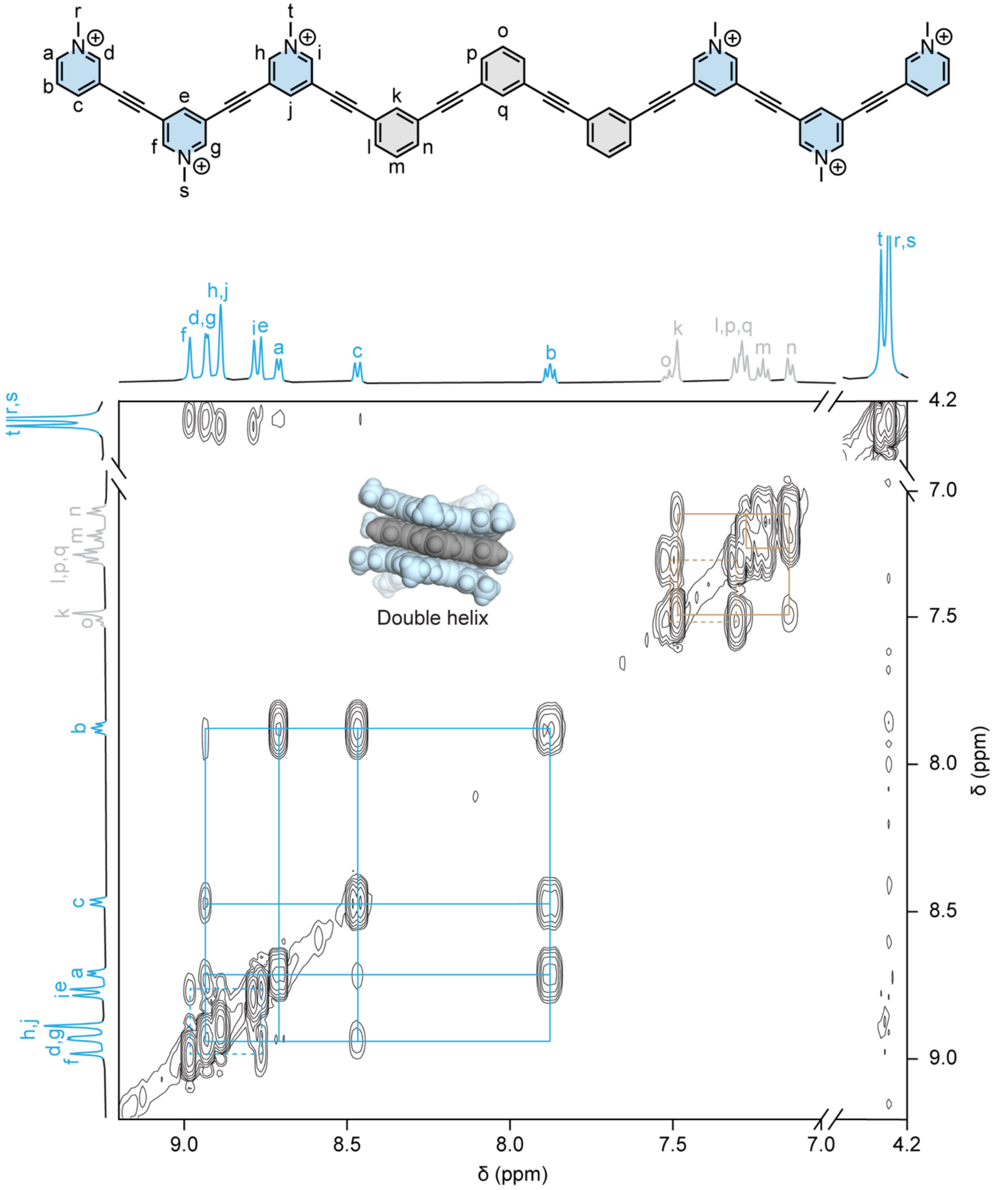

Figure S8 | Expansion of selected regions in 2D ^{1}H-^{1}H COSY NMR spectrum of double helix $(1)_2$·TFA in D_2O (10 mM, 500 MHz, 278 K).

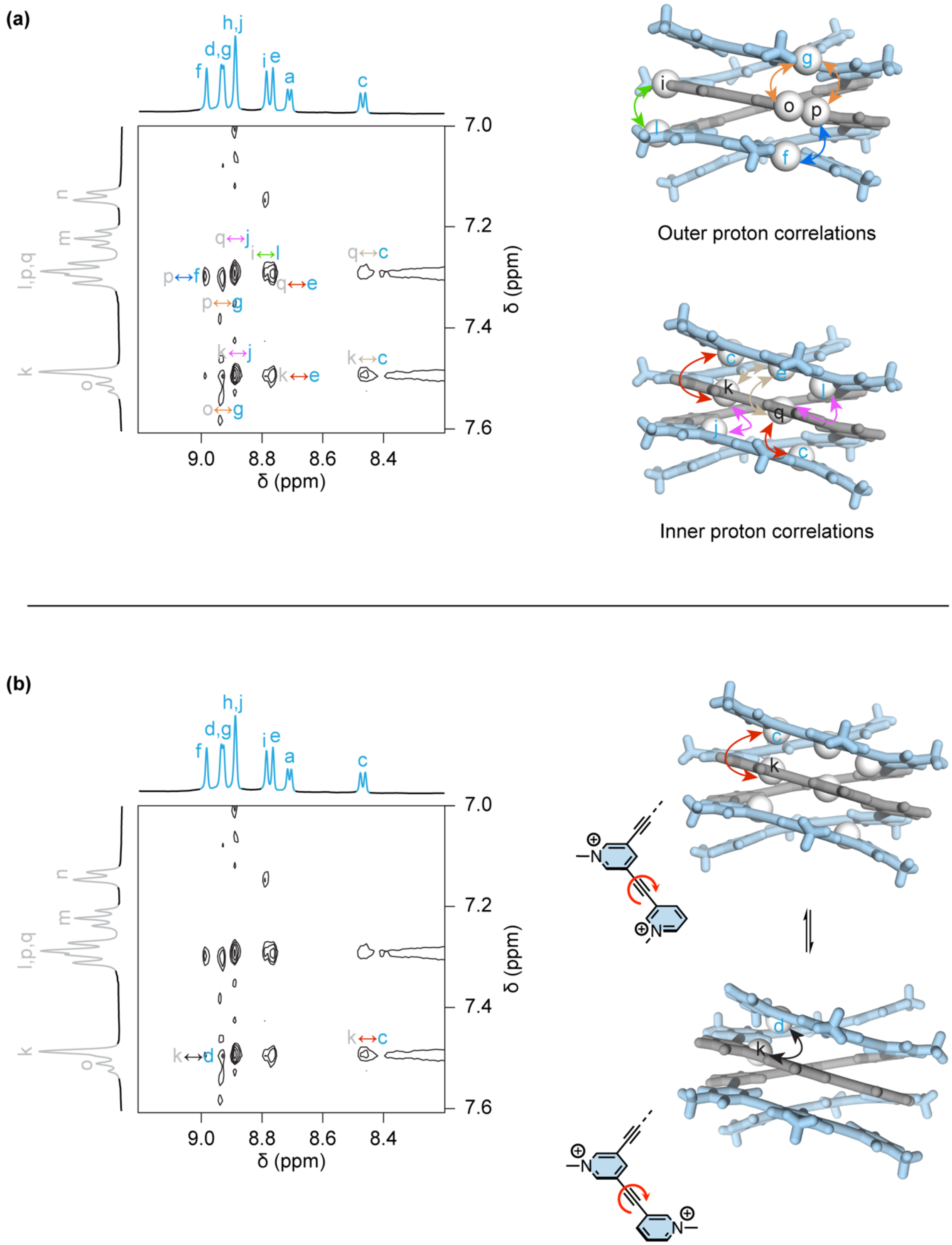

Figure S9 | Expansion of 2D ^{1}H-^{1}H ROESY NMR spectrum of double helix $(1)_2$·TFA in D_2O (10 mM, 500 MHz, 278 K, 100 ms mixing time). a, Key ROE correlations between protons of stacked pyridinium (colored in blue) and phenylene (colored in gray) residues. **b,** Rotation of the terminal pyridinium residue around the acetylene bond results in the appearance of additional ROE correlations between stacked pyridinium and phenylene residues.

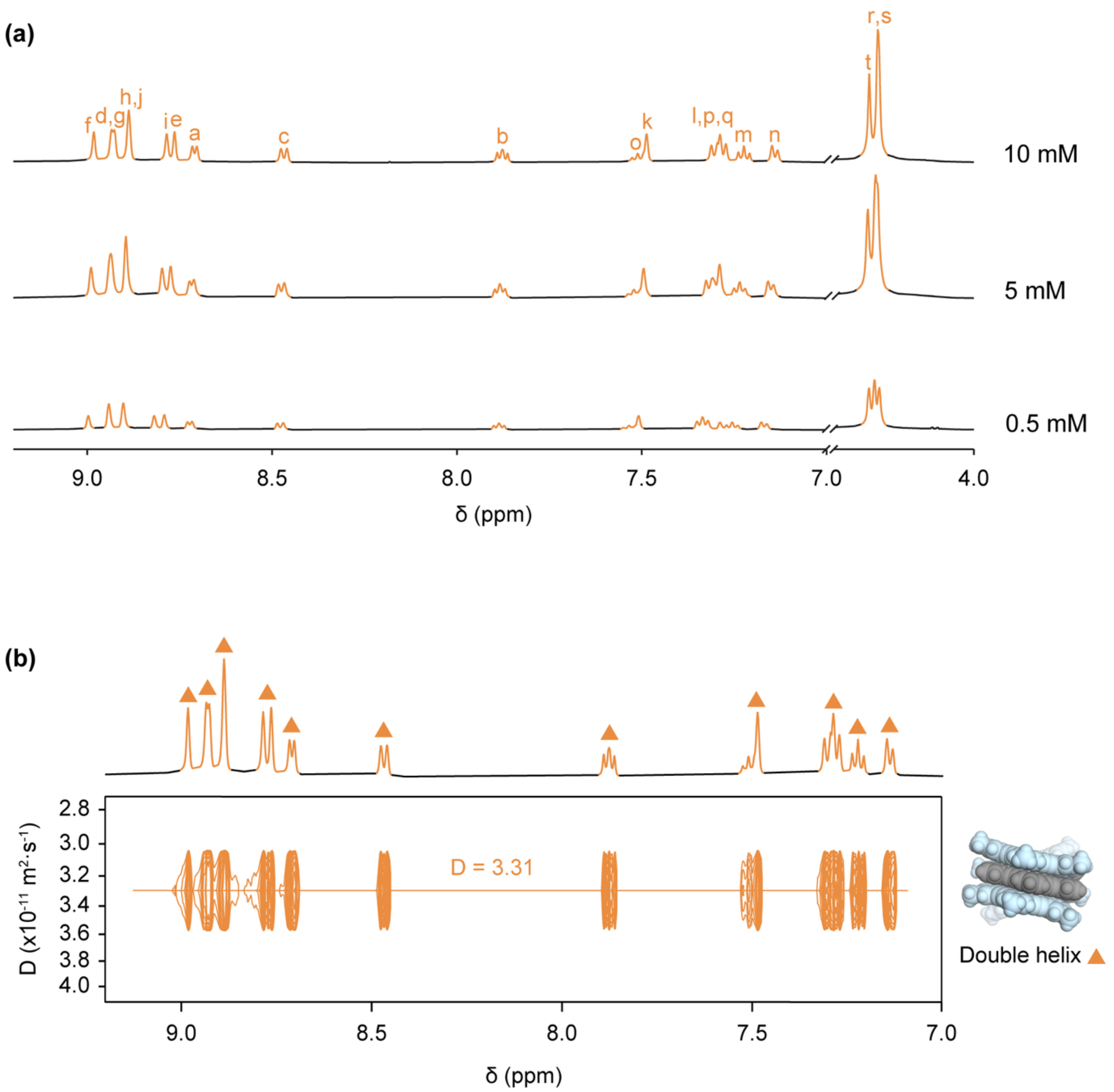

Figure S10 | Concentration-dependent ^{1}H and DOSY NMR spectra of double helix (1)$_2$·TFA. **a,** Concentration-dependent ^{1}H NMR spectra of **1·TFA** between 0.5 mM and 10 mM in D_2O (500 MHz, 278 K). **b,** ^{1}H DOSY NMR spectrum (500 MHz, D_2O, 278 K) of the double helix assembled from **1·TFA** (10 mM) in D_2O. The signals corresponding to the double helix are labelled with orange triangles. Its hydrodynamic radius r_H = 8.4 Å was calculated using the Stokes-Einstein equation.

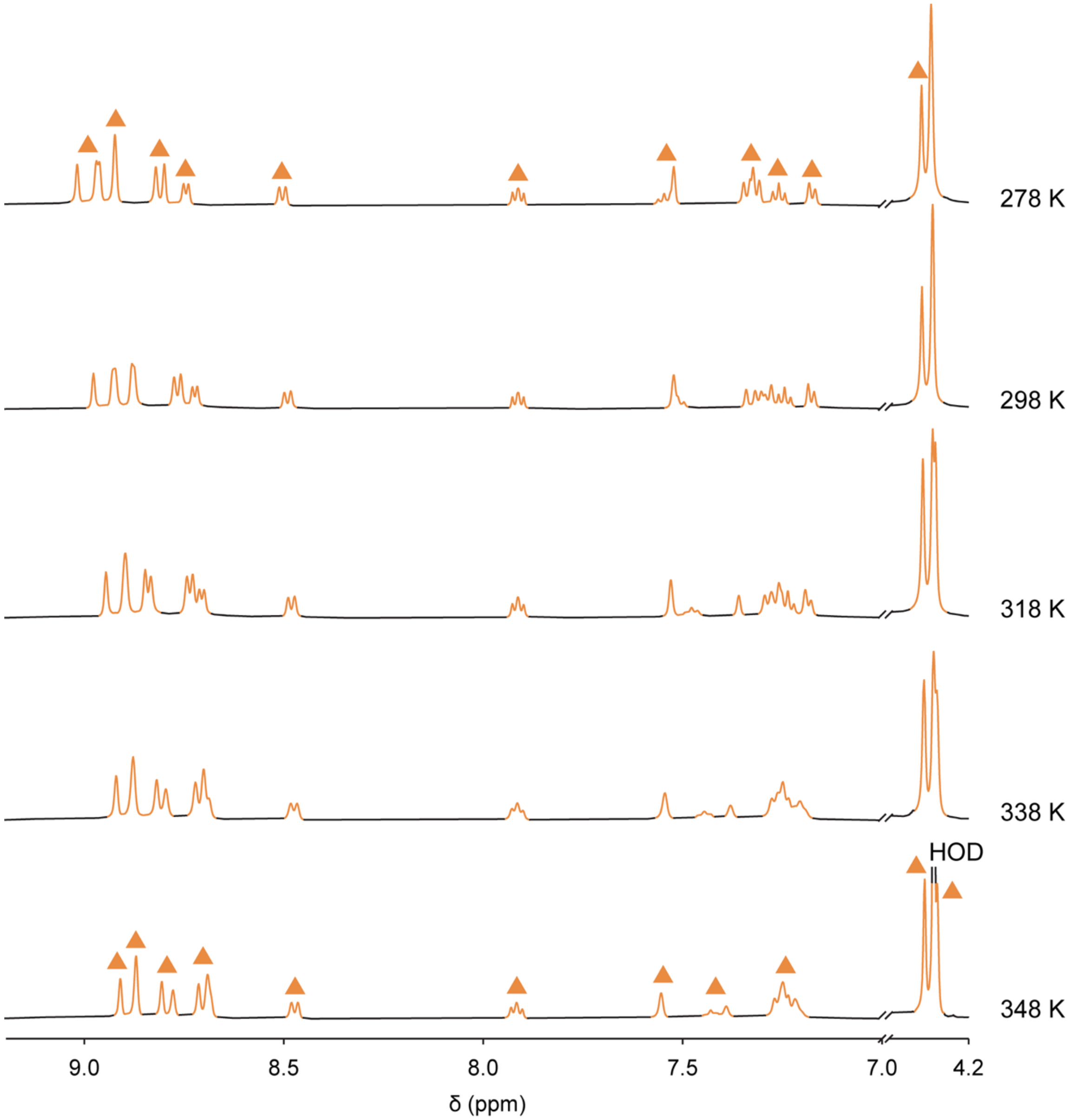

Figure S11 | Variable temperature ^{1}H NMR spectra (500 MHz) of a 10 mM solution of 1·TFA in D_2O between 278 K and 348 K. The signals corresponding to the double helix are labelled with orange triangles.

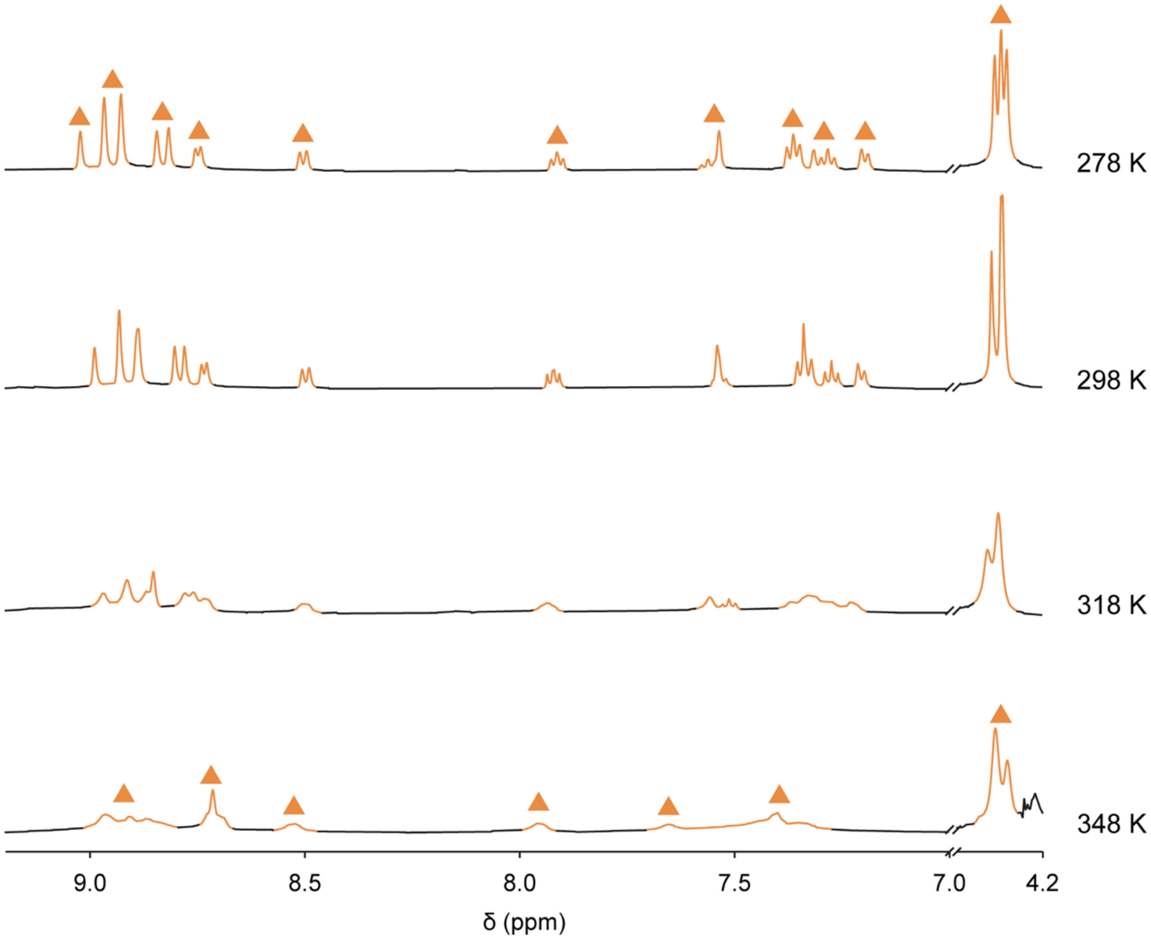

Figure S12 | Variable temperature ^{1}H NMR spectra (500 MHz) of a 0.5 mM solution of 1·TFA in D_2O between 278 K and 348 K. The signals corresponding to the double helix are labelled with orange triangles.

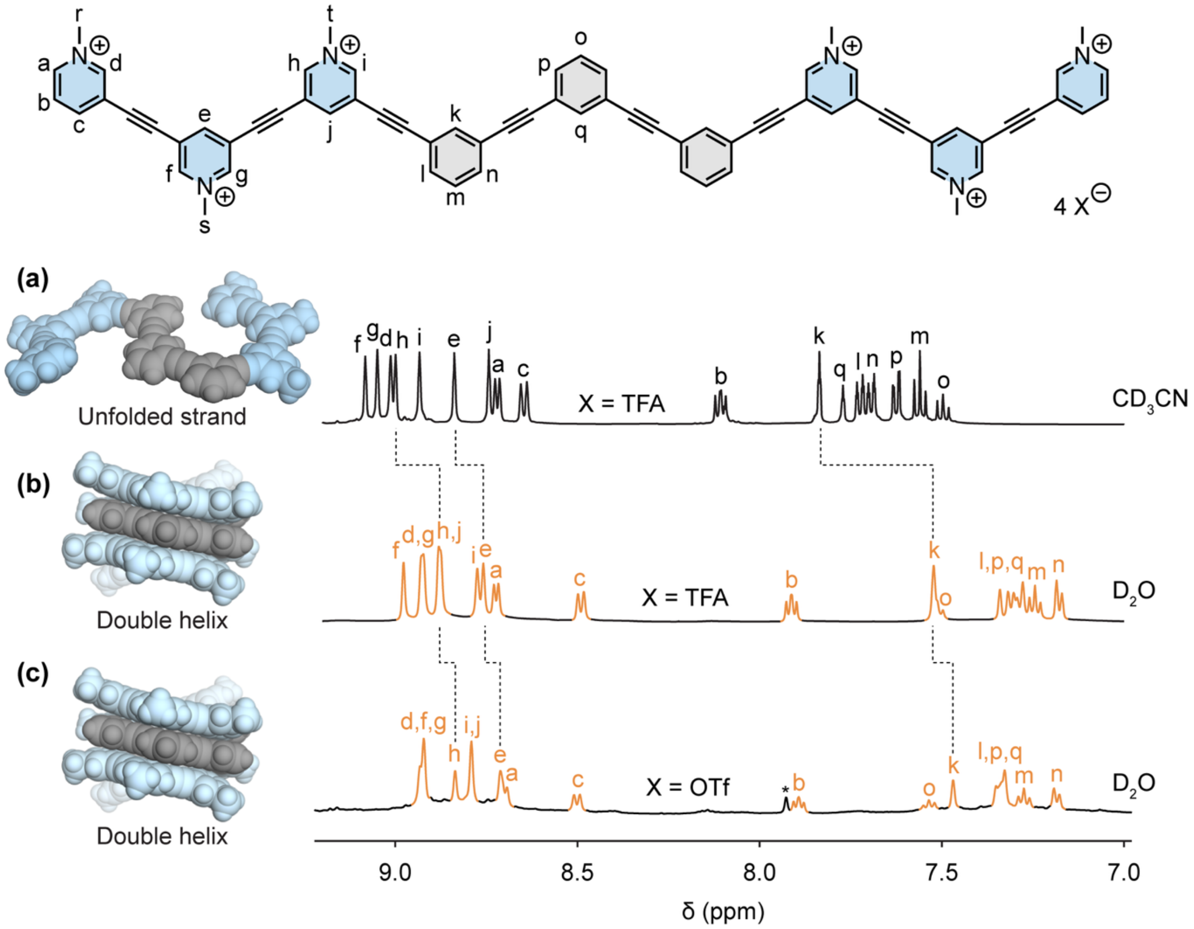

Figure S13 | Comparison between the ¹H NMR spectra of strand 1·TFA and 1·OTf. Comparison between the ¹H NMR spectra of **a,** unfolded strand **1·TFA** in CD_3CN (5 mM); **b,** double helix (**1**)$_2$·**TFA** in D_2O (5 mM); and **c,** double helix (**1**)$_2$·**OTf** in D_2O (5 mM). All the spectra were recorded at 500 MHz and 298 K. In panel **c**, an unknown impurity is labelled with a star.

5. Host-guest studies with double helix $(1)_2{\cdot}TFA$

5.1. Titration with potassium trifluoromethanesulfonate (G1)

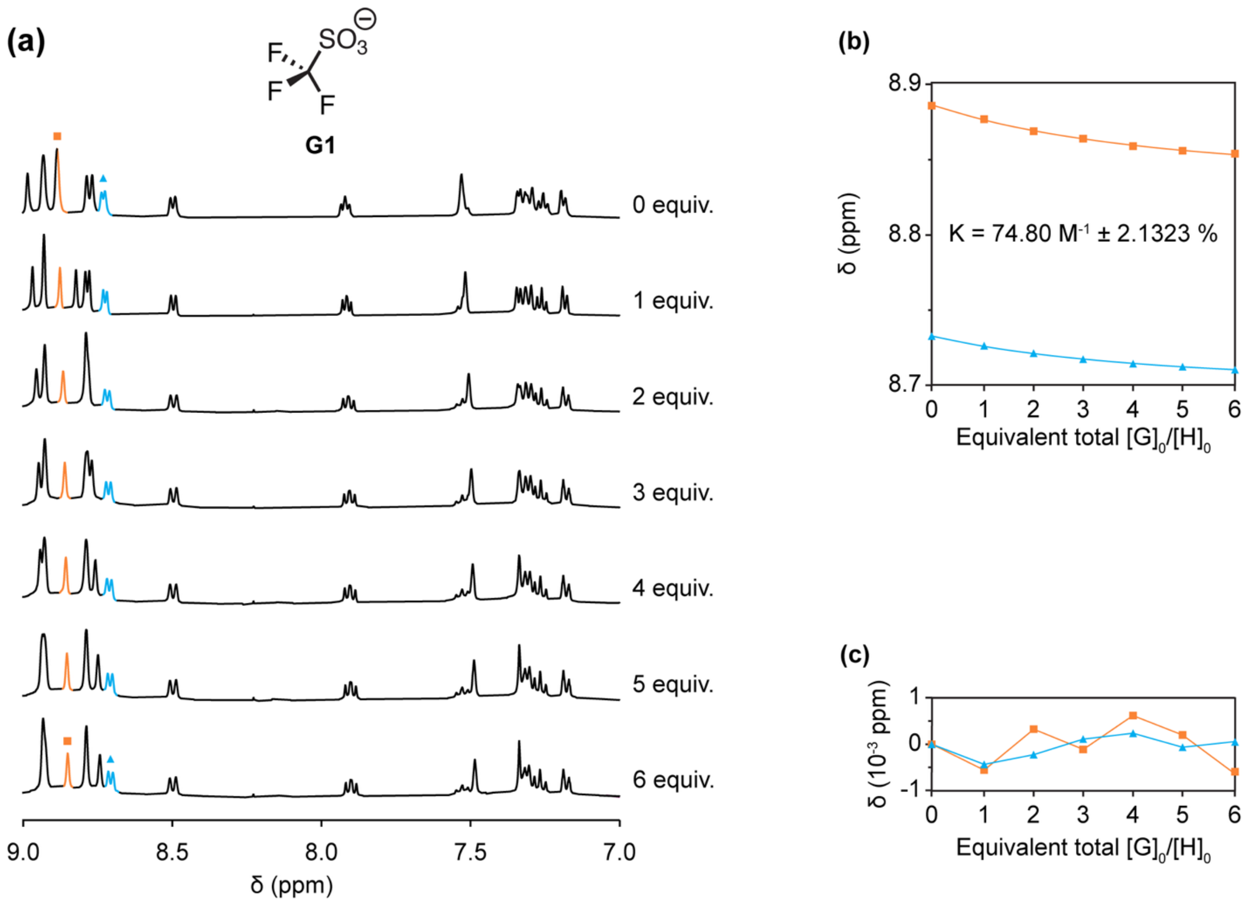

Figure S14 | ^{1}H NMR titration (400 MHz, 298 K) of potassium trifluoromethanesulfonate G1 into a 5 mM-solution of $1{\cdot}TFA$ in D_2O. a, Selected region of the ^{1}H NMR spectra recorded during the titration. **b,** Chemical shift changes recorded as a function of guest equivalents. **c,** Residuals from the curve fitting. Association constant was obtained by fitting the data with *BindFit*.[4]

5.2. Titration with potassium perfluorobutanesulfonate (G2)

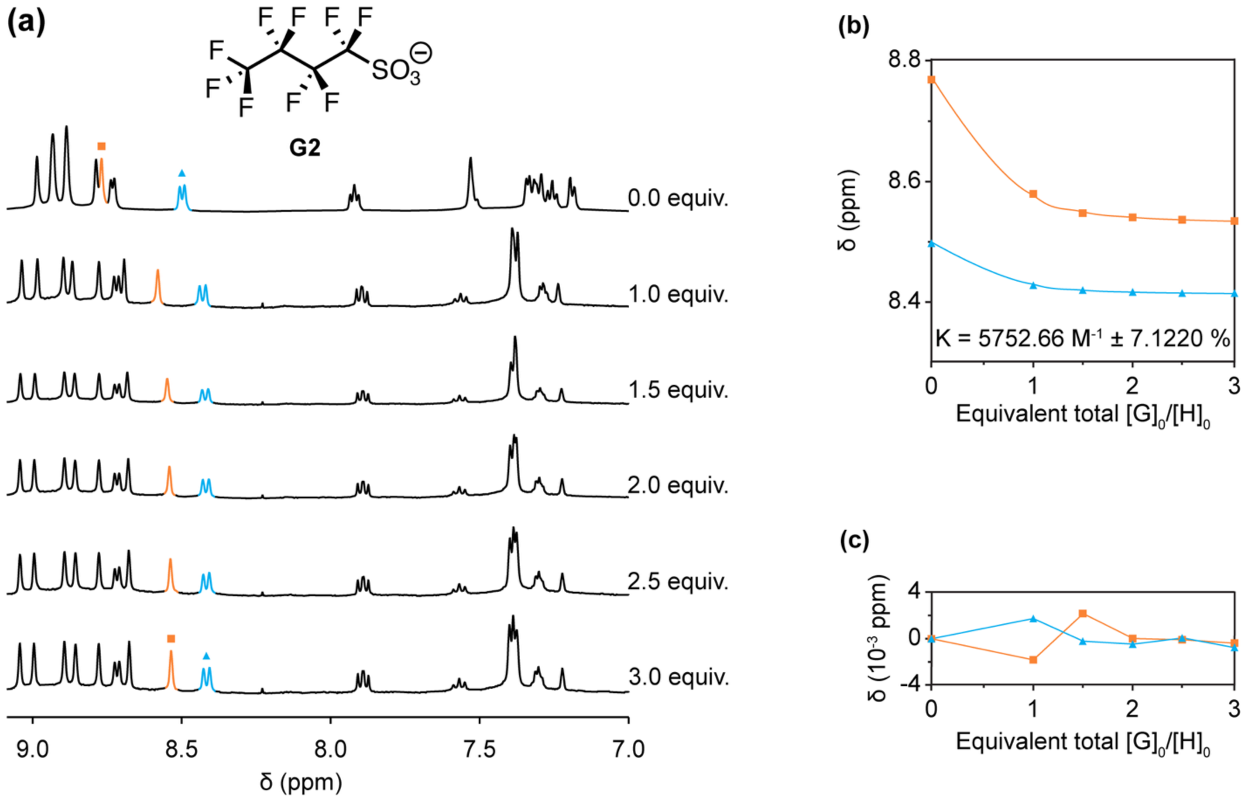

Figure S15 | ^{1}H NMR titration (400 MHz, 298 K) of potassium perfluorobutanesulfonate G2 into a 5 mM-solution of 1·TFA in D_2O. a, Selected region of the ^{1}H NMR spectra recorded during the titration. **b,** Chemical shift changes recorded as a function of guest equivalents. **c,** Residuals from the curve fitting. Association constant was obtained by fitting the data with *BindFit*.[4]

5.3. Comparison of the NMR spectra obtained after addition of guests G1-G4

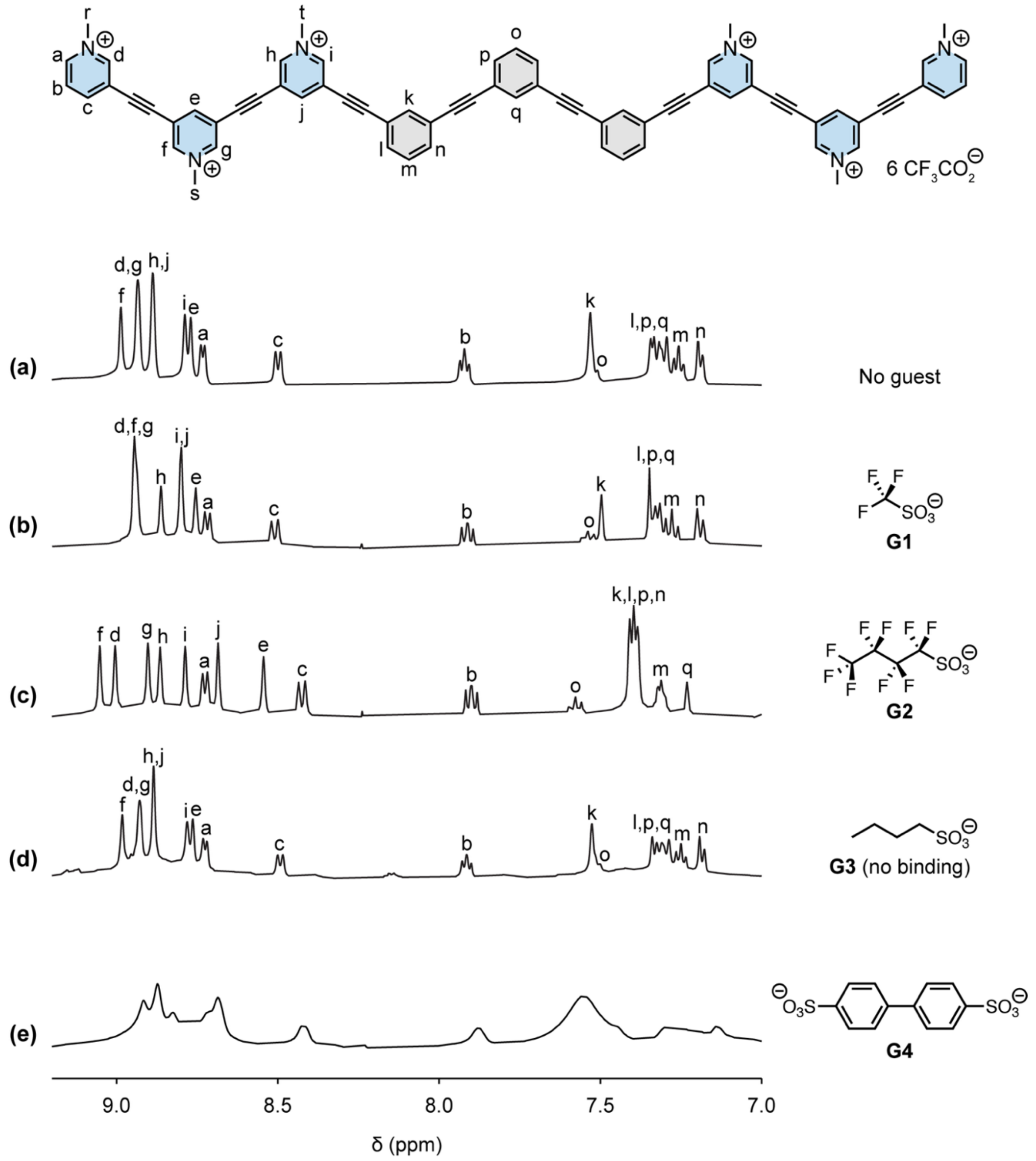

Figure S16 | ^{1}H NMR spectra of 1·TFA aromatic region (5 mM in D_2O, 500 MHz, 298 K) after addition of different guests. a, No guest added. **b,** After addition of 6.0 equiv. of potassium trifluoromethanesulfonate **G1**. **c,** After addition of 3.0 equiv. of potassium perfluorobutanesulfonate **G2**. **d,** After addition of 3.0 equiv. of sodium butanesulfonate **G3**. **e,** After addition of 2.0 equiv. of sodium 4,4'-biphenyldisulfonate **G4** (the sample precipitates above 2 equiv. of **G4**).

6. NMR Characterization of unfolded strand 2 in CD_3CN

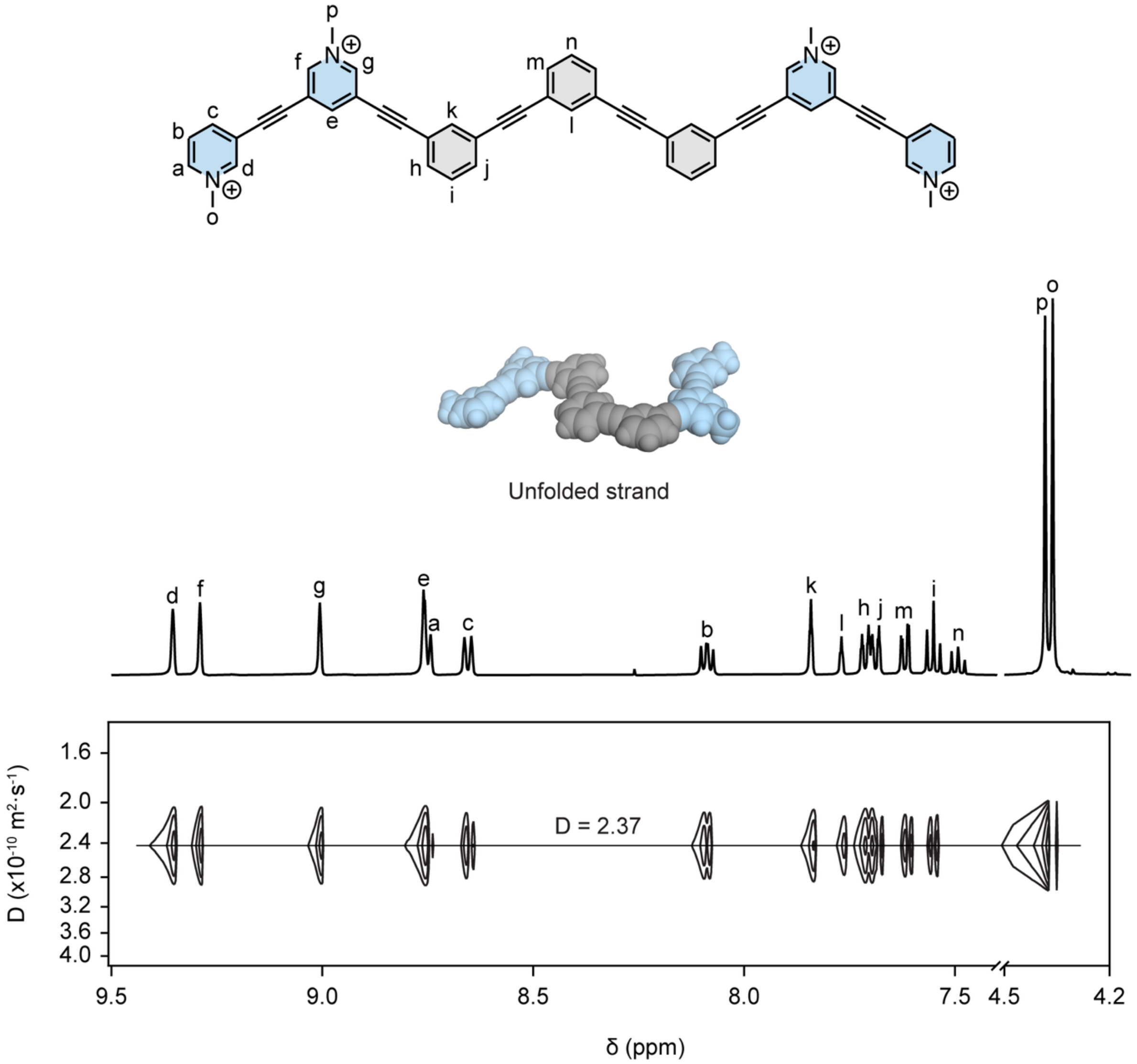

Figure S17 | Expansion of ^{1}H and DOSY NMR spectra of unfolded strand 2·TFA in CD_3CN (5 mM, 500 MHz, 298 K). Similar experiment was performed on strand 2·OTf. The hydrodynamic radii of unfolded strand 2·OTf and unfolded strand 2·TFA were found to be r_H = 10.3 Å and r_H = 10.8 Å, respectively, using the Stokes-Einstein equation.

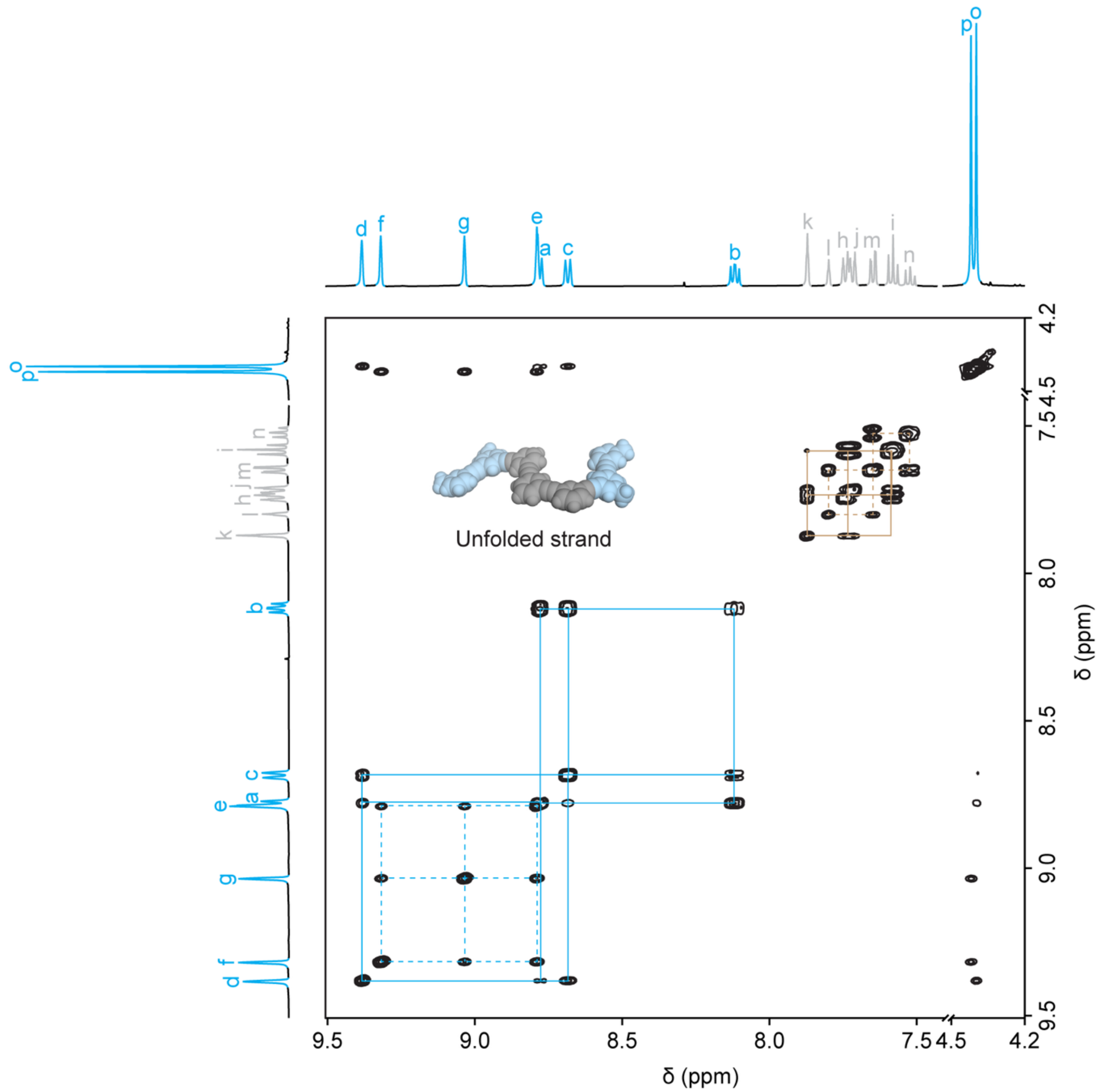

Figure S18 | Expansion of selected regions in 2D ^{1}H-^{1}H COSY NMR spectrum of unfolded strand 2·TFA in CD_3CN (5 mM, 500 MHz, 298 K).

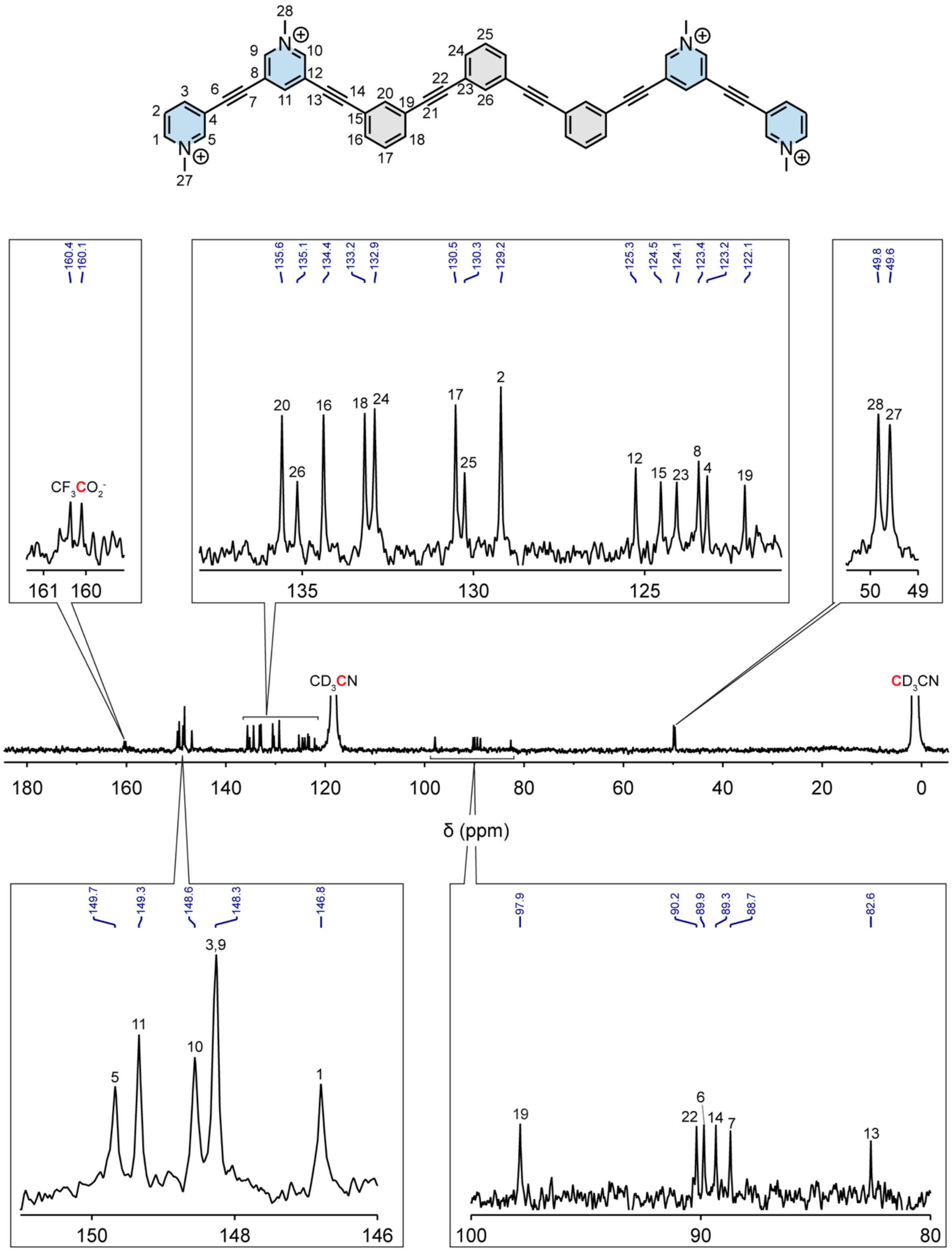

Figure S19 | ^{13}C NMR spectrum of unfolded strand 2·TFA in CD_3CN (5 mM, 126 MHz, 298 K).

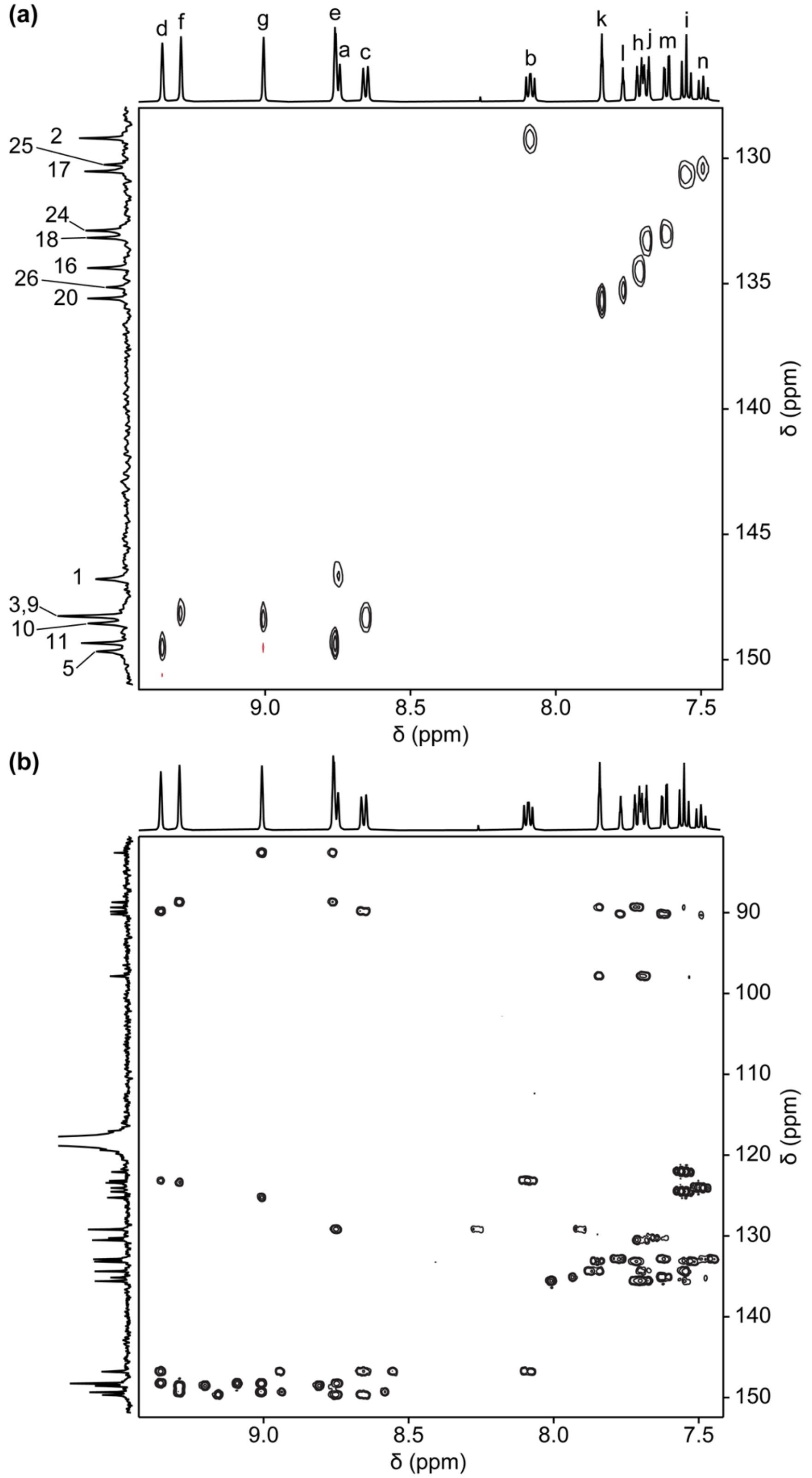

Figure S20 | a, ^{1}H-^{13}C HSQC and b, ^{1}H-^{13}C HMBC NMR spectra of unfolded strand 2·TFA in CD_3CN (5 mM, 500 MHz, 298 K).

7. NMR characterization of double helix $(2)_2$ and triple helix $(2)_3$ in D_2O

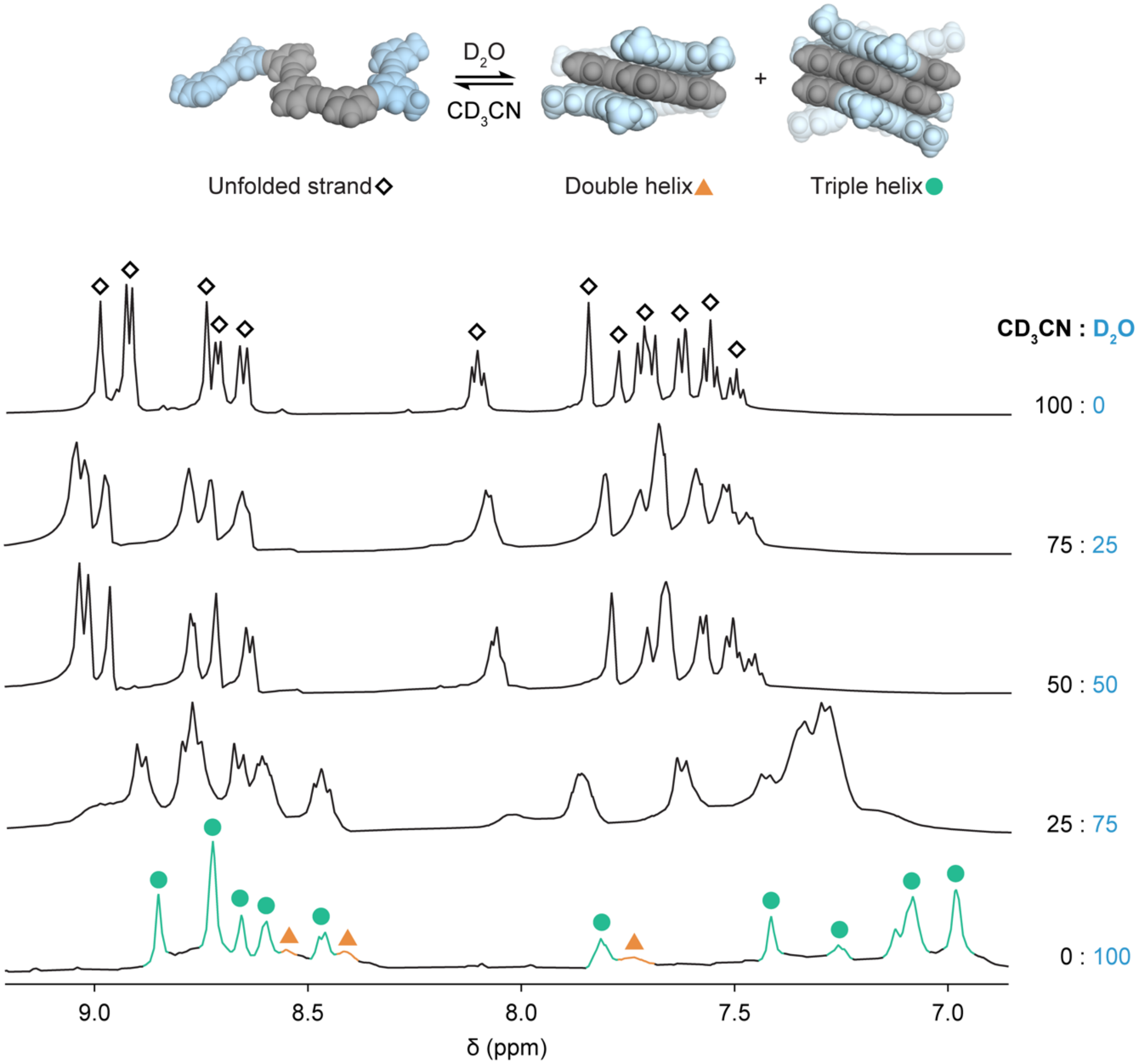

Figure S21 | ^{1}H NMR spectra of 2·OTf aromatic protons in CD_3CN / D_2O mixtures (5 mM, 500 MHz, 298 K). The signals corresponding to the unfolded strand, double helix and triple helix are labelled with empty diamonds, orange triangles and green circles, respectively.

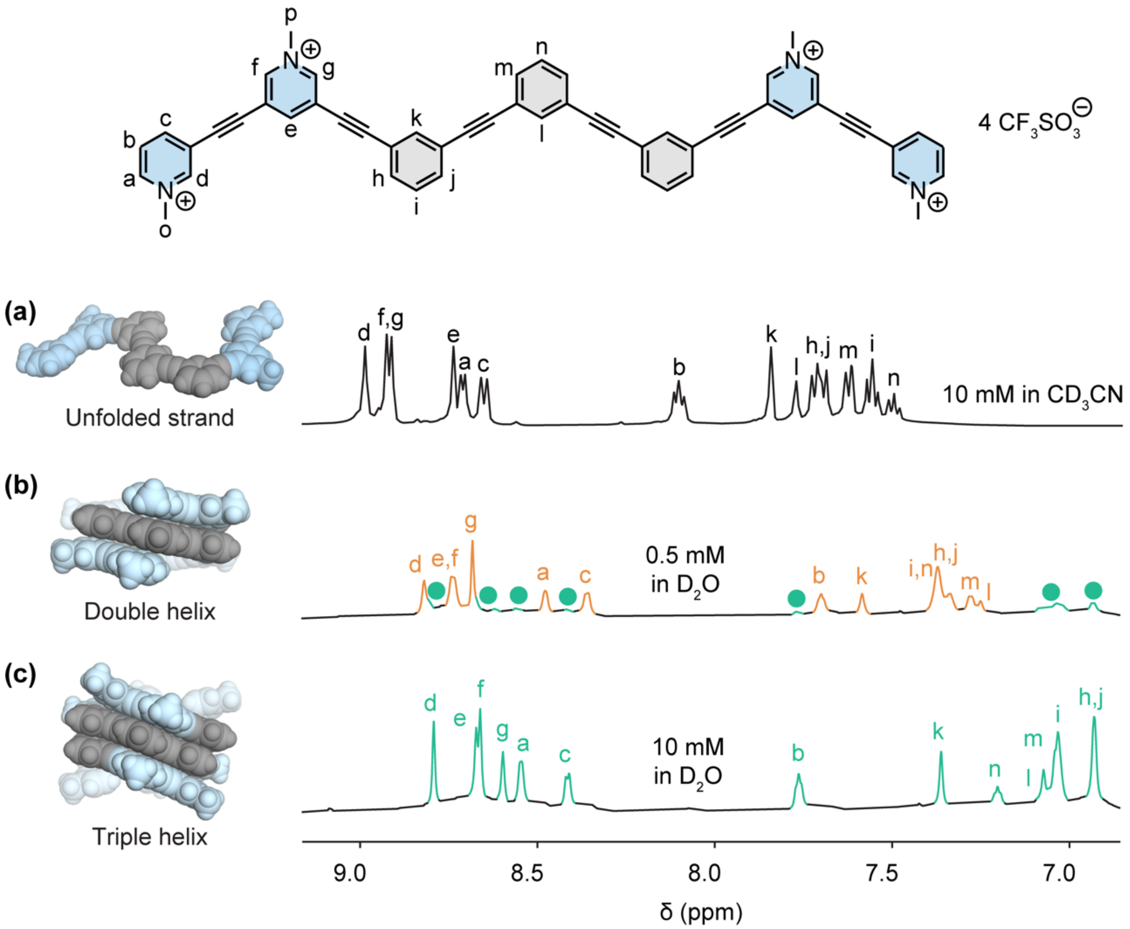

Figure S22 | Solvent-dependent ^{1}H NMR spectra of strand 2. Comparison between the ^{1}H NMR spectra of **a,** unfolded strand **2·OTf** in CD_3CN (10 mM, 500 MHz, 298 K); **b,** double helix (**2**)$_2$**·OTf** in D_2O (0.5 mM, 800 MHz, 288 K); and **c,** triple helix (**2**)$_3$**·OTf** in D_2O (10 mM, 800 MHz, 288 K). In spectrum **b**, the signals corresponding to the triple helix are labelled with green circles.

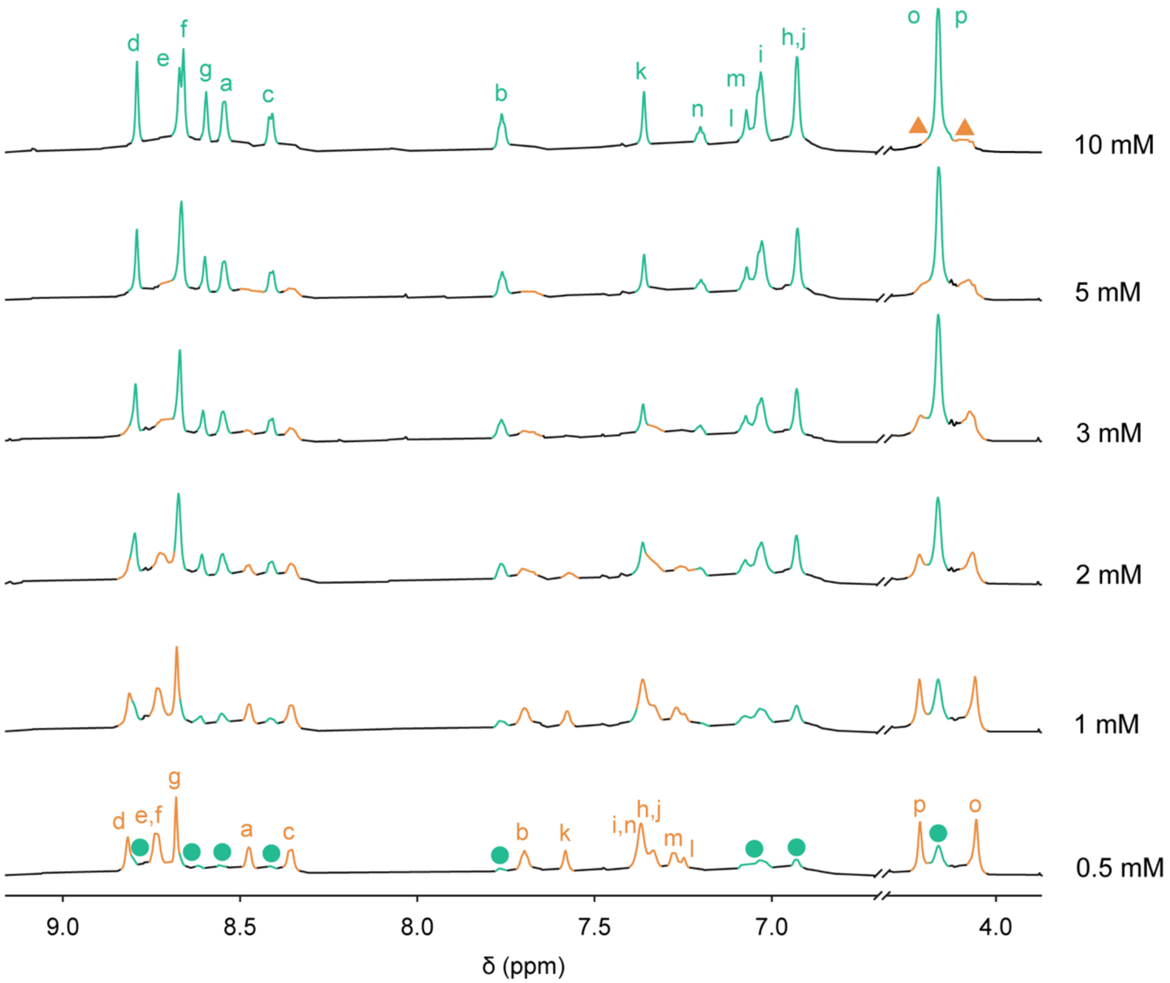

Figure S23 | Concentration-dependent ^{1}H NMR spectra of 2·OTf between 0.5 mM and 10 mM in D_2O (800 MHz, 288 K). The triple helix is the main species observed at high concentration and dissociates into the double helix upon dilution.

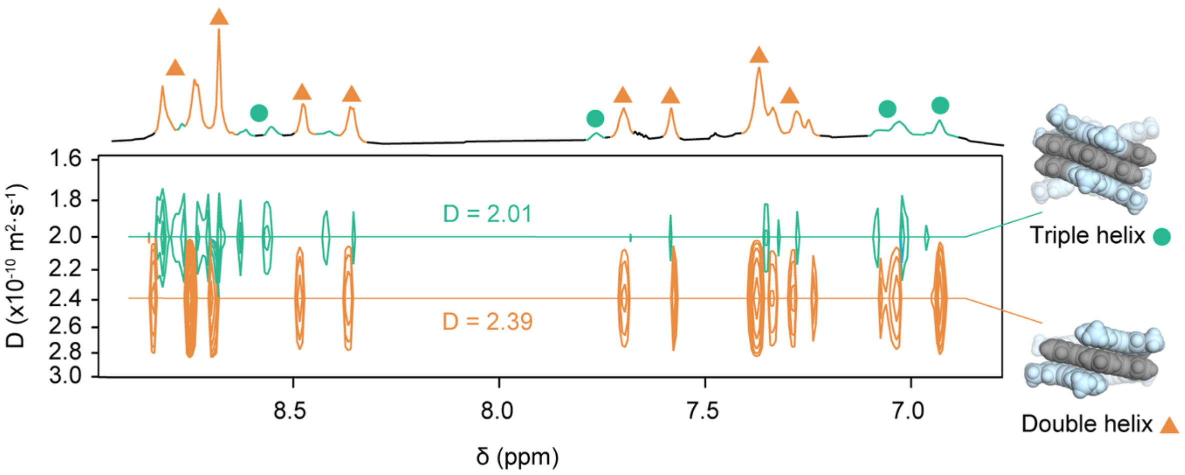

Figure S24 | ^{1}H DOSY NMR spectrum (800 MHz, D_2O, 288 K) of the double and triple helices assembled from 2·OTf (0.5 mM) in D_2O. The signals corresponding to the double and triple helices are labelled with orange triangles and green circles, respectively. The hydrodynamic radii of the double and triple helices were found to be r_H = 8.1 Å and r_H = 9.4 Å, respectively, using the Stokes-Einstein equation.

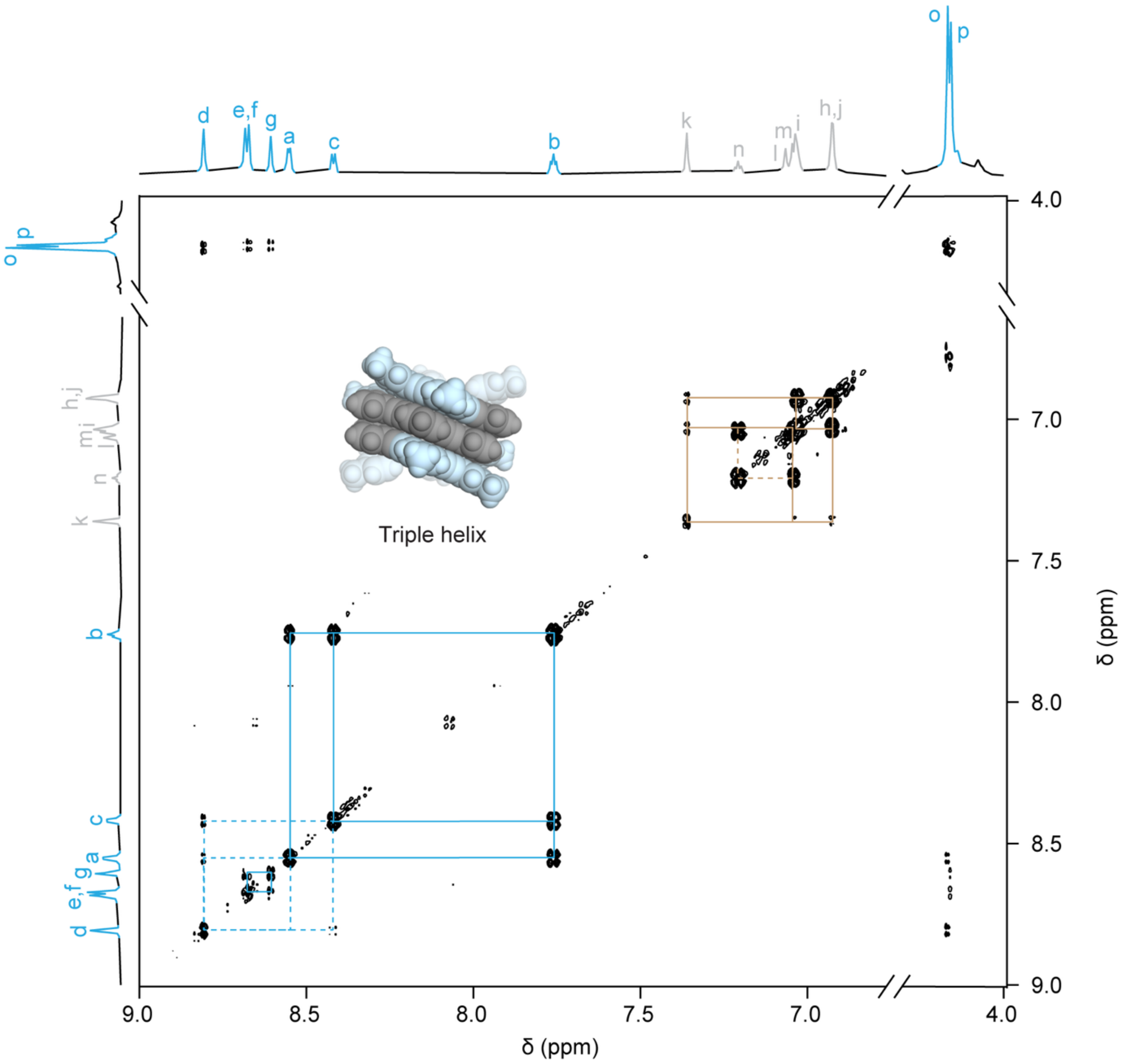

Figure S25 | 2D ^1H-^1H COSY from selected spectral regions of triple helix $(2)_3{\cdot}OTf$ in D_2O (10 mM, 800 MHz, 288 K).

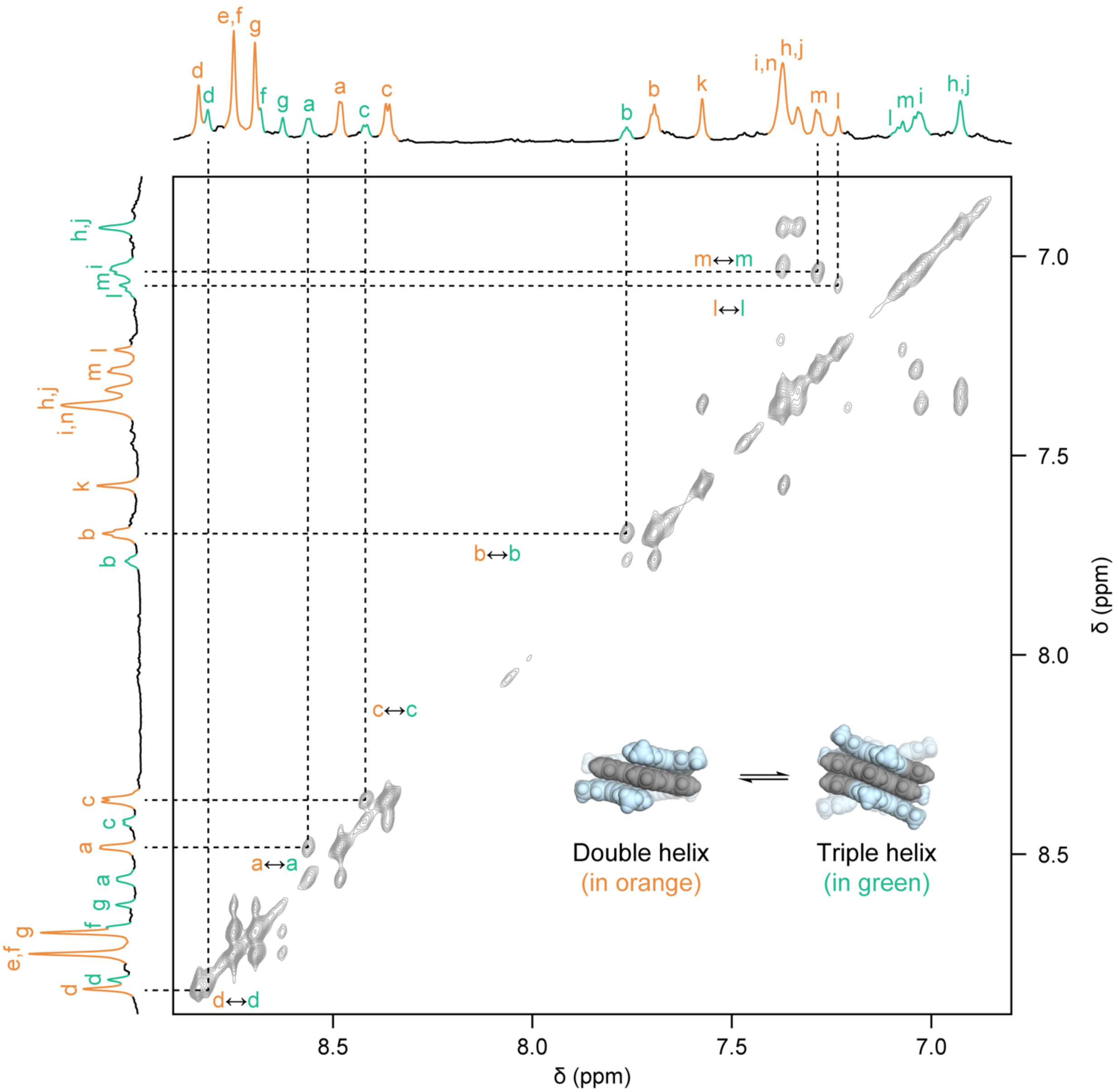

Figure S26 | 2D ^{1}H-^{1}H ROESY NMR spectrum from aromatic region of 2·OTf at 0.5 mM in D_2O (800 MHz, 288 K, 200 ms mixing time). The dotted lines highlight exchange cross-peaks between the protons of the double and triple helices, colored in orange and green, respectively. The ROE cross-peak correlations are shown on the following figures.

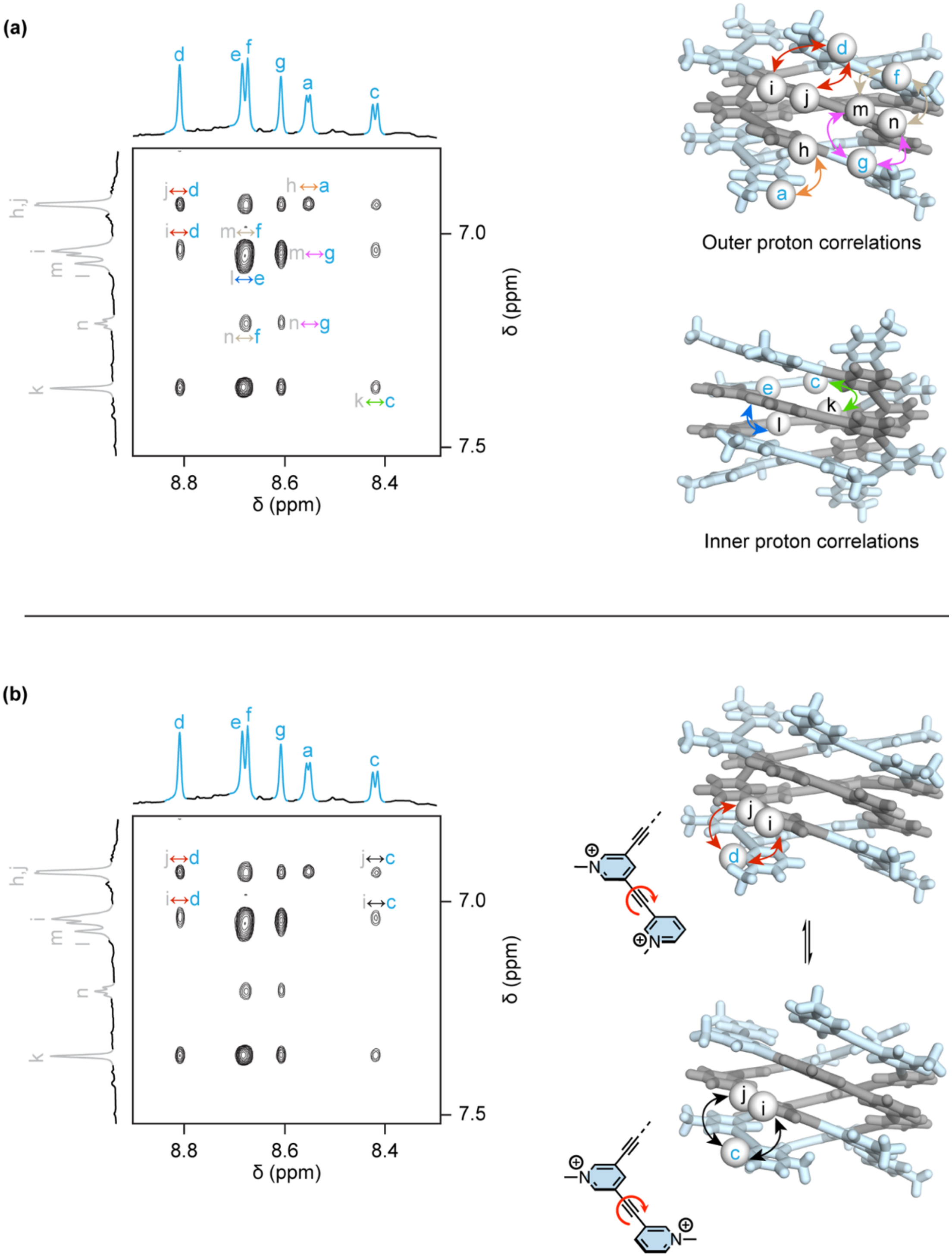

Figure S27 | Expansion of aromatic region in 2D ^{1}H-^{1}H ROESY NMR spectrum of triple helix $(2)_3$·OTf in D_2O (10 mM, 800 MHz, 288 K, 200 ms mixing time). a, Highlighted key correlations between protons of stacked pyridinium (colored in blue) and phenylene (colored in gray) residues. **b,** Rotation of the terminal pyridinium residue around the acetylene bond results in the appearance of additional 2D correlations between stacked pyridinium and phenylene residues.

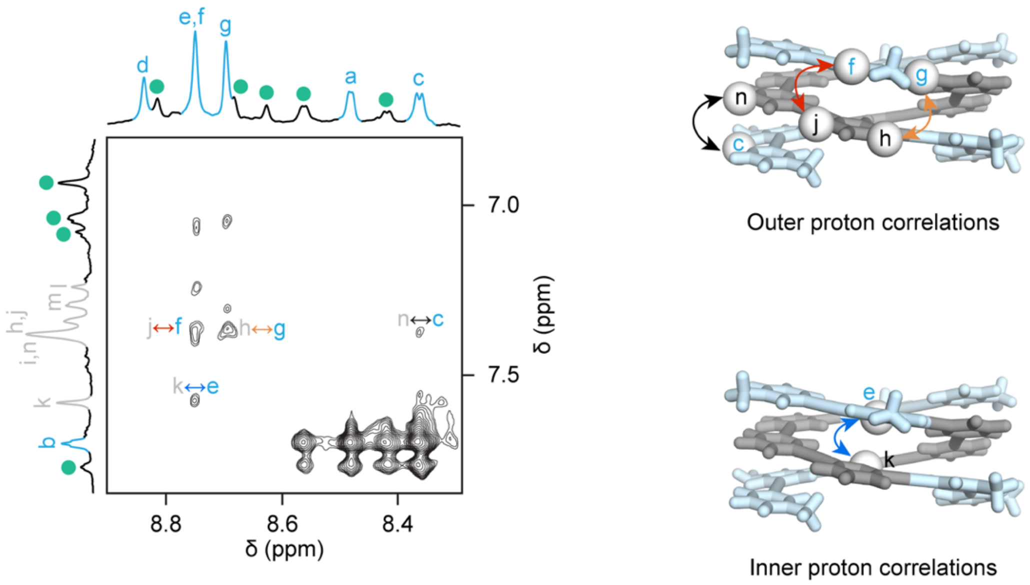

Figure S28 | Aromatic region of 2D ^{1}H-^{1}H ROESY NMR spectrum of double helix $(2)_2$·OTf in D_2O (0.5 mM, 800 MHz, 288 K, 200 ms mixing time). Key correlations between protons of stacked pyridinium (colored in blue) and phenylene (colored in gray) residues are highlighted. The signals corresponding to the triple helix are labelled with green circles.

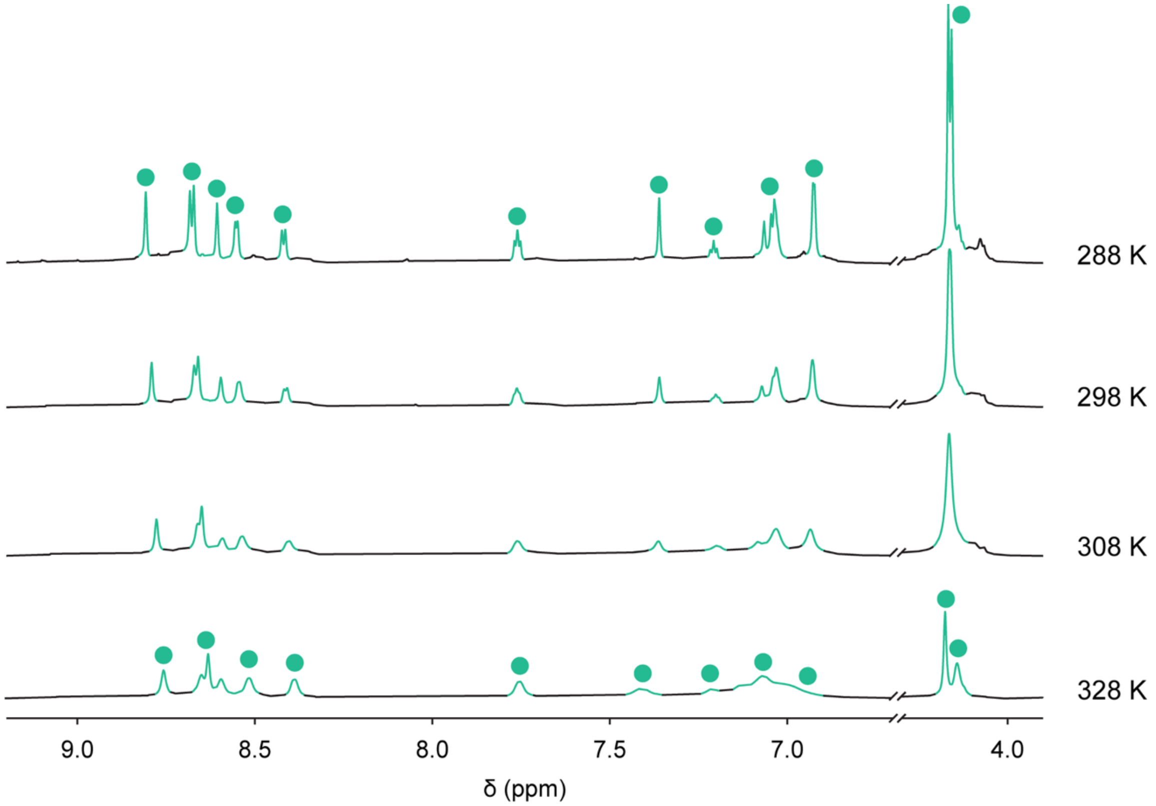

Figure S29 | Variable temperature ^{1}H NMR spectra of 2·OTf between 288 K and 328 K in D_2O (800 MHz, 10 mM). The triple helix (signals labelled with green circles) remains intact at high temperature when strand concentration is sufficiently high.

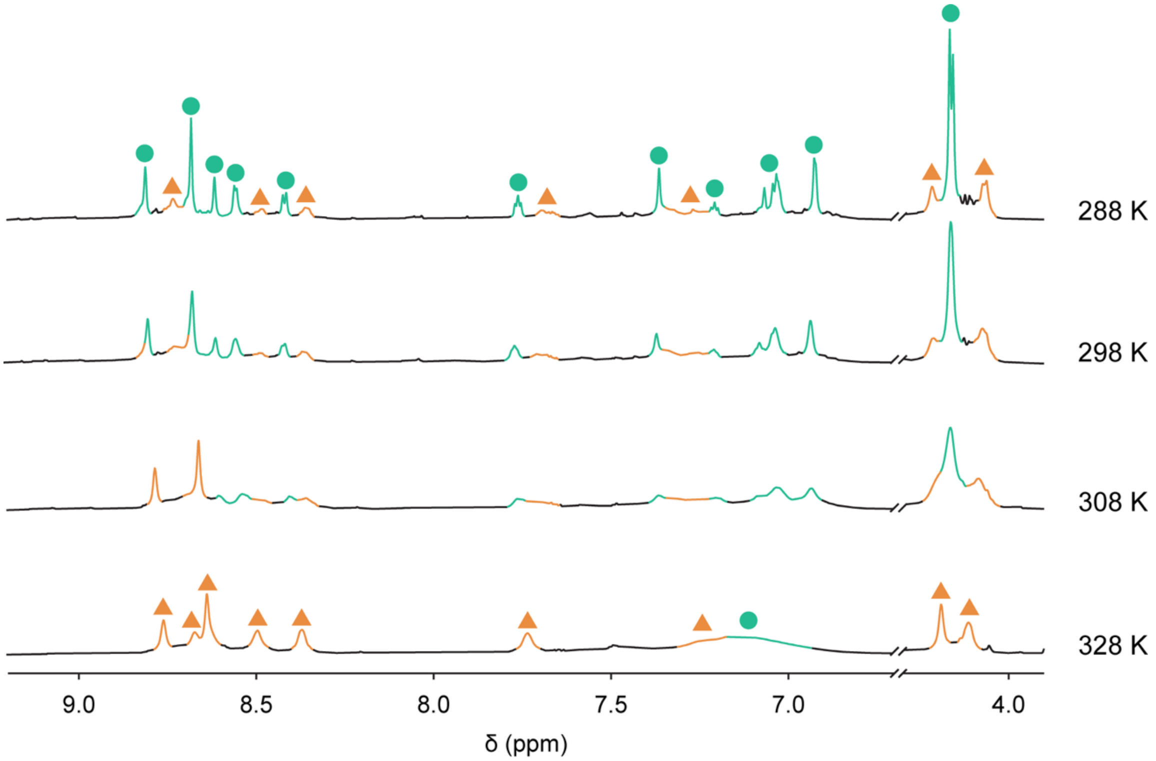

Figure S30 | Variable temperature ^{1}H NMR spectra of 2·OTf between 288 K and 328 K in D_2O (800 MHz, 3 mM). The spectra show the temperature-dependent interconversion between double and triple helix (signals labelled with orange triangles and green circles, respectively) at low strand concentration.

8. Host-guest studies with triple helix $(2)_3$·TFA

8.1. Titration with potassium trifluoromethanesulfonate (G1)

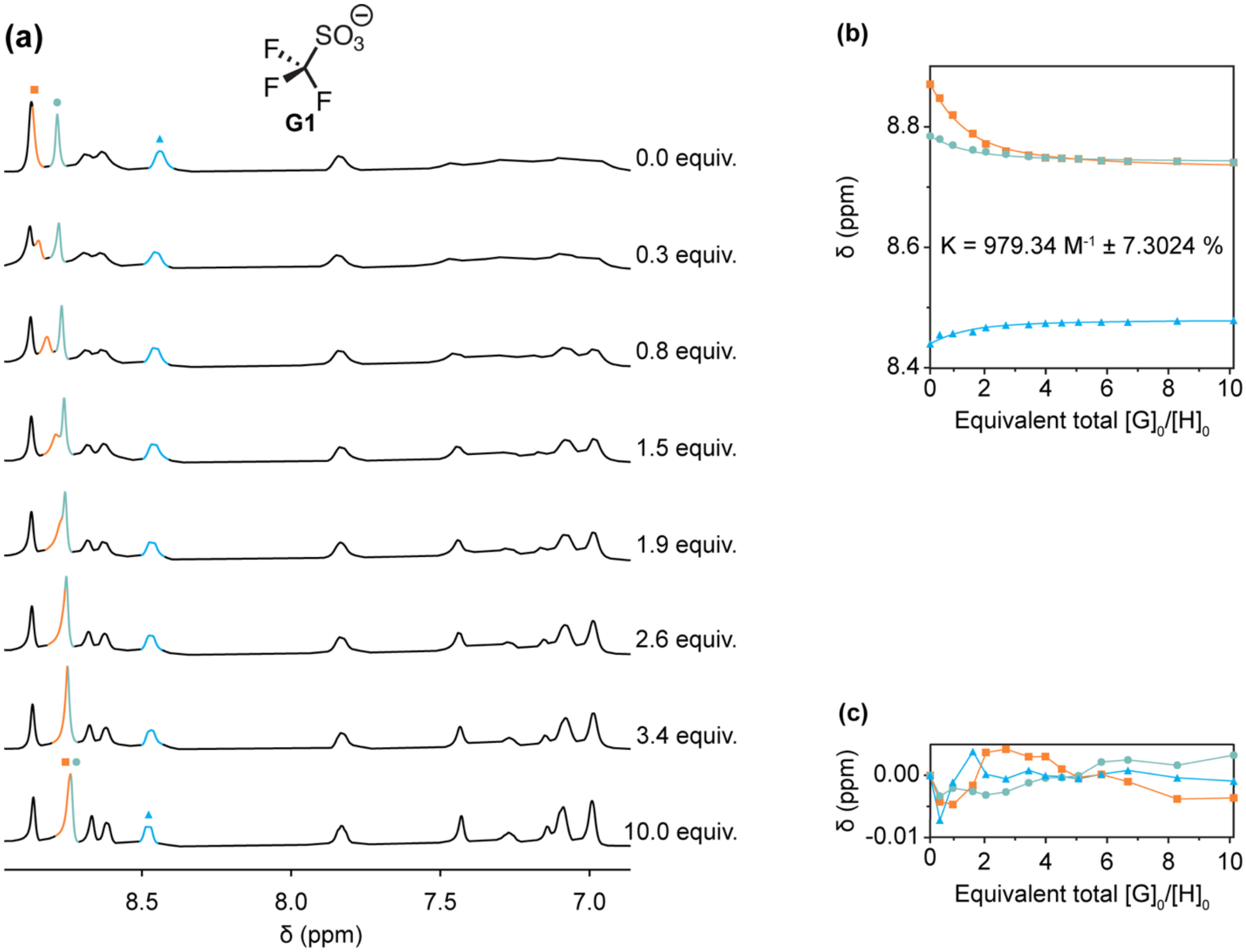

Figure S31 | ^{1}H NMR titration (500 MHz, 298 K) of potassium trifluoromethanesulfonate G1 into a 5 mM-solution of 2·TFA in D_2O. a, Selected region of the ^{1}H NMR spectra recorded during the titration. **b,** Chemical shift changes recorded as a function of guest equivalents. **c,** Residuals from the curve fitting. Association constant was obtained by fitting the data with *BindFit*.[4]

8.2. Titration with potassium perfluorobutanesulfonate (G2)

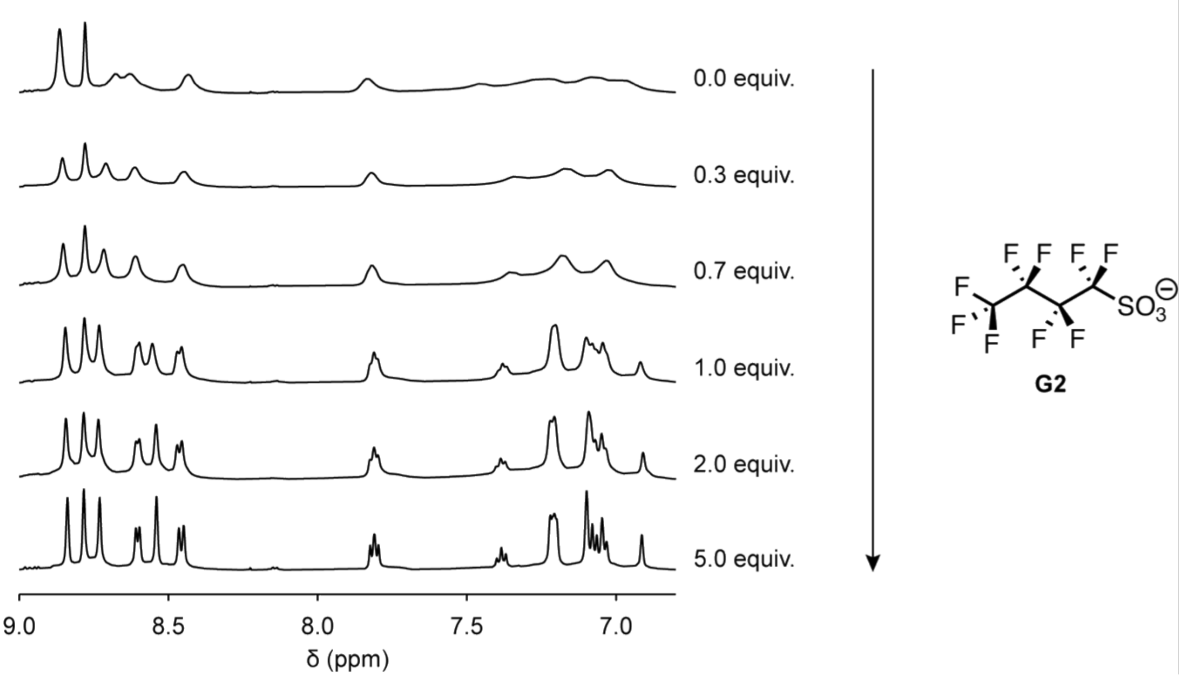

Figure S32 | ^{1}H NMR titration (500 MHz, 298 K) of potassium perfluorobutanesulfonate G2 into a 5 mM-solution of 2·TFA in D_2O.

8.3. Titration with sodium 4,4'-biphenyldisulfonate (G4)

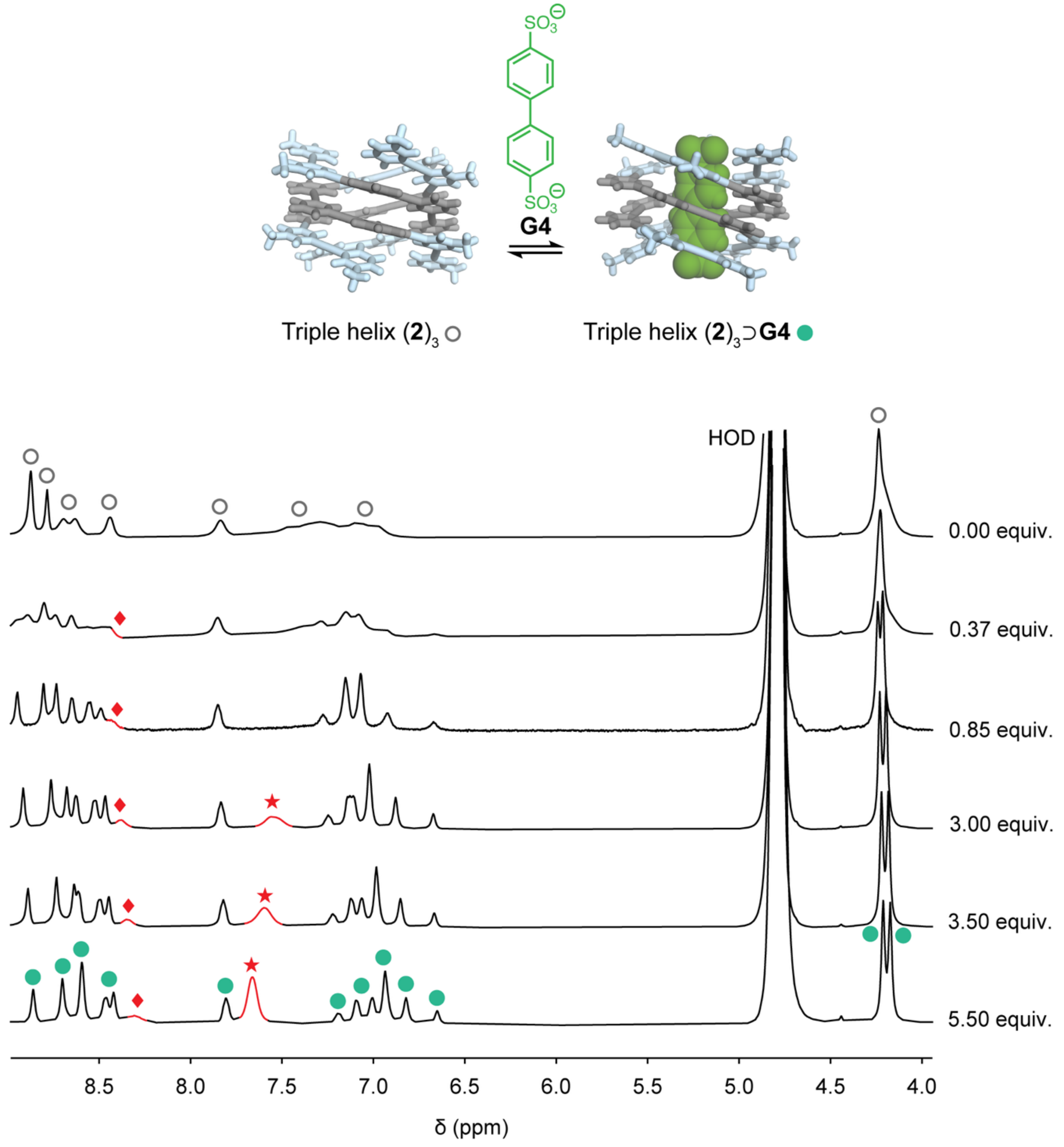

Figure S33 | ^{1}H NMR titration (500 MHz, 298 K) of sodium 4,4'-biphenyldisulfonate G4 into a 5 mM-solution 2·TFA in D_2O. The NMR signals of the resulting complex remain relatively broad, presumably due to dynamic deformations of the triple helix induced by electrostatic attraction between **G4** and the pyridinium units, as evidenced by DFT modeling. This bulky guest appears to occupy the cavity more completely than **G2**, slowing the exchange between free and bound guests on the NMR timescale. The signals corresponding to the bound and free guest are labelled with a red diamond and a red star, respectively.

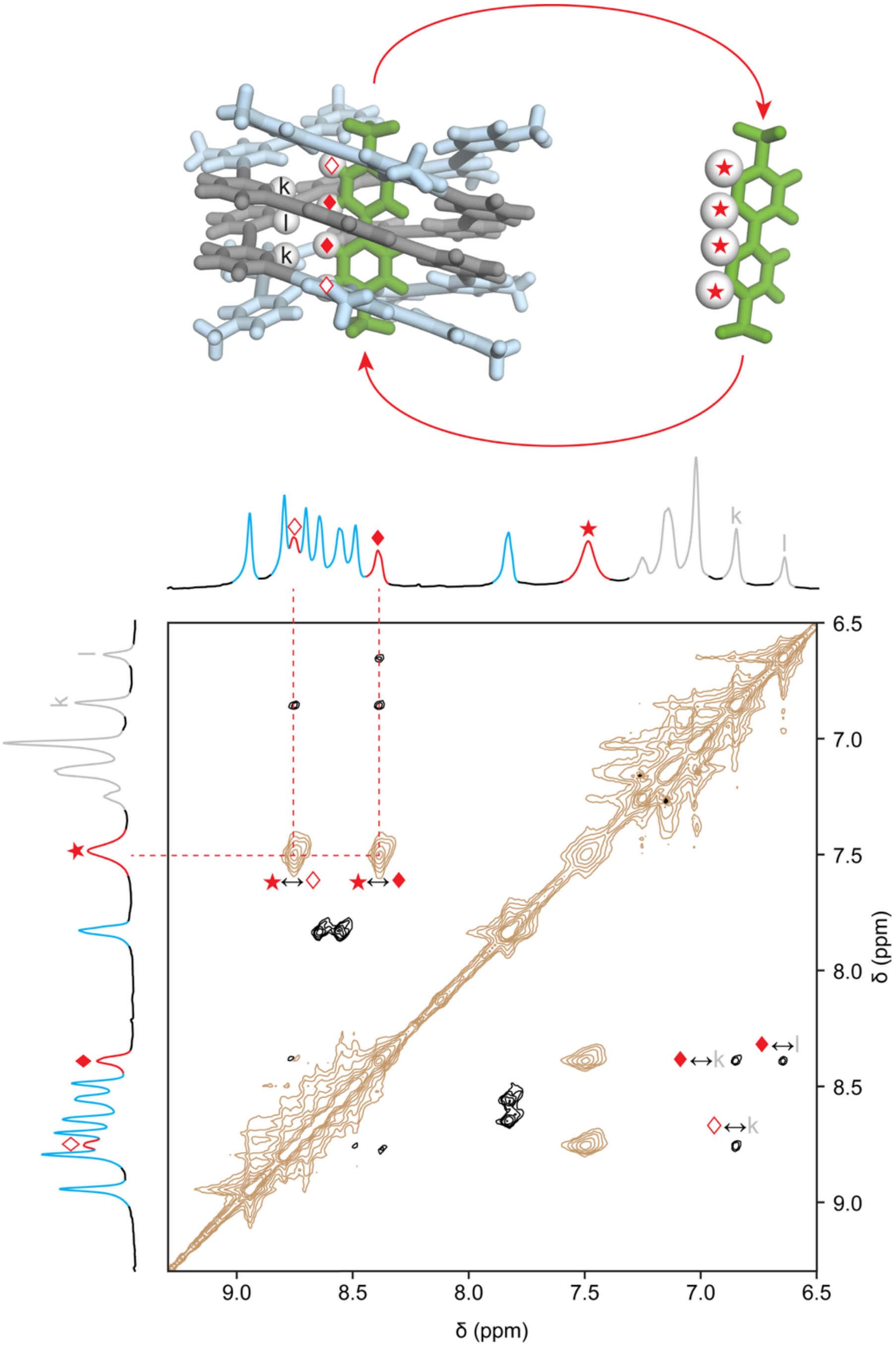

Figure S34 | Expansion of selected spectral regions from 2D ^{1}H-^{1}H ROESY of 2·TFA at 5 mM in D_2O (500 MHz, 100 ms mixing time) recorded at low temperature (278 K) in the presence of 5.5 equiv. of sodium 4,4'-biphenyldisulfonate G4. The dotted lines highlight exchange cross-peaks bound and free guest protons, labelled with red diamonds and star, respectively. ROE correlations between the bound guest and the inner phenylene protons of the triple helix (*k* and *l*) are also observable.

8.4. Comparison of the NMR spectra obtained after addition of guests G1-G4

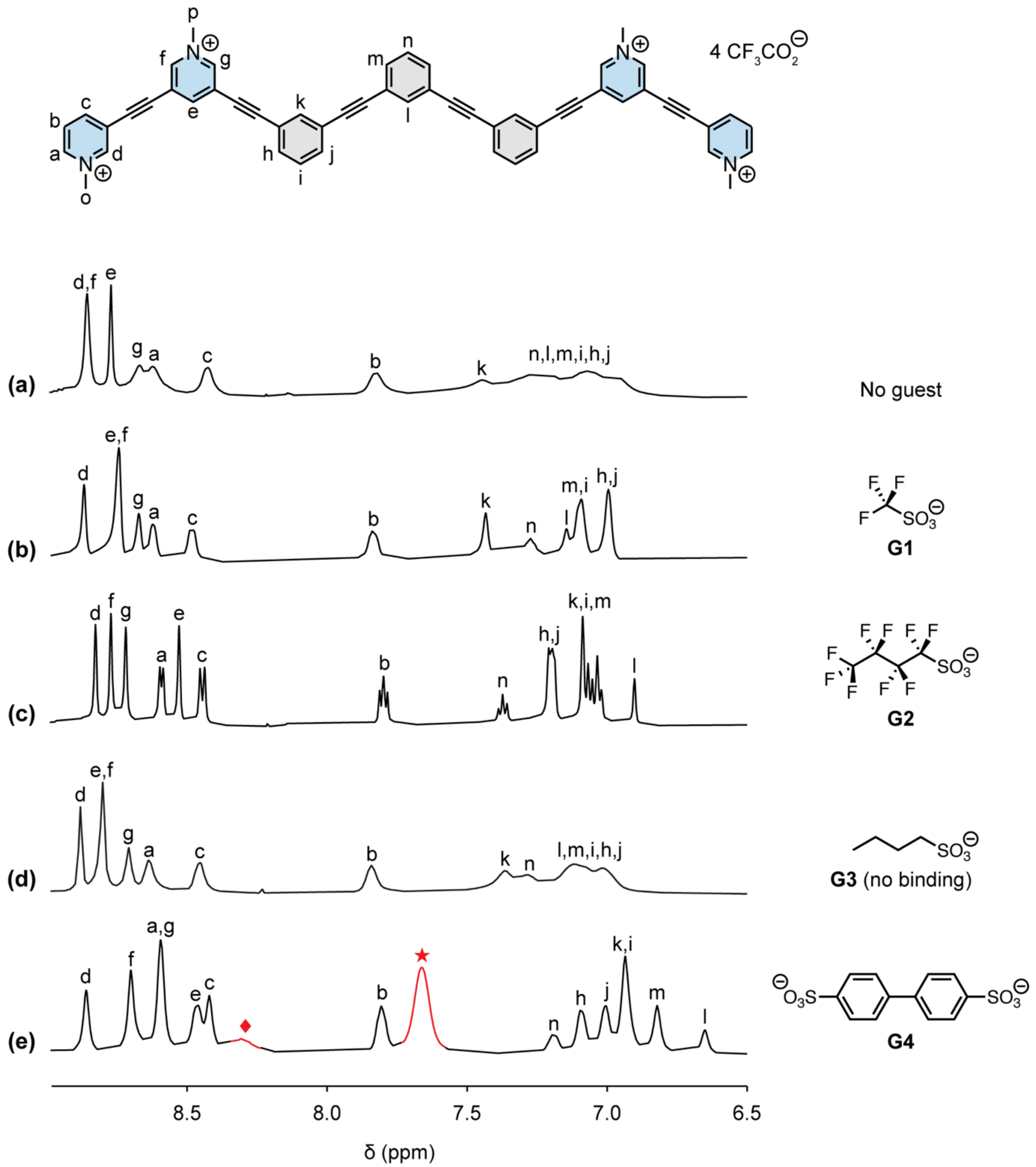

Figure S35 | Aromatic region of ^{1}H NMR spectra of 2·TFA (5 mM in D_2O, 500 MHz, 298 K) after addition of different guests. a, No guest added. **b,** After addition of 10.0 equiv. of potassium trifluoromethanesulfonate **G1**. **c,** After addition of 5.0 equiv. of potassium perfluorobutanesulfonate **G2**. **d,** After addition of 3.9 equiv. of sodium butanesulfonate **G3**. **e,** After addition of 5.5 equiv. of sodium 4,4’-biphenyldisulfonate **G4**. The signals corresponding to bound and free guest **G4** are labelled with a red diamond and a star, respectively.

9. Concentration- and temperature-dependent double-to-triple helix transformation in the presence of guests G1-G4

9.1. Concentration- and temperature-dependent double-to-triple helix transformation in the presence of potassium trifluoromethanesulfonate (G1)

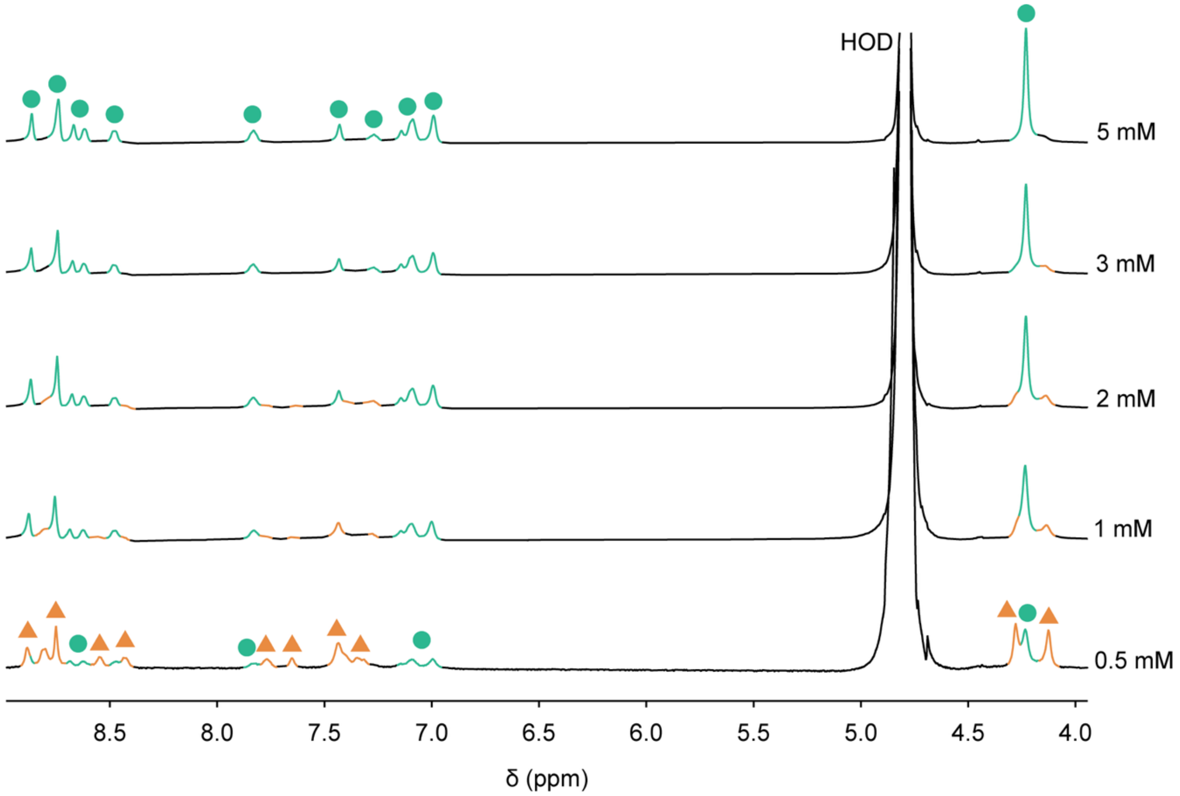

Figure S36 | Concentration-dependent ^{1}H NMR spectra of 2·TFA between 0.5 mM and 5 mM in D_2O (500 MHz, 298 K) in the presence of 10 equiv. of potassium trifluoromethanesulfonate G1. The signals corresponding to the double and triple helices are labelled with orange triangles and green circles, respectively.

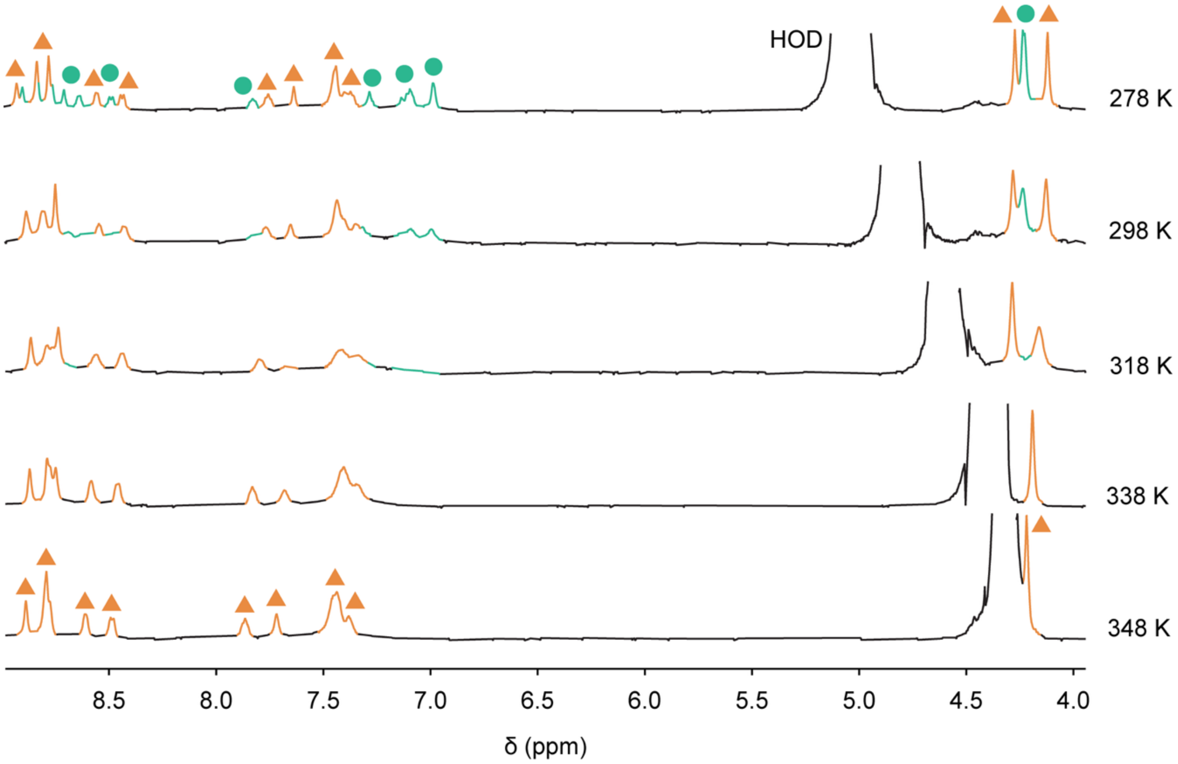

Figure S37 | Variable temperature ^{1}H NMR spectra of 2·TFA between 278 K and 348 K in D_2O (500 MHz, 0.5 mM) in the presence of 10 equiv. of potassium trifluoromethanesulfonate G1. The signals corresponding to the double and triple helices are labelled with orange triangles and green circles, respectively.

9.2. Concentration- and temperature-dependent double-to-triple helix transformation in the presence of potassium perfluorobutanesulfonate (G2)

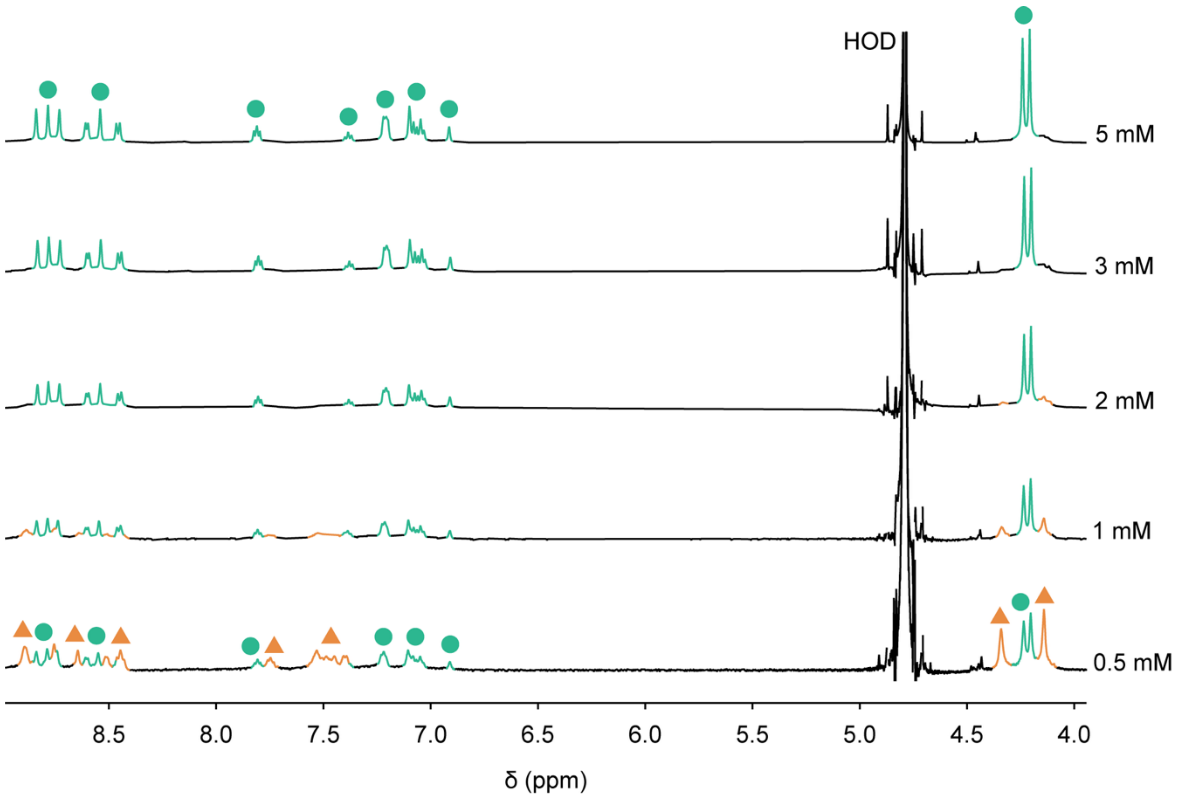

Figure S38 | Concentration-dependent ^{1}H NMR spectra of 2·TFA between 0.5 mM and 5 mM in D_2O (500 MHz, 298 K) in the presence of 5 equiv. of potassium perfluorobutanesulfonate G2. The signals corresponding to the double and triple helices are labelled with orange triangles and green circles, respectively.

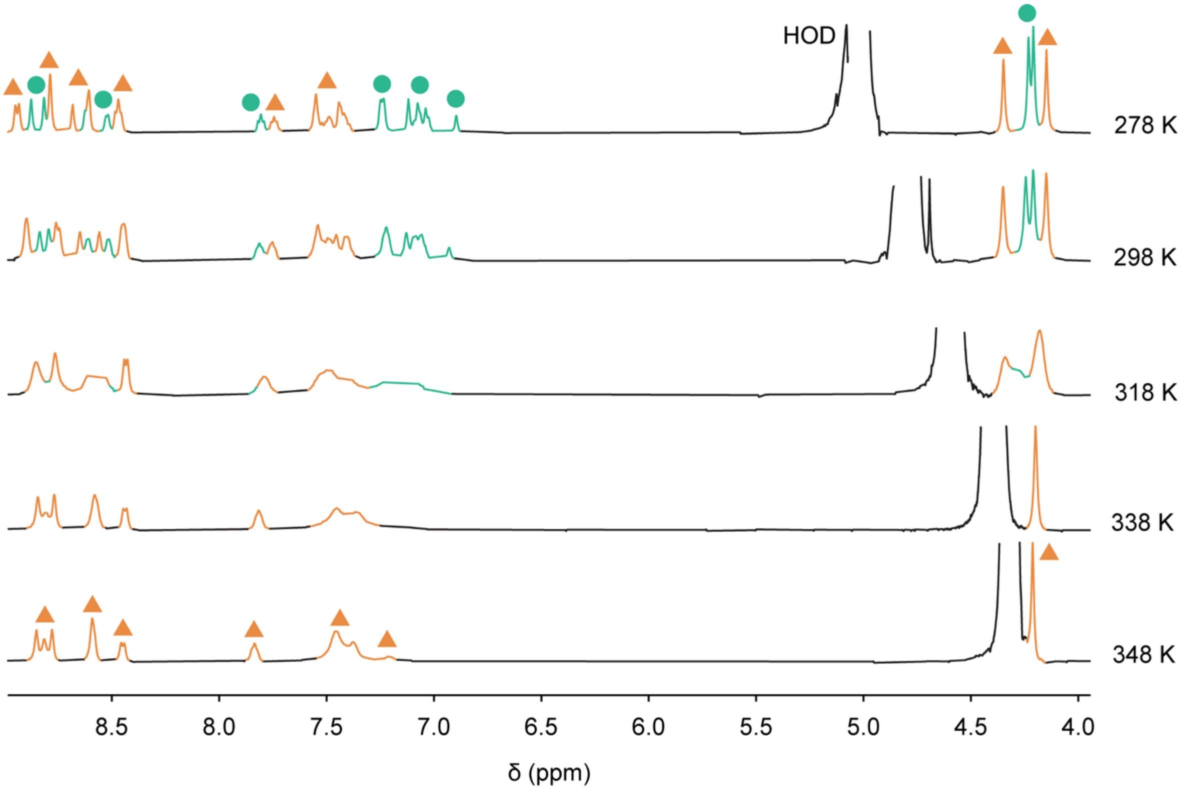

Figure S39 | Variable temperature ^{1}H NMR spectra of 2·TFA between 278 K and 348 K in D_2O (500 MHz, 0.5 mM) in the presence of 5 equiv. of potassium perfluorobutanesulfonate G2. The signals corresponding to the double and triple helices are labelled with orange triangles and green circles, respectively.

9.3. Concentration- and temperature-dependent double-to-triple helix transformation in the presence of sodium 4,4'-biphenyldisulfonate guest (G4)

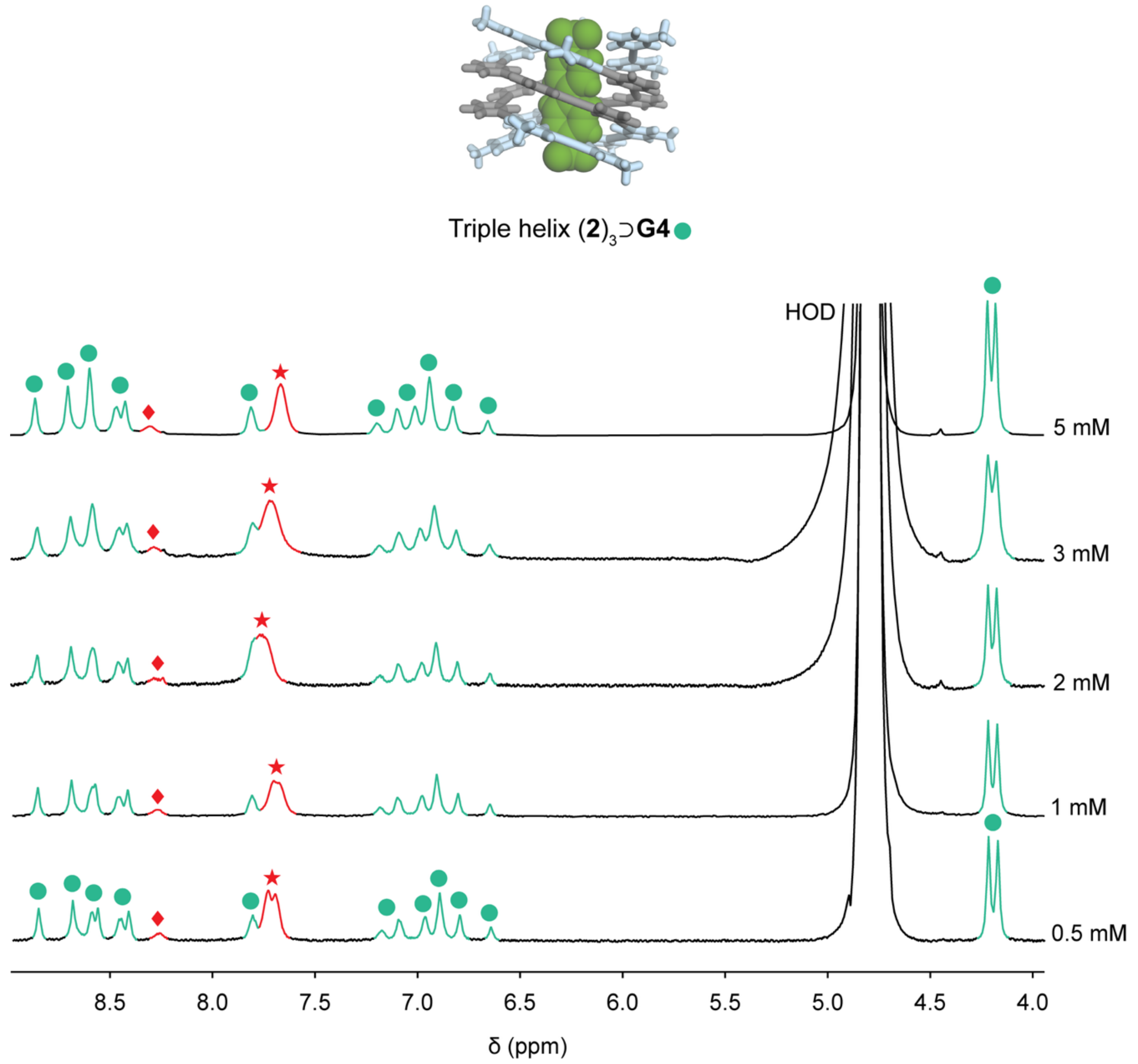

Figure S40 | Concentration-dependent ^{1}H NMR spectra of 2·TFA between 0.5 mM and 5 mM in D_2O (500 MHz, 298 K) in the presence of 5.5 equiv. of sodium 4,4'-biphenyldisulfonate G4. No switch was observed in the conditions. The signals corresponding to the bound and free guest are labelled with a red diamond and a red star, respectively.

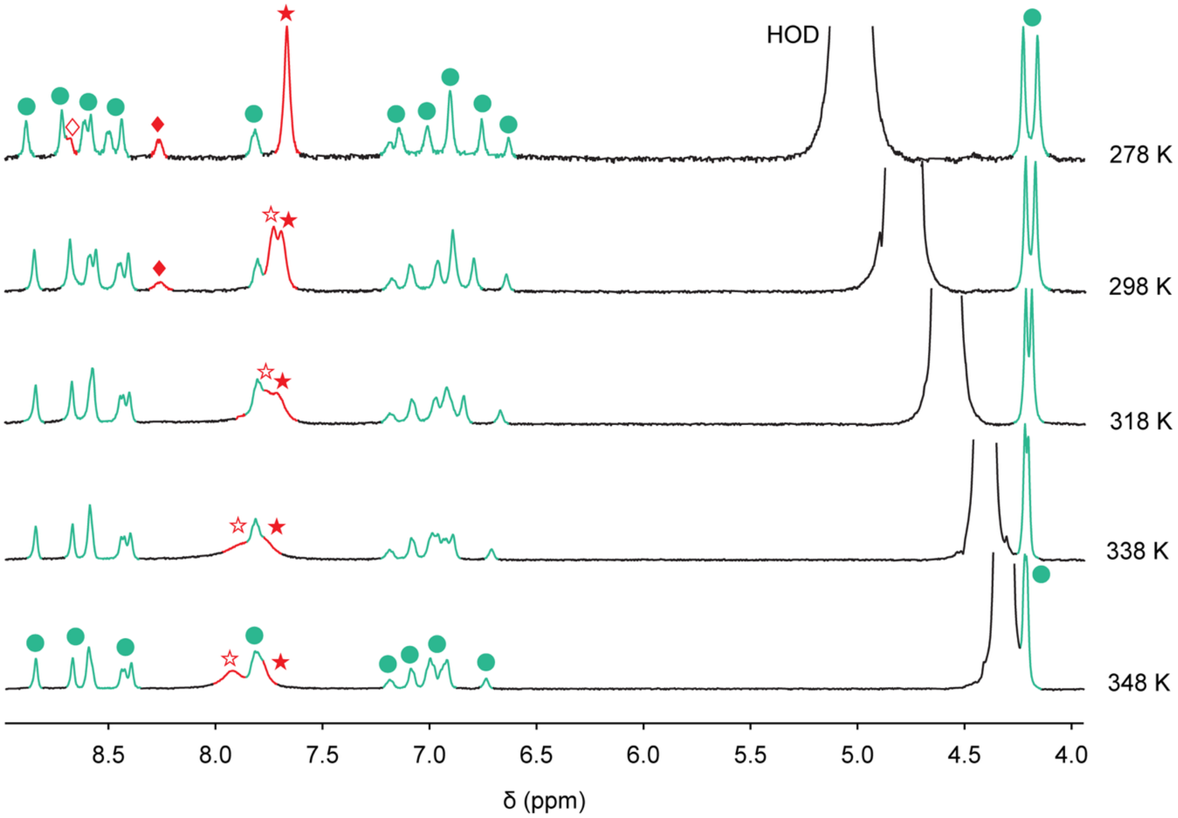

Figure S41 | Variable temperature ^{1}H NMR spectra of 2·TFA between 278 K and 348 K in D_2O (500 MHz, 0.5 mM) in the presence of 5.5 equiv. of sodium 4,4'-biphenyldisulfonate G4. No switch was observed in the conditions.

10. ¹H and ¹³C NMR spectra of the synthesized compounds

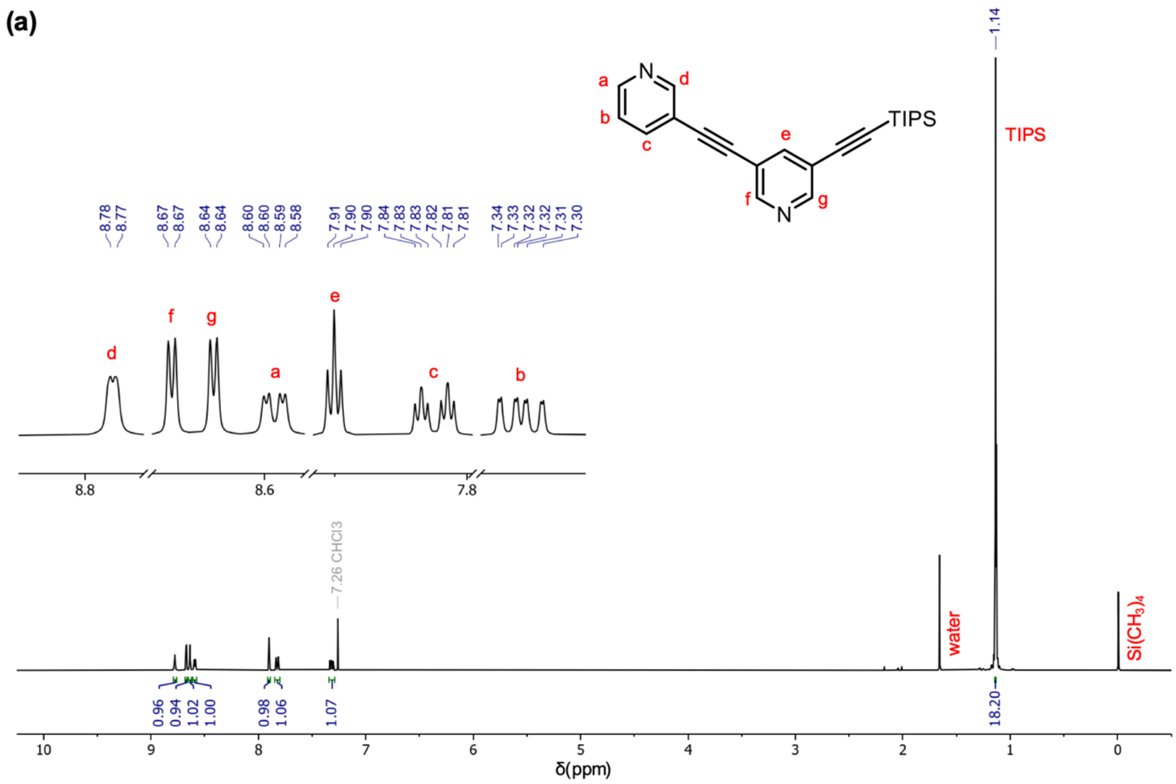

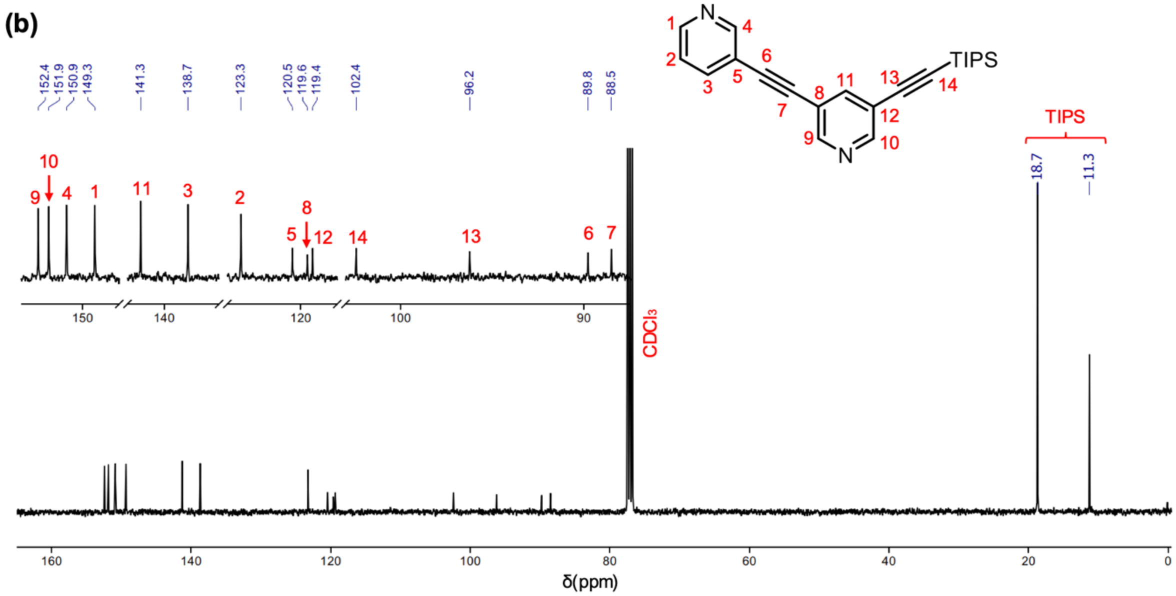

Figure S42 | NMR characterization of S2. a, ^{1}H NMR (400 MHz, $CDCl_3$ + 0.03 % tetramethylsilane, 293 K) and **b,** ^{13}C NMR (100 MHz, $CDCl_3$ + 0.03 % tetramethylsilane, 293 K).

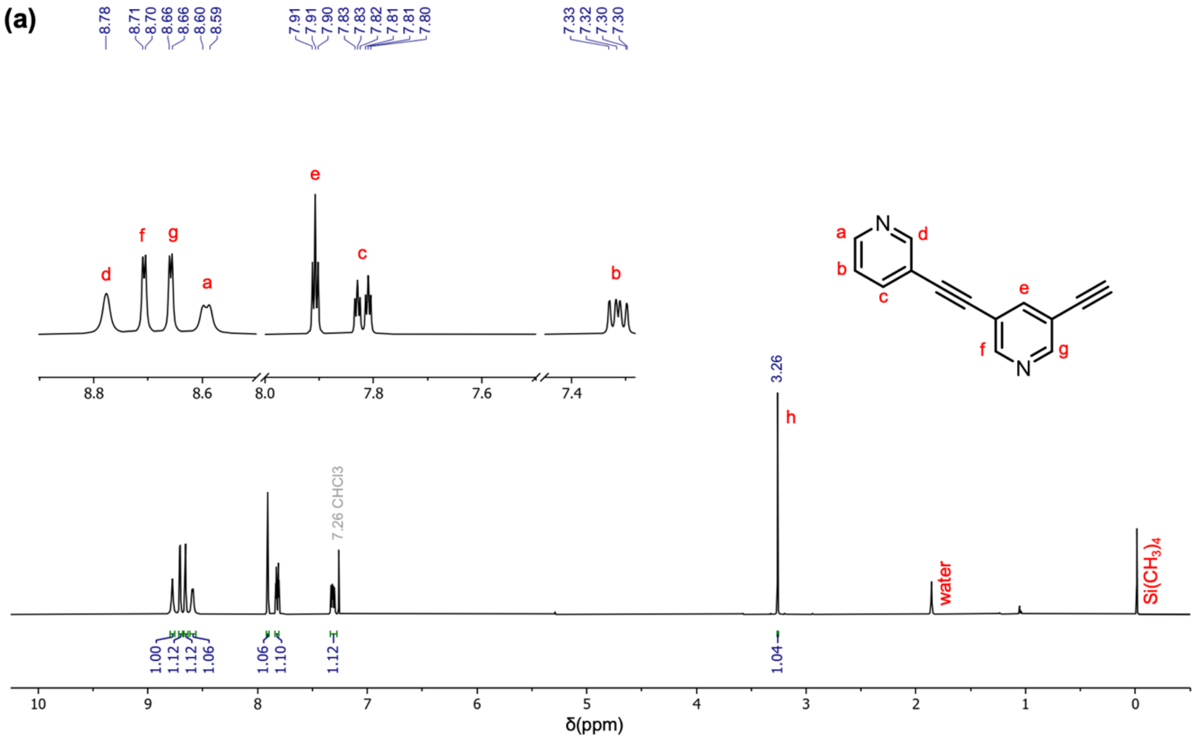

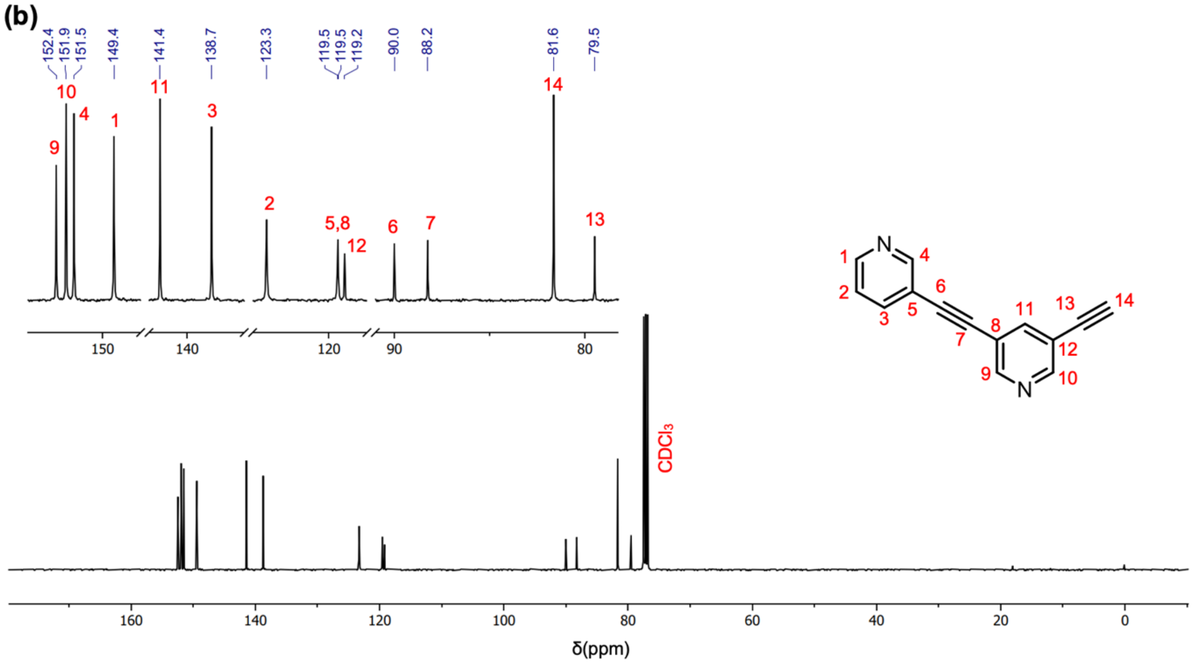

Figure S43. | NMR characterization of S3. a, ^{1}H NMR (400 MHz, $CDCl_3$ + 0.03 % tetramethylsilane, 293 K) and **b,** ^{13}C NMR (100 MHz, $CDCl_3$ + 0.03 % tetramethylsilane, 293 K).

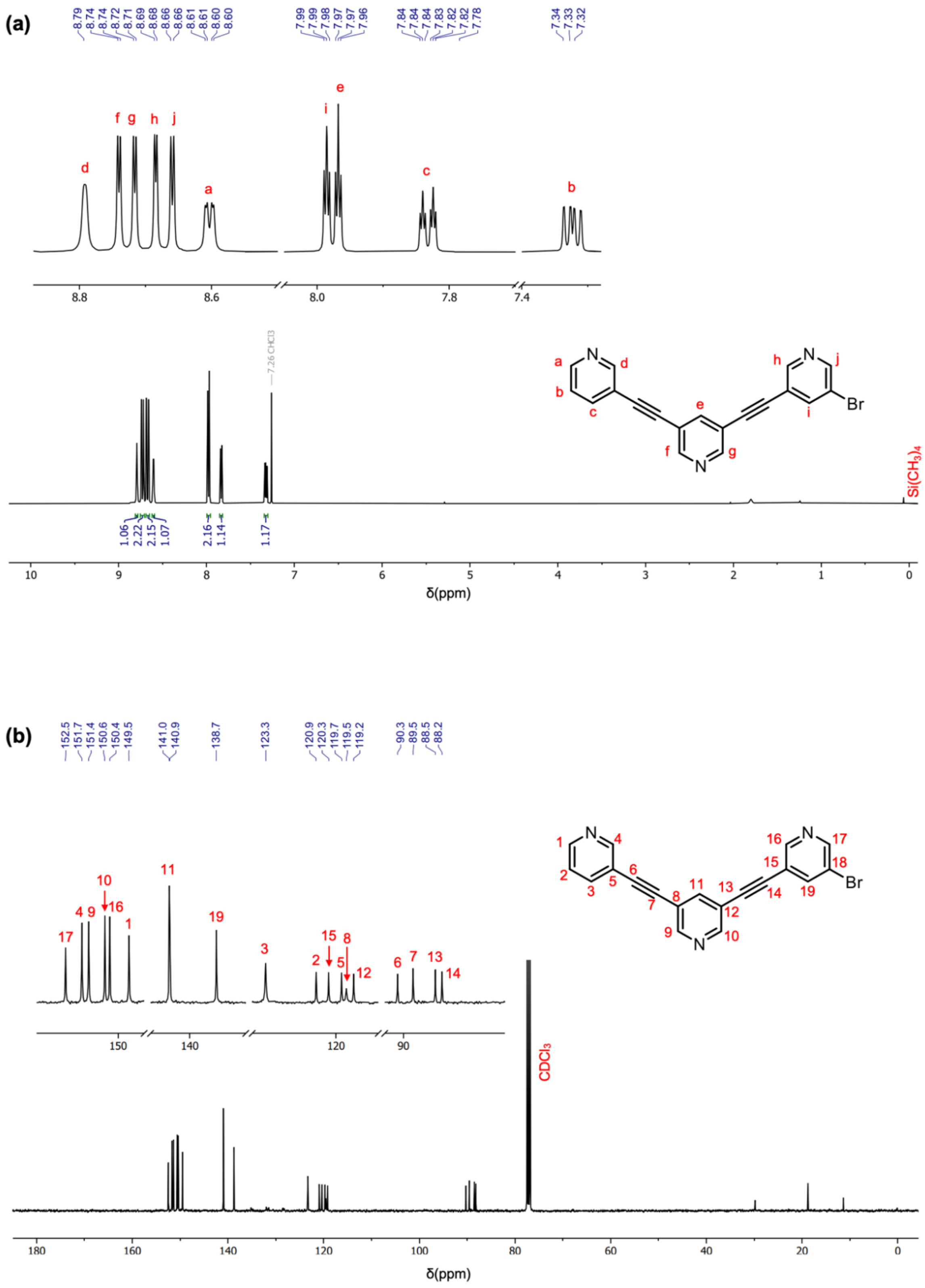

Figure S44 | NMR characterization of S4. a, ^{1}H NMR (400 MHz, $CDCl_3$ + 0.03 % tetramethylsilane, 293 K) and **b,** ^{13}C NMR (100 MHz, $CDCl_3$ + 0.03 % tetramethylsilane, 293 K).

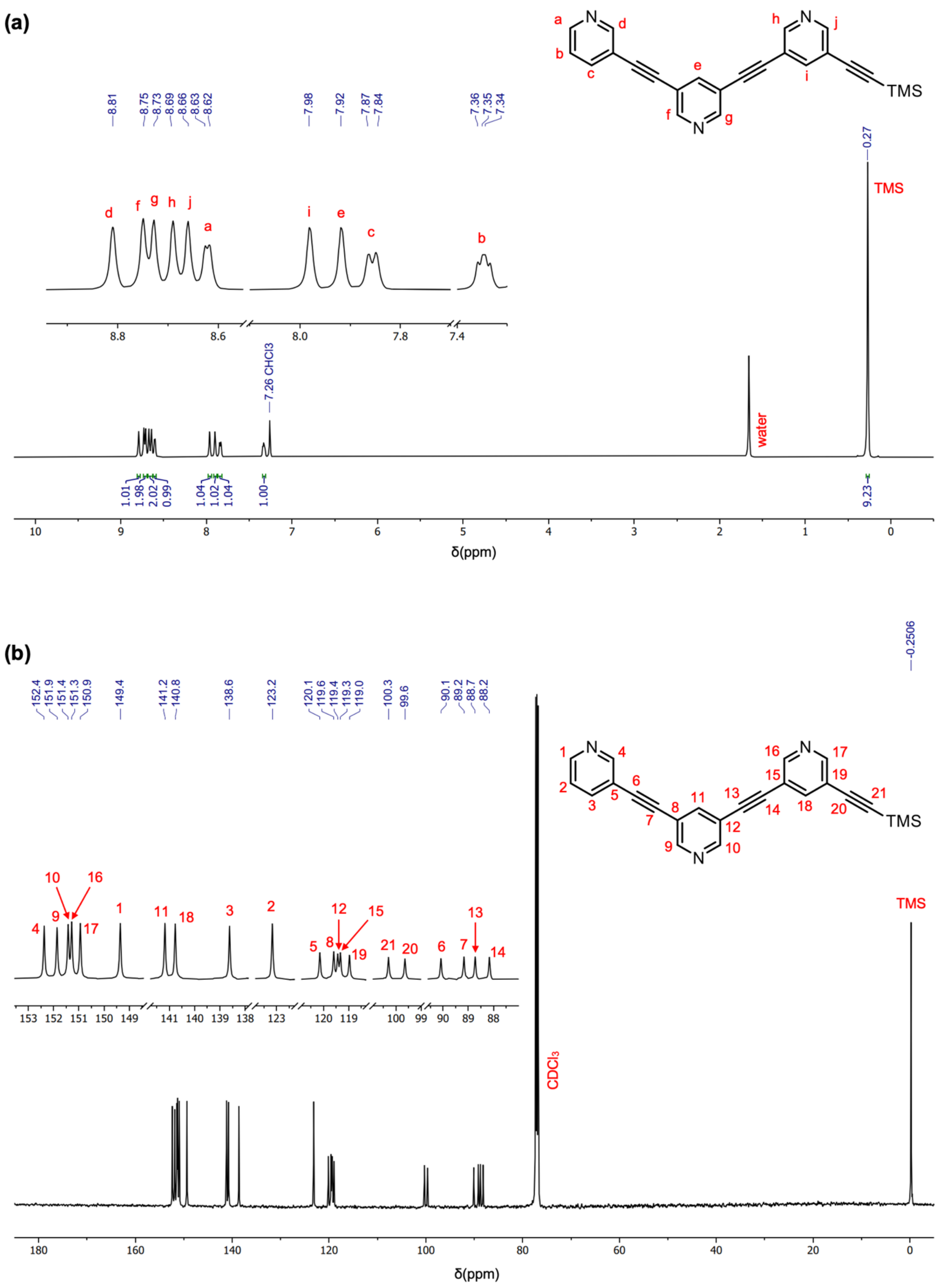

Figure S45 | NMR characterization of S5. a, ^{1}H NMR (500 MHz, $CDCl_3$, 298 K) and **b,** ^{13}C NMR (126 MHz, $CDCl_3$, 298 K).

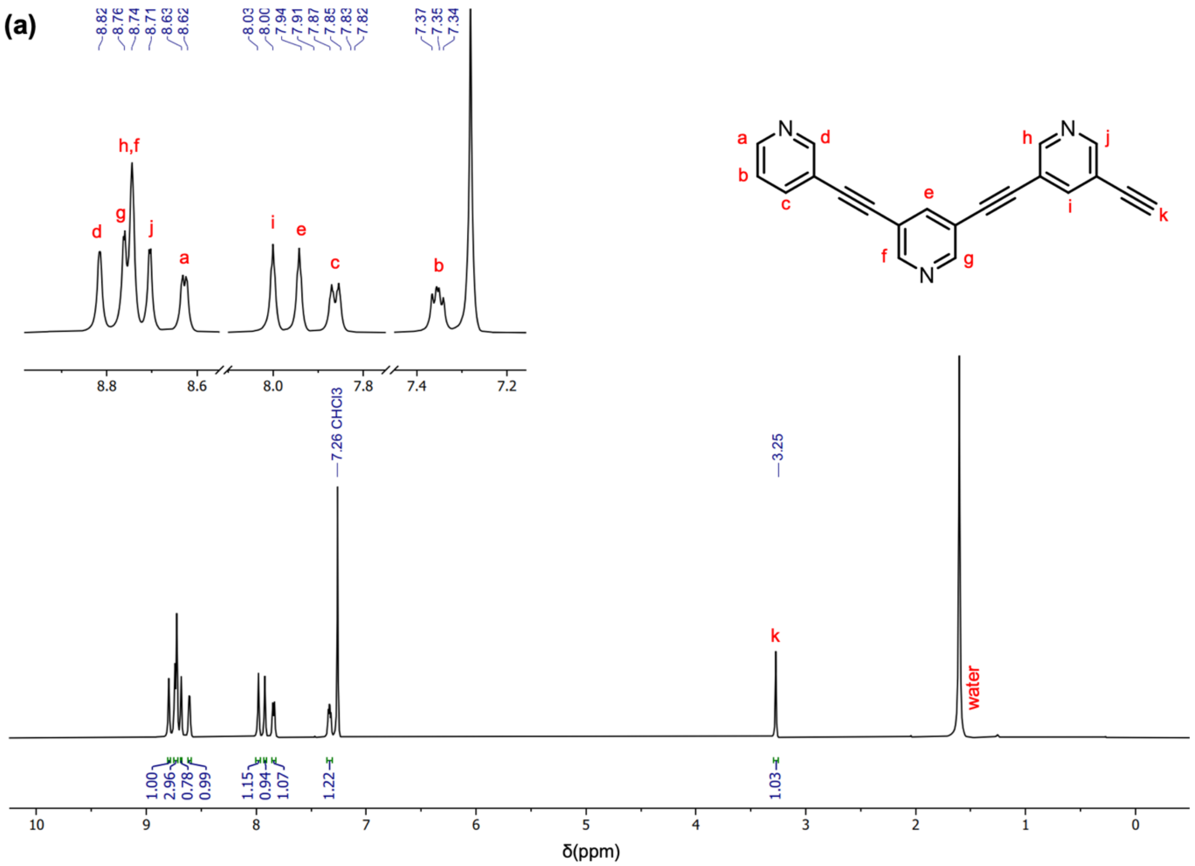

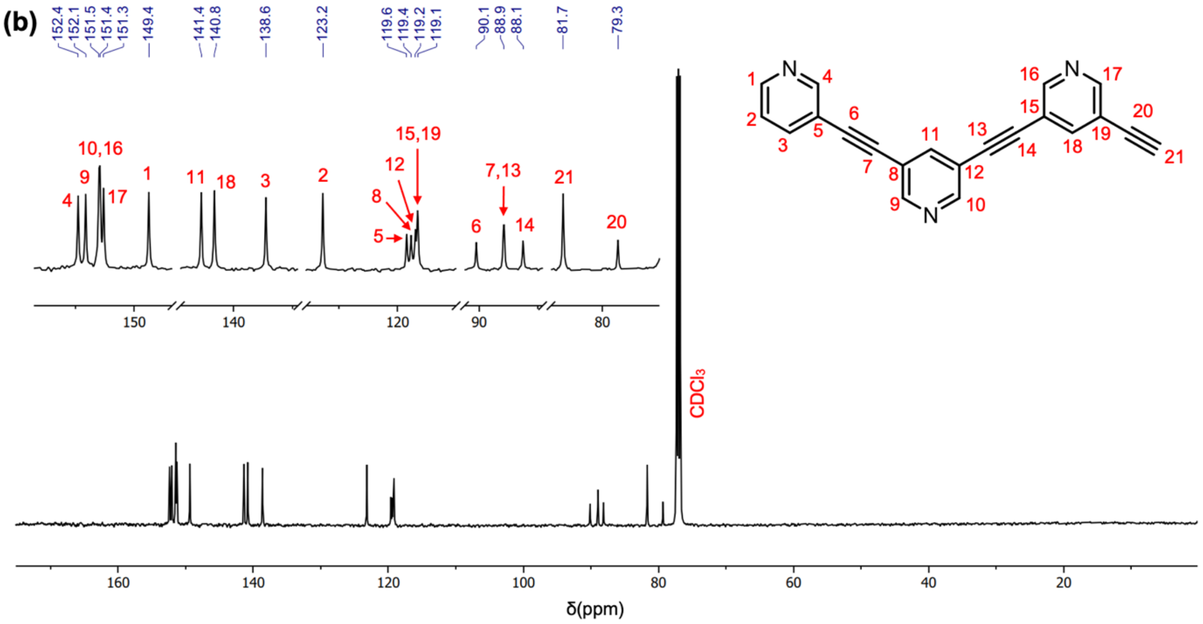

Figure S46 | NMR characterization of S6. a, ^{1}H NMR (500 MHz, $CDCl_3$, 298 K) and **b,** ^{13}C NMR (126 MHz, $CDCl_3$, 298 K).

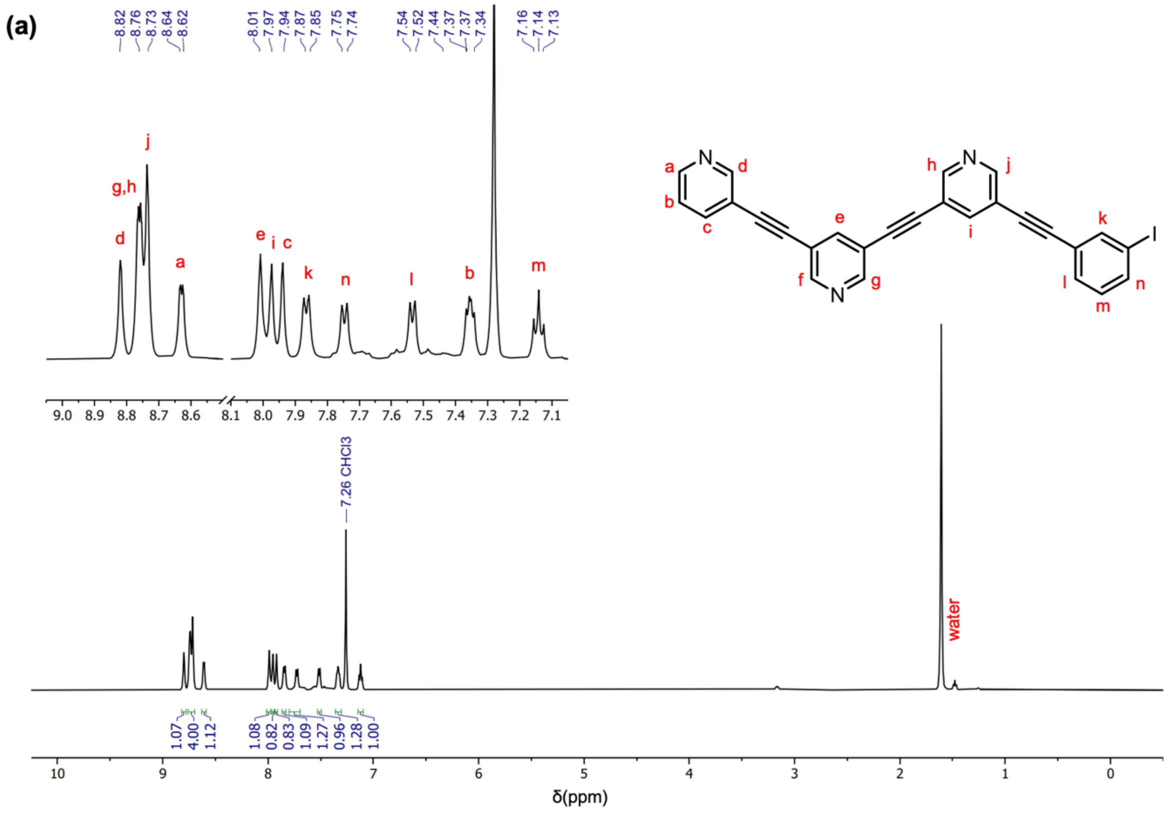

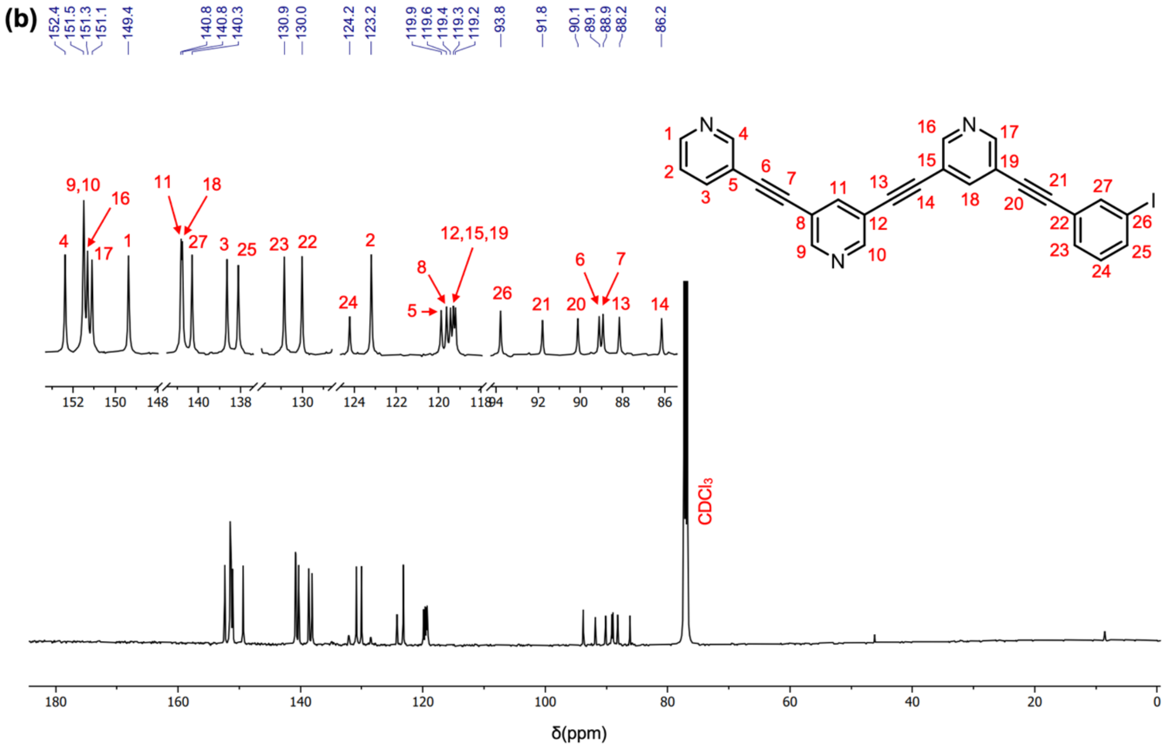

Figure S47 | NMR characterization of S7. a, ^{1}H NMR (500 MHz, $CDCl_3$, 298 K) and **b,** ^{13}C NMR (126 MHz, $CDCl_3$, 298 K).

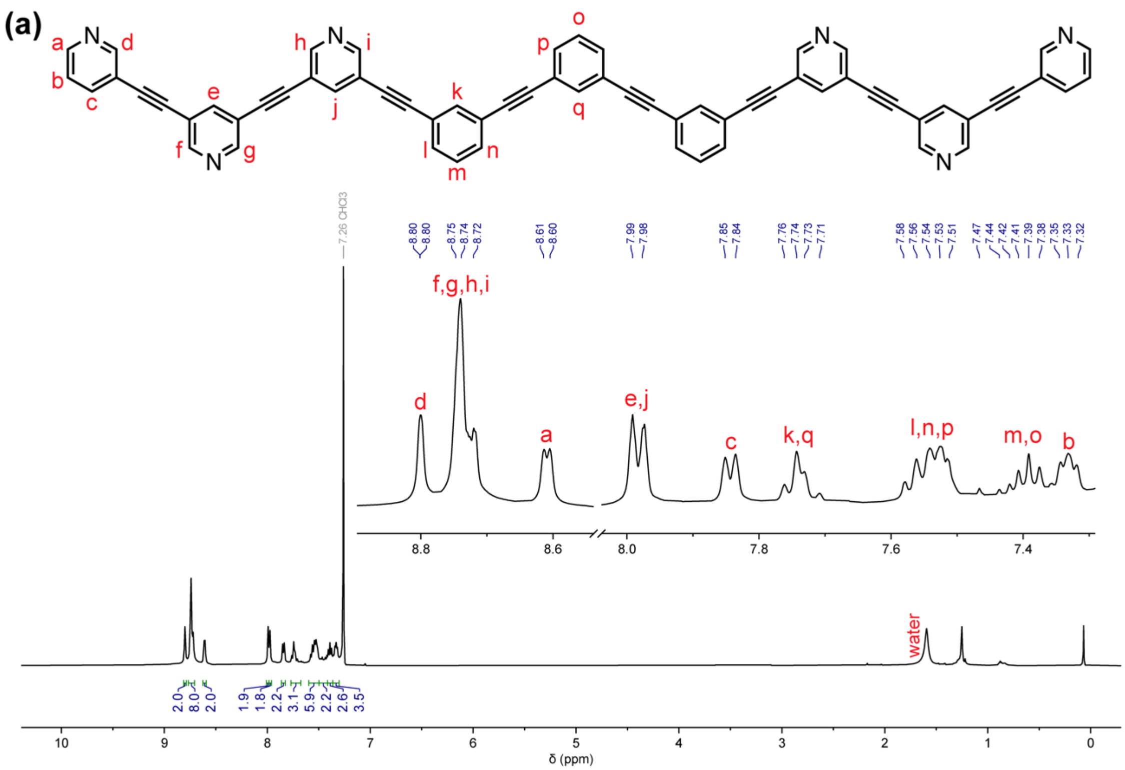

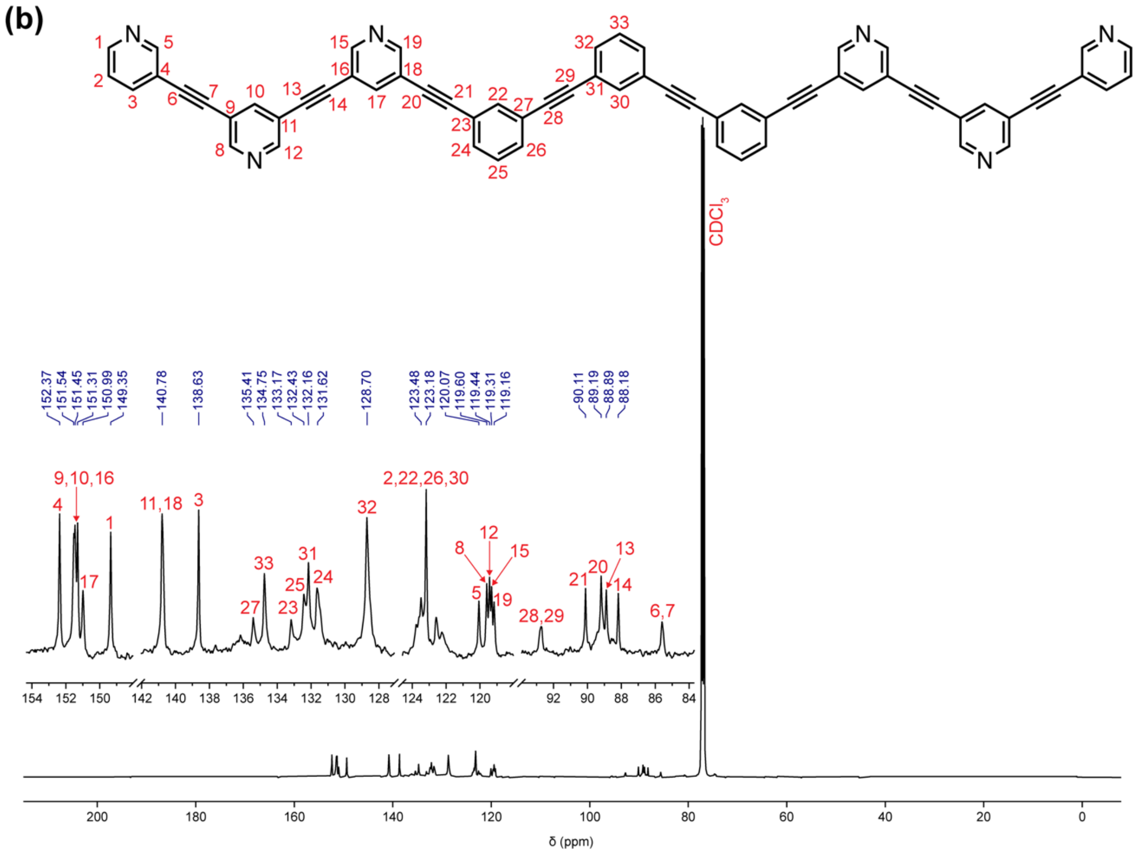

Figure S48 | NMR characterization of S8. a, ^{1}H NMR (500 MHz, $CDCl_3$, 298 K) and **b,** ^{13}C NMR (126 MHz, $CDCl_3$, 298 K).

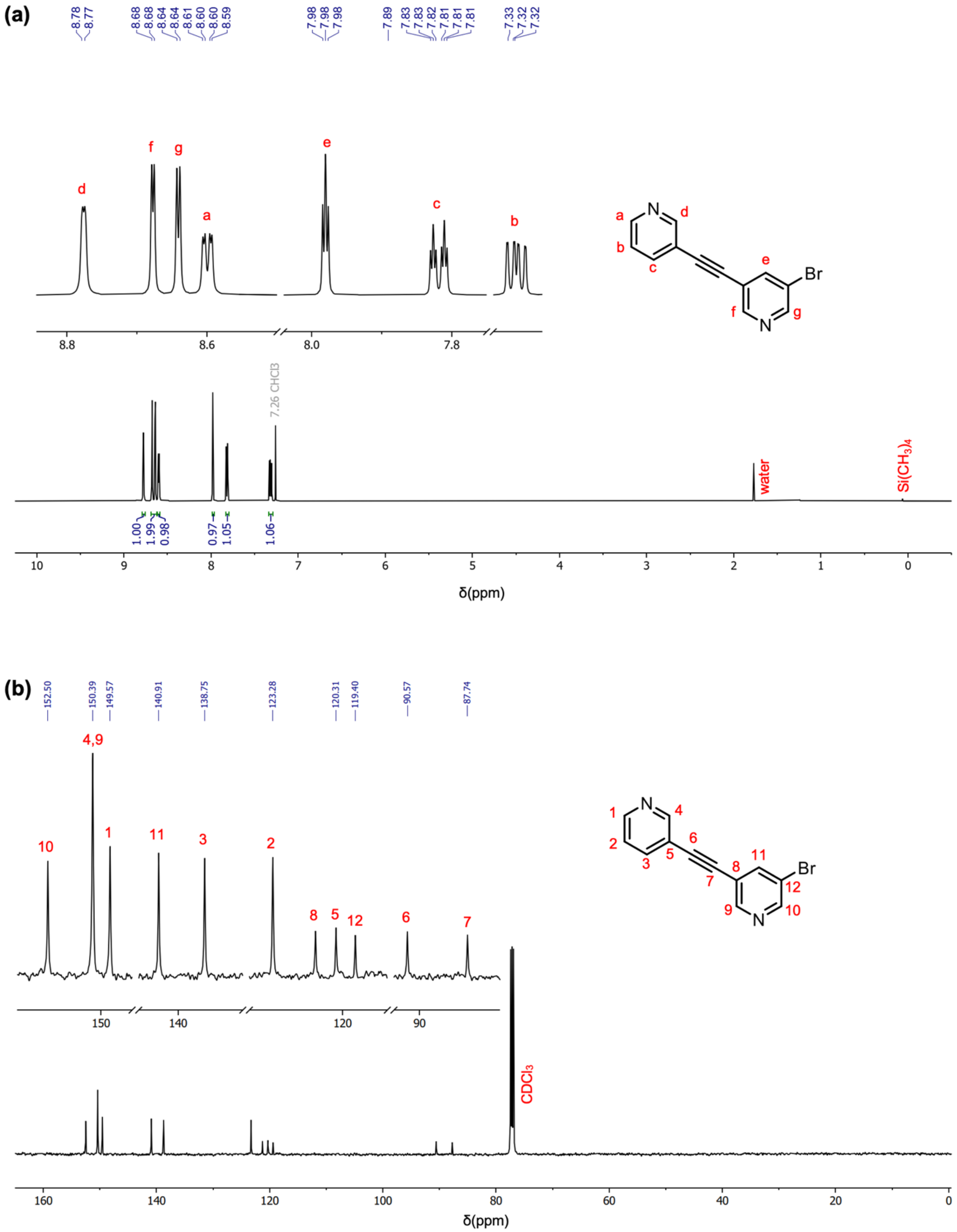

Figure S49 | NMR characterization of S12. a, ^{1}H NMR (400 MHz, $CDCl_3$ + 0.03 % tetramethylsilane, 293 K) and **b,** ^{13}C NMR (100 MHz, $CDCl_3$+ 0.03 % tetramethylsilane, 293 K).

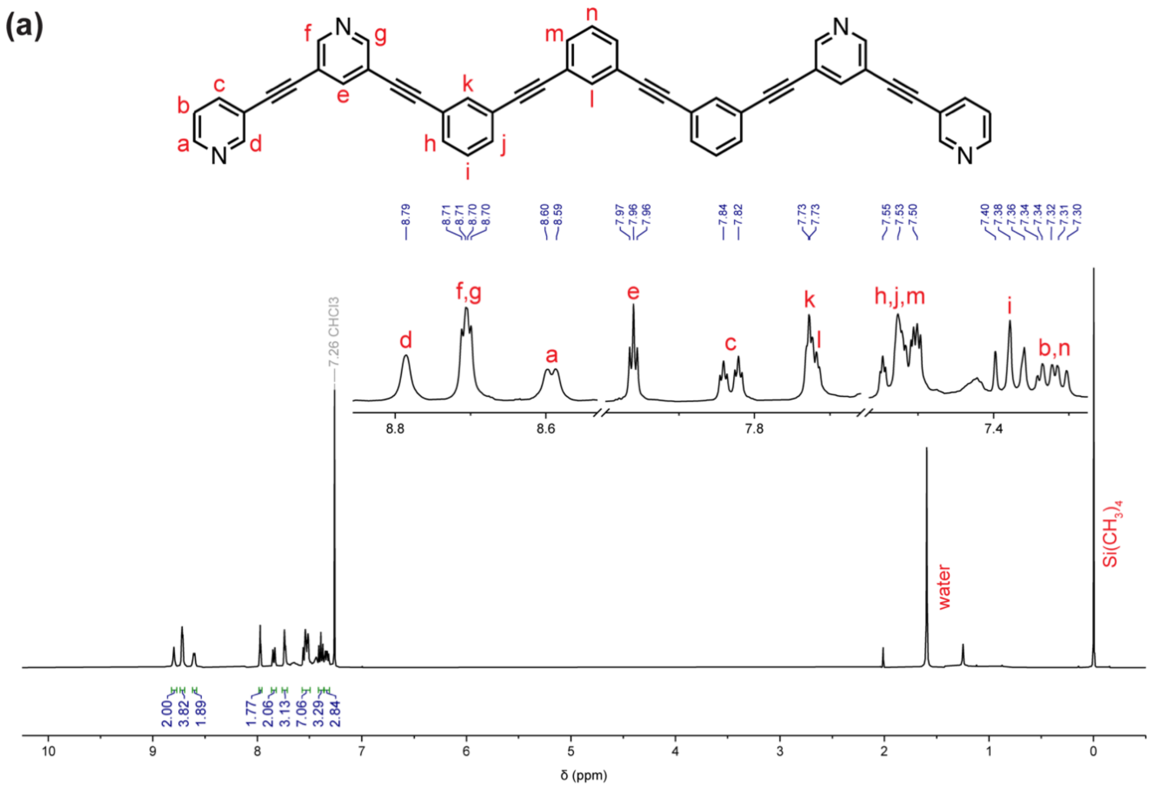

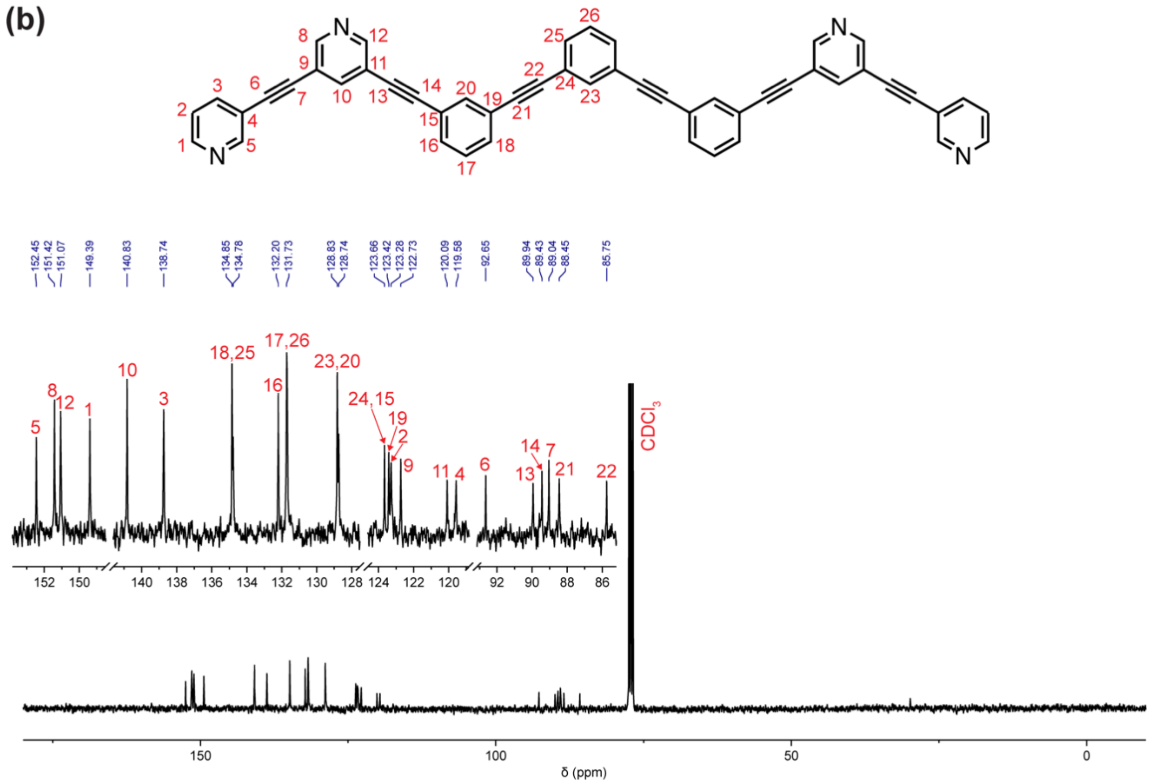

Figure S50 | NMR characterization of S13. a, ^{1}H NMR (400 MHz, $CDCl_3$ + 0.03 % tetramethylsilane, 293 K) and **b,** ^{13}C NMR (100 MHz, $CDCl_3$+ 0.03 % tetramethylsilane, 293 K).

11. DFT calculations

All geometries of (**1**)$_2$, (**2**)$_2$, (**2**)$_3$, (**2**)$_3$⊃**G2**, and (**2**)$_3$⊃**G4** were optimized without symmetry constraints. Initial structures were manually constructed and pre-optimized using Grimme's GFN2-xTB program.[5] The final re-optimizations were performed at the BP86-D4-COSMO/def2-TZVP[6-8] level of theory using the TURBOMOLE 7.7 program suite in a water solvent.[9] The solvent effects were modeled using the COSMO model[10] with the default TURBOMOLE parameters. The D4 dispersion correction[7] was included to account for crucial London dispersion forces, which are essential for the stabilization of these supramolecular complexes, given the dominance of stacking interactions. Cartesian coordinates for all DFT minimized structures are provided in the file "Supplementary Data 1".

Calculations of the helix cavity volume were performed using Swiss-PdbViewer.[11] Since the helices do not form fully enclosed cavities, we followed the standard procedure for open cavities by capping the ends with a triethynylbenzene unit to define the volume boundaries.

Supplementary Table S1 | Cavity volumes of multistranded helices calculated with Swiss-PdbViewer.

Helix	Volume (Å^3)	Interior volume (Å^3)
Double helix (**1**)$_2$	3022	378
Double helix (**2**)$_2$	2314	222
Triple helix (**2**)$_3$	3470	469

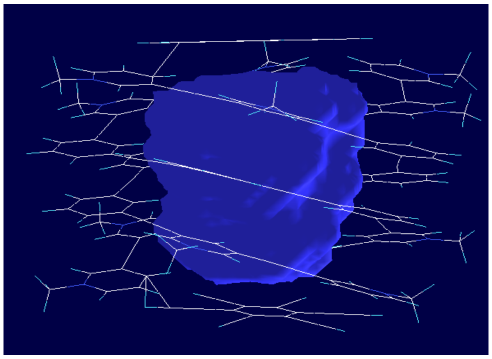

Figure S51 | Cavity volume of triple (2)$_3$ capped with a triethynylbenzene molecule.

12. References

1. Dubbaka, S. R., Kienle, M., Mayr, H. & Knochel, P. Copper(I)-Mediated Oxidative Cross-Coupling between Functionalized Alkynyl Lithium and Aryl Magnesium Reagents. *Angew. Chem. Int. Ed.* **46**, 9093–9096 (2007).
2. Moreno, J., Grubert, L., Schwarz, J., Bléger, D. & Hecht, S. Efficient Sensitized Z→E Photoisomerization of an Iridium(III)-Azobenzene Complex over a Wide Concentration Range. *Chem. – Eur. J.* **23**, 14090–14095 (2017).
3. Leung, S. Y.-L., Tam, A. Y.-Y., Tao, C.-H., Chow, H. S. & Yam, V. W.-W. Single-Turn Helix–Coil Strands Stabilized by Metal···Metal and π–π Interactions of the Alkynylplatinum(II) Terpyridyl Moieties in meta-Phenylene Ethynylene Foldamers. *J. Am. Chem. Soc.* **134**, 1047–1056 (2012).

4 BindFit v0.5 | Supramolecular. http://app.supramolecular.org/bindfit/.

5. Bannwarth, C., Ehlert, S. & Grimme, S. GFN2-xTB—An Accurate and Broadly Parametrized Self-Consistent Tight-Binding Quantum Chemical Method with Multipole Electrostatics and Density-Dependent Dispersion Contributions. *J. Chem. Theory Comput.* **15**, 1652–1671 (2019).
6. Becke, A. D. Density-functional exchange-energy approximation with correct asymptotic behavior. *Phys. Rev. A* **38**, 3098–3100 (1988).
7. Caldeweyher, E. *et al.* A generally applicable atomic-charge dependent London dispersion correction. *J. Chem. Phys.* **150**, 154122 (2019).
8. Weigend, F. & Ahlrichs, R. Balanced basis sets of split valence, triple zeta valence and quadruple zeta valence quality for H to Rn: Design and assessment of accuracy. *Phys. Chem. Chem. Phys.* **7**, 3297–3305 (2005).
9. TURBOMOLE V7.7. A development of University of Karlsruhe and Forschungszentrum Karlsruhe GmbH, 1989–2007; TURBOMOLE GmbH: Karlsruhe, Germany (2022).
10. Klamt, A. & Schüürmann, G. COSMO: a new approach to dielectric screening in solvents with explicit expressions for the screening energy and its gradient. *J. Chem. Soc. Perkin Trans. 2* 799–805 (1993).

11 Swiss-PdbViewer. https://spdbv.unil.ch/.
